# FTO/IGF2BP2-mediated N6 methyladenosine modification in invasion and metastasis of thyroid carcinoma via CDH12

**DOI:** 10.1038/s41419-024-07097-4

**Published:** 2024-10-08

**Authors:** Zuyao Chen, Xiaolin Zhong, Min Xia, Chang Liu, Weiqiang Tang, Gaohua Liu, Yan Yi, Yinping Guo, Qingshan Jiang, Xuyu Zu, Jing Zhong

**Affiliations:** 1 Clinical Medical Research Center, The First Affiliated Hospital, Hengyang Medical School, University of South China, 421001 Hengyang, Hunan China; 2 Department of Otorhinolaryngology, The First Affiliated Hospital, Hengyang Medical School, University of South China, 421001 Hengyang, Hunan China; 3 Department of Endocrinology and Metabolism, The First Affiliated Hospital, Hengyang Medical School, University of South China, 421001 Hengyang, Hunan China; 4 Department of Endocrinology and Metabolism, The First People’s Hospital of Chenzhou, The First School of Clinical Medicine, University of Southern Medical, Guang Zhou Shi, 510515 China; 5 Institute of Cancer Research, The First Affiliated Hospital, Hengyang Medical School, University of South China, 421001 Hengyang, Hunan China

**Keywords:** Cancer, RNA

## Abstract

Epigenetic reprogramming plays a critical role in cancer progression of cancer, and N6-methyladenosine (m6A) is the most common RNA modification in eukaryotes. The purpose of this study was to explore the related modification mode of m6A regulator construction and evaluate the invasion and migration of thyroid cancer. Our results showed that m6A levels were significantly increased in papillary thyroid cancer (PTC) and anaplastic thyroid cancer (ATC) samples, which may have been induced by the down-regulation of demethylase fat mass and obesity-associated gene (FTO). Moreover, FTO inhibited PTC and ATC invasion and metastasis through the epithelial-to-mesenchymal transition (EMT) pathway in vivo and in vitro. Mechanistically, an m6A-mRNA epitranscriptomic microarray showed that Cadherin 12 (CDH12) is the key target gene mediated by FTO in an m6A-dependent manner. CDH12 promotes invasion and metastasis through the EMT pathway in thyroid cancer, both in vivo and in vitro. Furthermore, we found that insulin-like growth factor 2 mRNA-binding protein 2 (IGF2BP2) is an important m6A reading protein, that regulates the stability of CDH12 mRNA and mediates EMT progression, thereby promoting the invasion and metastasis of PTC and ATC. Thus, FTO, IGF2BP2 and CDH12 may be effective therapeutic targets for PTC and ATC with significant invasion or distant metastasis.

**Figure:**
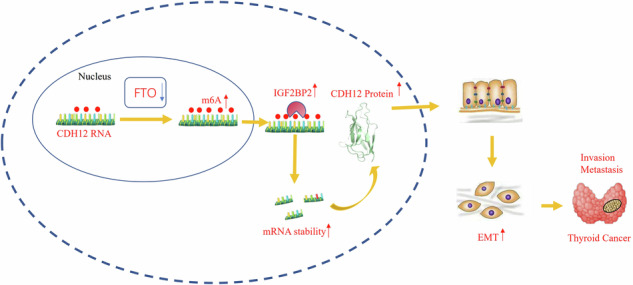
Schematic summary of FTO-IGF2BP2 axis in modulation of CDH12 mRNA m6A and upregulation of CDH12 expression in the invasion and metastasis of thyroid carcinoma.

Schematic summary of FTO-IGF2BP2 axis in modulation of CDH12 mRNA m6A and upregulation of CDH12 expression in the invasion and metastasis of thyroid carcinoma.

## Introduction

Thyroid cancer (TC) is the most common endocrine malignancy worldwide, of which papillary thyroid cancer (PTC) is the most prevalent subtype, accounting for approximately 90% of all thyroid cancer cases [1], and anaplastic thyroid cancer (ATC) is the most lethal subtype associated with rapid progression and poor prognosis [2]. Although surgical resection combined with radioactive iodine therapy and thyroid-stimulating hormone suppression therapy is recommended for PTC treatment, some advanced, invasive, and metastatic PTC do not respond to these treatments and have high mortality rates [3, 4]. In addition, there is currently no effective therapy for ATC because of its high capacity for invasion and metastasis [2]. Therefore, it is important to elucidate the molecular mechanisms involved in the invasion and metastasis of PTC and ATC, and to search for a potential therapeutic strategy.

N6-methyladenosine (m6A) modification is a type of eukaryotic RNA modification [5] that has been shown to play a regulatory role in numerous human diseases, especially in cancer progression [5], such as in lung [6], endometrial [7], breast [8], and liver cancers [9]. Several enzymes catalyse the m6A modification, such as the m6A methyltransferase complex, also known as the m6A ‘writer’, which includes methyltransferase-like 3/14 (METTL3/14), Wilm’s tuner 1-associated protein (WTAP) and Vir-like m6A methyltransferase-associated protein (KIAA1429) [10]. The enzyme that removes m6A is called demethylase, also known as the m6A ‘eraser’, which is mainly composed of fat mass and obesity-associated protein (FTO) [11] and Alk B homolog 5 (ALKBH5) [12]. The enzyme that recognises m6A and regulates the functions of activated m6A is called the m6A ‘reader’, which referred to as YT521-B homology (YTH) domain family (YTHDF1-3 and YTHDC1-2), insulin-like growth factor 2 mRNA-binding proteins (IGF2BP1/2/3), and eukaryotic initiation factor 3 (eIF3) [13]. The expression of these enzymes influences m6A levels [14], and many studies have demonstrated the active role of m6A in tumourigenesis and tumour progression through the regulation of mRNA methylation of oncogenes or tumour suppressors [15]. Evidence has shown that METTL3, FTO, YTHDF2, and IGF2BP2 participate in the progression of thyroid carcinoma [16]. Thus, understanding the role of m6A and the enzymes in TC is important for determining the most effective therapeutic strategy for the disease.

The epithelial-to-mesenchymal transition (EMT) is a process by which cancer cells lose their epithelial properties and acquire stem cell-like invasive qualities which increase their capacity for local invasion and metastasis [17]. It is also thought to occur during thyroid cancer invasion and metastasis [18]. A previous study demonstrated that METTL3-mediated m6A modification is critical for EMT in gastric cancer [19], and another study verified that m6A methylation regulates the EMT in HeLa and HepG2 cancer cells [20]. Additionally, during EMT, cells exhibit both morphological and molecular alterations, as demonstrated by decreased expression of epithelial markers, including E-cadherin, and increased expression of mesenchymal markers, including N-cadherin, Vimentin and MMP9 [21]. A subtype of the N-cadherin family, N-cadherin 2, which is also called Cadherin 12 (CDH12), has been verified to play a significant role in the progression of breast cancer and bladder cancer [22, 23]. A previous study has reported that CDH12 promotes the invasion of salivary adenoid cystic carcinoma [24] and contributes to tumorigenicity in colorectal cancer by promoting migration and invasion [25]. Moreover, one study demonstrated that CDH12 influences colorectal cancer cell progression through promoting EMT by targeting Snail [26]. However, the role of CDH12 in EMT and its m6A regulatory mechanism in thyroid cancer remain largely unknown.

Herein, we examined m6A levels and the expression of m6A-related enzymes in PTC and ATC tissues and cells, and found that m6A levels were significantly increased, accompanied by the down-regulation of FTO. Moreover, FTO inhibited the invasion and metastasis of TC cells through the EMT pathway. Additionally, we demonstrated that CDH12 is the key target gene mediated by FTO and promotes EMT progression in TC. Furthermore, we verified that IGF2BP2 regulates the stability of CDH12 mRNA and mediates EMT progression in PTC and ATC. Overall, the present study showed that low expression of FTO in thyroid cancer could up-regulate the m6A modification level of CDH12 mRNA, and that the reading protein IGF2BP2 recognises the m6A modification of CDH12 mRNA and promotes its stability, thereby upregulating the expression of CDH12, inducing the EMT process, and promoting the invasion and metastasis of PTC and ATC. We provide new insights into the FTO/IGF2BP2-mediated CDH12 m6A modification and uncover a novel molecular mechanism underlying PTC and ATC.

## Materials and methods

### Cell lines

The human PTC cell line B-CPAP (CTCC-400-0087) and human ATC cell lines CAL-62 (CTCC-400-0099) and 8305 C (CTCC-400-0275) were purchased from Meisen (Zhejiang) Cell Technology Co., Ltd., and the immortalised normal follicular epithelial cell line Nthy-ori3-1 (BFN680331) was purchased from Qingqi (Shanghai) Biotechnology Development Co., Ltd. All cell lines have been identified by STR and confirmed to be free of mycoplasma contamination. Nthy-ori3-1, B-CPAP and CAL-62 cell lines were cultured in DMEM medium (Gibco, USA), while 8305 C was cultured in 1640 medium (Gibco, USA), and all the medium was supplemented with 10% foetal bovine serum (Hyclone, Thermo Fisher Scientific, USA) and 1% penicillin-streptomycin (Gibco, USA) in 5% CO_2_ at 37 °C.

### Tissues of patients

Forty paired PTC tissue samples and their adjacent noncancerous thyroid tissue samples (Nor) were obtained from the Department of Thyroid Surgery at the First Affiliated Hospital of the University of South China between 2019 and 2021. The project was approved by the Ethics Committee of the First Affiliated Hospital of the University of South China and registered in the Chinese Clinical Trial Registry (registration number: ChiCTR2200065323). Patients in the study group were required to meet the following criteria: (1) patients who had undergone thyroid surgery for the first time and had undergone lymph node dissection in the central district or neck dissection at the same time; (2) the diagnosis of TC was confirmed by intraoperative frozen section and postoperative pathology; (3) no history of chemotherapy, radiotherapy, and immunotherapy was found before operation; and (4) age ranged from 18 to 80 years. The following conditions were also excluded from the study group: (1) recurrent TC; (2) complicated with other malignant tumours; and (3) pregnant and breastfeeding women. The mean age of the patients was 38.9 years, including 35 females and 5 males. According to TNM staging, 8 cases were T1N0M0, 4 cases were T2N0M0, 14 cases were T1N1aM0, 5 cases were T2N1aM0, 2 case were T3N1aM0, 1 case was T1N1bM0, 3 cases were T2N1bM0 and 3 cases were T3N1bM0. The tissues were immediately obtained after surgery and quickly stored in a -80 °C refrigerator for further RT-PCR (forty paired) and Western Blot (seven paired) assay. Also five paired PTC tissues were embedded in paraffin and sectioned for immunohistochemical analysis. Paraffin sections of five paired ATC tissue samples and their adjacent noncancerous thyroid tissues were obtained from the First Affiliated Hospital of the University of South China between 2010 and 2021 for immunohistochemistry.

### Cell transfection

The knockdown of FTO, CDH12 and IGF2BP2 were conducted by infection with Lv-FTO shRNA (interference sequences: #1, AGCATACAACGTAACTTTG; #2, TCACCAAGGAGACTGCTATTT; #3, TCACGAATTGCCCGAACATTA), Lv-CDH12 shRNA (interference sequences: #1, AGGCAGCAAGAGTTGTATT; #2, TGGGCAACAATTCTCCTTT; #3, GCAGTATAATTTCTCCATA) and Lv-IGF2BP2 shRNA (interference sequences: #1, agCGCAAGATCAGGGAAATTG; #2, TGAAGCTGGAAGCGCATAT; #3, GAAGCATGCCGCATGATTC) (Shanghai Genechem Co., Ltd, China) in B-CPAP, CAL-62, or 8305 C cells according to the manufacturer’s protocol. The shRNA-NC was used as a control. The overexpression of FTO, CDH12, and IGF2BP2 was induced by infection with Lv-FTO, Lv-CDH12, and Lv-IGF2BP2 (Shanghai Genechem Co., Ltd., China) in B-CPAP, CAL-62, or 8305 C cells. Lentivirus particles were transfected into tumour cells in the presence of polybrene (8 µg/mL) and selected by using 2 μg/ml puromycin. All transfected cells were tested regularly by immunoblotting to ensure the efficiency of the interference.

### M6A-ELISA

The m6A RNA Methylation Quantification Kit (Colorimetric) (ab185912; Abcam) was used to detect the level of m6A modification in the total RNA of forty paired PTC tissue samples and their adjacent noncancerous thyroid tissue samples. The m6A-ELISA was performed according to the manufacturer’s protocol. Briefly, 80 μl binding solution was added to each well, the negative control, diluted positive control, and 200 ng sample (1–8 μl) RNA were separately coated on the designated wells and incubated at 37 °C for 90 min. 50 μl diluted capture antibody was added to each well at room temperature, covered, and incubated at room temperature for 60 min, and further in the detection antibody solution for another 30 min. Developer and stop solutions were used to detect the signals. m6A levels were assessed by reading the absorbance of each well at a wavelength of 450 nm and were then calculated based on the standard curve.

### RNA isolation and real time-PCR

Total RNA was extracted by TRIzol® reagent (CWBIO, Beijing, China). The concentration of RNA was detected using Nanodrop (Thermo Scientific), and RNA purity was detected through the A260 nm/A280 nm absorption ratio. cDNA was synthesised using the RevertAid First Strand cDNA Synthesis Kit (Fermentas). Gene expression was determined by the Roche LC480 real-time PCR system with TB Green Premix Ex Taq II (Takara). Primers were designed with Primer 3 software (Supplementary Table 1). The PCR cycling conditions were 30 s at 95 °C followed by 40 cycles at 95 °C for 5 s and 60 °C for 45 s. The relative gene expression was measured through the 2^−△△Ct^ method. For tissues, *n* = 40 paired, while for cell lines, *n* = 6 repetitions.

### RNA co-immunoprecipitation (RIP)

Protein was extracted with IP lysis solution including PMSF from CAL-62 cells. Then, 2 µg of the rabbit m6A (Abclonal), IGF2BP2 (Proteintech), and IgG (Proteintech) antibodies were added to the lysis solution overnight at 4 °C on the rotating instrument, the experiment shown was replicated four times. The next day, the resuspending magnetic beads were added to the lysis solution for 5 h at 4 °C on a rotating instrument. After washing three times with 500 μl RIPA wash buffer, Trizol^®^ reagent (CWBIO) was used to extract the RNA, and the amount of CDH12 mRNA was detected by RT-qPCR after reverse transcription.

### Western blotting

The proteins in tissues or cells were extracted using the protein cleavage reagent RIPA, including PMSF. Protein concentration was detected using a BCA Assay Kit (CWBIO). The protein extracts were then separated by sodium dodecyl sulfate-polyacrylamide gel electrophoresis at 100 V for 2 h and transferred to PVDF membranes (Millipore) at 200 mA for 2 h. Then, the membranes were blocked with 5% nonfat milk for 2 h and incubated with primary antibodies (Supplemental Table 2). After washing three times in 0.02 M Tween-Tris buffered solution, the membranes were incubated with horseradish peroxidase (HRP)-conjugated donkey anti-rabbit or donkey anti-mouse polyclonal secondary antibodies (1:1000, CWBIO) for 2 h. Signals were detected using an enhanced chemiluminescence (ECL) system (CWBIO). The protein levels were quantified by densitometry using the NIH ImageJ software (NIH, Bethesda, MD, USA). For tissues, *n* = 7 paired. For cell lines, *n* = 3 repetitions.

### Immunohistochemistry

The paraffin sections (*n* = 5 paired) were soaked in xylene overnight and dewaxed in a graded ethanol series. Endogenous peroxidases were removed from the sections using 3% H_2_O_2_. Antigen repair was completed with antigen repair solution at 95 °C for 20 min. The sections were then blocked in 5% normal sheep serum with 0.1% Triton for 2 h and incubated with anti-FTO (Proteintech, 1:500), anti-CDH12 (Abclonal, 1:200), anti-IGF2BP2 (Proteintech, 1:500) and anti-m6A (Abclonal, 1:200) at room temperature for 2 h, followed by incubation at 4 °C overnight. The secondary antibodies used were biotinylated goat anti-rabbit or goat anti-mouse IgG (Proteintech, 1:200). Diaminobenzidine tetrahydrochloride (ZSGB-BIO, Beijing, China) was used as a peroxidase substrate. Three washes with 0.01 M PBS were carried out between the above incubations, except for the blocking step. Images were captured using a digital camera that was attached to a microscope (Olympus, Tokyo, Japan). Under a microscope, brown or yellow granules in the cytoplasm or nucleus indicate positive staining of immunohistochemistry.

### m6A-mRNA epitranscriptomic microarray

CAL-62 cells transfected with Lv-shFTO were used for m6A-mRNA epitranscriptomic microarray assay. Total RNA (*n* = 3 repetitions) was prepared and microarray hybridisation was performed according to Arraystar’s standard protocols. Briefly, total RNAs were immunoprecipitated using an anti-m6A antibody. The modified RNAs were eluted from the immunoprecipitated magnetic beads as the ‘IP’. The unmodified RNAs were recovered from the supernatant as ‘Sup’. The ‘IP’ and ‘Sup’ RNAs were labelled with Cy5 and Cy3 respectively as cRNAs in separate reactions using Arraystar Super RNA Labeling Kit. The cRNAs were combined and hybridised onto Arraystar Human mRNA Epitranscriptomic Microarray (8×60 K, Arraystar). After washing the slides, the arrays were scanned in two colour channels using an Agilent Scanner G2505C. The Agilent Feature Extraction software (version 11.0.1.1) was used to analyse the acquired array images. The ‘m6A methylation level’ was calculated for the percentage of modification based on the IP (Cy5-labelled) and Sup (Cy3-labelled) normalised intensities. The ‘m6A quantity’ was calculated for the m6A methylation amount based on the IP (Cy5-labelled) normalised intensities. The ‘RNA expression level’ was calculated based on the total IP (Cy5-labelled) and Sup (Cy3-labelled) normalised intensities of RNA, and an additional quantile normalisation method of limma package was used to normalise the expression level between arrays before probes flag screening.

### m6A dot blot assay

Total RNA was extracted with Trizol reagent and denatured by heating at 65 °C for 5 min. Equal amounts of serially diluted mRNA were spotted onto a nylon membrane (Thermo Scientific) and cross-linked with 365 nm UVP for 15 min. After blocking with 5% non-fat milk for 2 h. m6A levels were measured using an anti-m6A antibody (Abclonal, A17924) in 4 °C over-night. Membranes were incubated with HRP-conjugated donkey anti-rabbit (Millipore, 1:2000) at room temperature for 2 h. Signals were measured using an ECL system (CWBIO). The membranes were incubated with a 0.02% methylene blue solution for 2 h and washed with water. *N* = 3 repetitions for cell lines.

### Transwell assay

Cell migration and invasion were determined using Transwell chambers (Corning Costar, diameter = 8 μm). The single-cell suspension (1 × 10^5^; 300 μl) diluted in serum-free medium was added to the upper chamber (with or without matrix glue), whereas the basolateral chambers were filled with 10% FBS-supplemented medium. The cells were allowed to migrate for 24 h in 5% CO2 at 37 °C. The migrated and invaded cells on the lower surface were fixed in methanol solution, stained with Giemsa dye, and observed under a microscope. The experiment shown was replicated three times. The number of cells in the three different microscopic views was evaluated to quantify cell invasion or migration.

### Wound-healing assay

For the wound-healing assay, cells subjected to different treatments were seeded into 6-well plates. When the cells formed a confluent monolayer, the middle of every well was scraped with a 10 μl sterile pipette tip. Wound images of B-CPAP and CAL-62 were acquired at 0 h, 24 h and 48 h by using a light microscope. Due to the rapid proliferation of 8305 C cells, wound images of 8305 C were only acquired at 0 h and 24 h. The experiment shown was replicated three times. ImageJ software (National Institutes of Health, Bethesda, MD, USA) was used to quantitatively detect the gap distance.

### RNA stability assays

In order to measure the influence of IGF2BP2 knockdown or over-expression on the stability of CDH12 mRNA in thyroid cancer cells, actinomycin D (Sigma-Aldrich, USA) was added at a final concentration of 5 μg/ml. Cells were collected at 0, 3, 6, and 9 h. *N* = 6 repetitions. Total RNA was extracted, and RT-qPCR was performed as described above, GAPDH was used as a loading control for normalisation. Finally, the RNA half-life was calculated.

### Mouse experiment

Five-week-old female BALB/c nude mice were obtained from Speife Biotechnology Co., Ltd. (Beijing, China). Mice were acclimated to the new environment for one week before the experiment, and randomly assigned to each group (*n* = 8 for each group). All the animals were housed in an environment with temperatures of 26–28 °C, relative humidity of 40–60%, and ventilated 10-15 times per hour. The experimental protocol was approved by the Animal Care and Use Committee of the University of South China and conformed to the National Institutes of Health Guide for the Care and Use of Laboratory Animals. The mixture of shFTO#1, shFTO#2 and shFTO#3 was used to infect CAL-62 cells, also the mixture of shCDH12#1, shCDH12#2 and shCDH12#3 was used to infect CAL-62 cells. The prepared cells were intravenously injected into nude mice via the tail vein. After 45 days of modelling, the nude mice were weighed and intraperitoneally injected with 10 μl/g of the fluorescein potassium salt solution (15 mg/ml); 5 minutes later, mice were intraperitoneally injected with 10% sodium pentobarbital (80 mg/kg), and after another 5 minutes lung luciferase activity was measured by three-dimensional imaging system of small animals (Pekinelmer, USA). Then, the lung tissues were removed and the pulmonary nodules were further counted, and the lung tumours were used for the HE assay. To avoid any bias, the researcheres quantifying the pulmonary nodules were blinded to the treatment.

### HE staining

Lung tumour tissues were fixed in 10% formaldehyde (Solarbio, Beijing, China) for 24 h and embedded in paraffin. Paraffin sections (4 μm thick) were soaked in xylene overnight and then dewaxed in a graded ethanol series. Afterwards, the sections were stained with haematoxylin solution (Servicebio, Wuhan, China) for 8 min and in hydrochloric acid-ethanol mixtures for 5 s, followed by washing for 20 min, and stained with 0.5% eosin solution (Servicebio) for 1 min. The final sections were treated with a graded ethanol series for 2 min and dehydrated with xylene for 10 min. Pathological examinations were performed using a microscope (Nikon, Japan).

### Bioinformatics analysis

The KMplot program (http://kmplot.com) and the GEPIA (http://gepia2.cancer-pku.cn) databases were used to plot the Kaplan-Meier survival curves for FTO, CDH12, and IGF2BP2.

### Statistical analysis

Statistical analyses were performed using GraphPad Prism software (version 5.0; GraphPad Prism Software, San Diego, CA, USA). The exact sample size, the statistical test used, and the results of the tests for each experiment were accorded to the requirements of statistics and showed in methods and Fig. legends. Any value > 2 standard deviation from the group mean was considered as an outlier (a criteria decided on prior to initiating the experiments) and excluded from the analysis. All data comply with the homogeneity of variance test. A paired T-test was used for statistical analysis between tumour tissues and adjacent normal tissues. An unpaired t-test was used for the statistical analysis between two independent groups. Differences among three or more groups were determined using one-way analysis of variance (ANOVA) followed by Bonferroni’s post hoc test. The differences between the four groups in the rescue experiments were analysed using two-way ANOVA. Data are presented as the mean ± SEM. *P* values < 0.05 were considered to be statistically significant.

## Results

### m6A modification was increased in thyroid carcinoma and correlated with a lower FTO expression

**Fig. 1:**
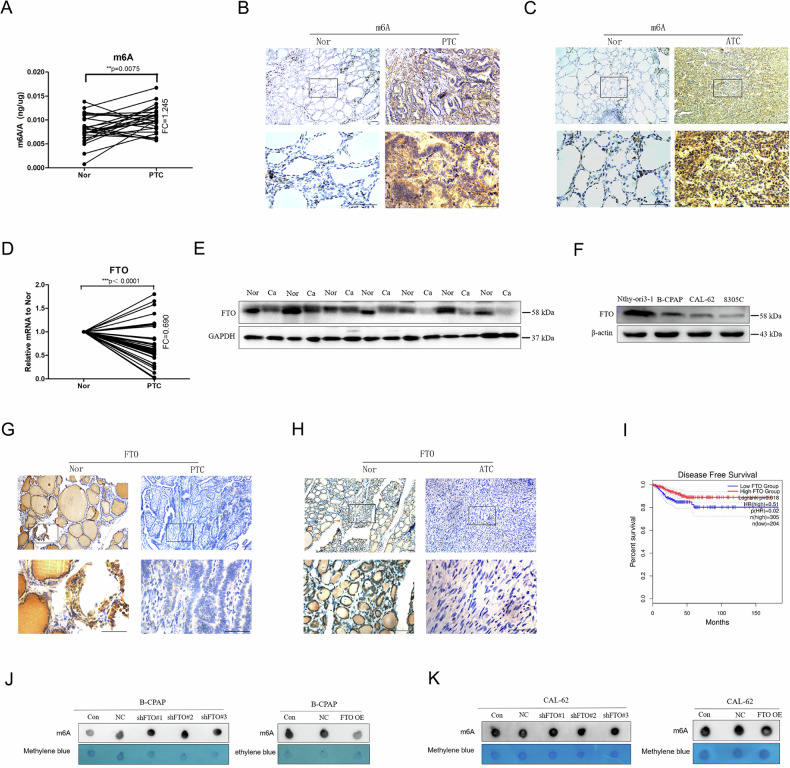
The level of m6A and expression of FTO in thyroid carcinoma. **A** m6A-ELISA analysis of m6A levels in papillary thyroid carcinoma (PTC) tissues and the adjacent normal tissues (Nor) (*n* = 40 paired). **B** Representative immunohistochemistry images of m6A-positive cells in PTC and (**C**) ATC tissues and Nor tissues (*n* = 5 paired). **D** The mRNA expression level of FTO in papillary thyroid carcinoma (PTC) tissues and Nor tissues (n = 40 paired). (**E**) The protein level of FTO in PTC and Nor tissues (*n* = 7 paired). **F** Protein level of FTO in Nthy-ori 3-1 (represents thyroid normal cells), B-CPAP (represents PTC), and CAL-62 and 8305 C (represents ATC) cell lines (*n* = 3 repetitions). **G** Representative immunohistochemistry images of FTO-positive cells in PTC and (**H**) ATC and Nor tissues (*n* = 5 paired). **I** Kaplan–Meier survival curves of disease-free survival based on FTO. **J** Representative images of m6A dot blot assay showed the effects of FTO on the level of m6A in B-CPAP and (**K**) CAL-62 cells (n = 3 repetitions). Bar=200 μm, ^*^p < 0.05, ^**^p < 0.01, ^***^p < 0.001.

M6A has been shown to play a regulatory role in cancers [5–9]. We measured m6A modifications in TC and adjacent normal tissues (Nor) and found that m6A modifications were considerably increased in both PTC and ATC tissues (Fig. 1A, B and C). Since m6A modifications are generated by methyltransferases and removed by demethyltransferases in eukaryotic RNA, we systematically analysed the mRNA levels of m6A modification enzymes in TC and Nor tissues (Fig. 1D; Supplement Fig. 1A-E). The mRNA levels of the demethyltransferases FTO and ALKBH5 decreased in PTC tissues, but FTO decreased more significantly. We further demonstrated that the protein level of FTO was considerably lower in both PTC and ATC tissues than in the Nor tissues (Fig. 1E, G and H; Supplement Fig. 1F and H). Because of the low incidence rate of ATC in the clinic, the cell lines were also used to estimate the protein level of FTO; we found that when compared with the Othy-ori3-1 cells (representing normal thyroid cells), the protein levels of FTO in B-CPAP (representing PTC), CAL-62 and 8305 C cells (representing ATC) decreased significantly, and the FTO protein levels in CAL-62 and 8305 C were lower than those in B-CPAP (Fig. 1F; Supplement Fig. 1G). In addition, m6A dot blot assays revealed that the down-regulation of FTO in B-CPAP and CAL-62 cells increased the m6A modification level of total mRNA, whereas the upregulation of FTO had the opposite effect (Fig. 1J-K). Furthermore, GEPIA2 data indicated that patients with PTC with high FTO expression had optimistic disease-free-survival (DFS) (Fig.1I). These results suggest that FTO-mediated the up-regulation of m6A modifications in PTC and ATC may play an important role in the development of thyroid carcinoma.

### FTO inhibits the invasion and metastasis of thyroid carcinoma cells in vivo and in vitro

**Fig. 2:**
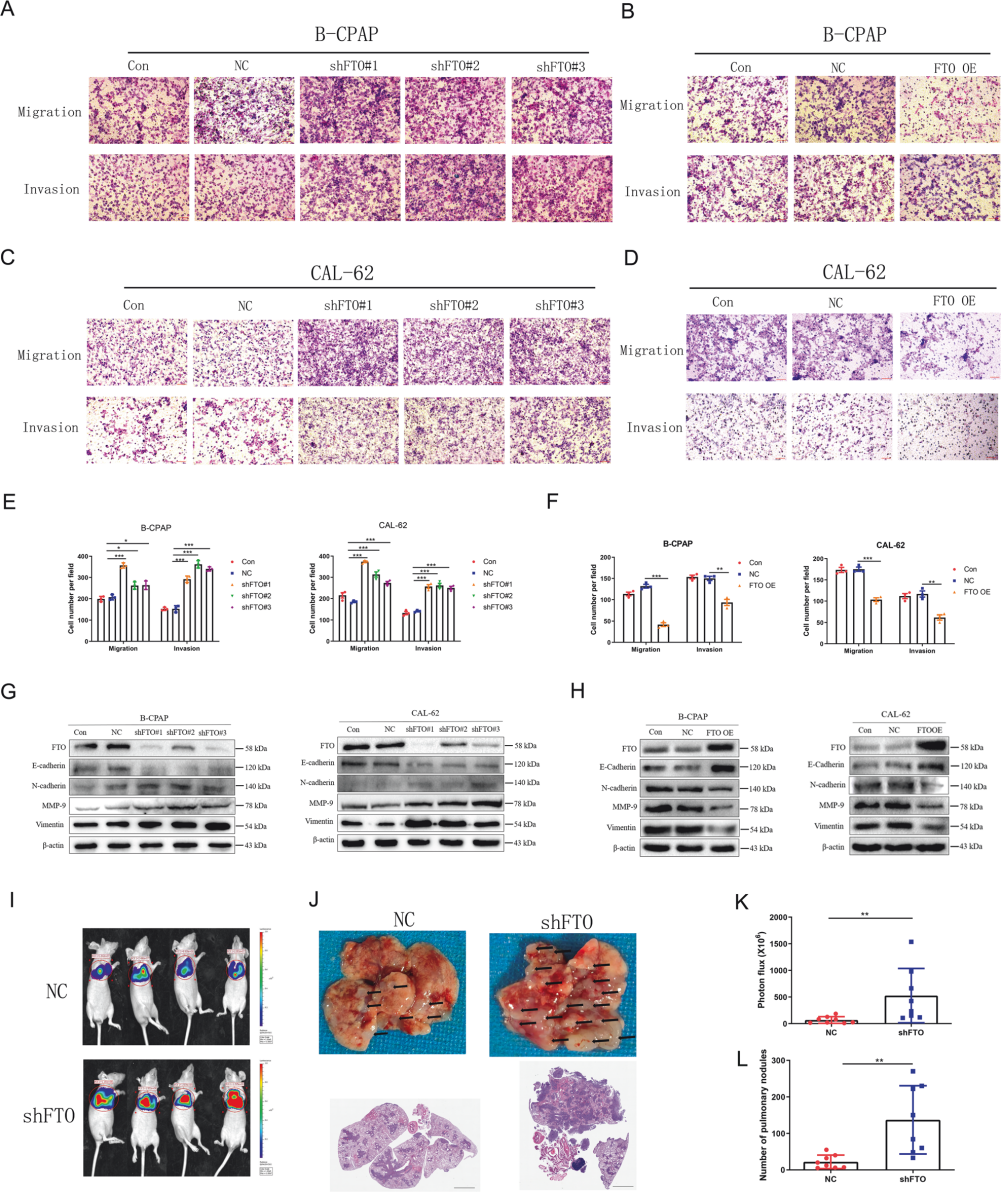
The role of FTO in thyroid carcinoma invasion and metastasis. **A, C** Transwell assay analysing the effect of FTO knockdown (shFTO) or (B, D) FTO over-expression (FTO OE) on the invasive and migration capabilities in B-CPAP and CAL-62 cells and the quantitatively analyzed (**E–F**) (*n* = 3 repetitions). **G** Influences of shFTO or (**H**) FTO OE on the protein levels of E-cadherin, N-cadherin, MMP-9, and Vimentin in B-CPAP and CAL-62 cells (*n* = 3 repetitions). **I** Luciferase activity of nude mice metastatic lung tumours were shown and (**K**) were quantitatively analyzed after CAL-62 cells were infected with shFTO and shRNA-NC virus injected (*n* = 8 mice). **J** Lung metastatic tumours of nude mice and the panoramic section HE staining results were shown and (L) were quantitatively analyzed after CAL-62 cells were infected with shFTO and shRNA-NC virus injected (*n* = 8 mice). Bar=200 μm for Transwell images and Bar=1 mm for HE images, ^*^p < 0.05, ^**^p < 0.01, ^***^p < 0.001.

Patients with thyroid carcinoma with extensive local invasion and distant metastasis frequently do not respond to standard treatments and have a worse prognosis [27]. We next investigated the role of FTO in the invasion and metastasis of thyroid carcinoma. We found that FTO mRNA levels in PTC tissues with cervical lymph node metastasis (LM + ) were lower than those in tissues without cervical lymph node metastasis (LM-) (Supplement Fig. 1I), indicating that the down-regulation of FTO may participate in the metastasis of PTC. Transwell and wound-healing assays demonstrated that FTO knockdown significantly promoted the invasion and migration capabilities of B-CPAP, CAL-62 and 8305 C cells (Fig. 2A, C and E, Supplement Fig. 2A, C, E and G), whereas FTO over-expression (FTO OE) produced the opposite functions (Fig. 2B, D and F, Supplement Fig. 2B, D, F and H). Remarkably, FTO knockdown induced down-regulation of the epithelial marker E-cadherin with concomitant up-regulation of the mesenchymal markers N-cadherin and Vimentin, and up-regulation of an important matrix metalloproteinase 9 (MMP-9) (Fig. 2G; Supplement Fig. 2I). Conversely, FTO over-expression upregulated E-cadherin, and down-regulated N-cadherin, Vimentin and MMP-9 (Fig. 2H; Supplement Fig. 2J). We further determined the effect of FTO on lung metastasis in vivo. CAL-62 cell lines with stably down-regulated FTO and control cells (NC) were established and intravenously injected into nude mice via the tail vein. The results showed that the luciferase activity of lung tumours derived from FTO-knockdown CAL-62 cells was significantly higher than that of control cells (Fig. 2I and K). We also found that FTO knockdown significantly increased the number of pulmonary nodules compared to that in control cells (Fig. 2 J and L). The panoramic section of HE staining suggested the similar results (Fig. 2J). These findings indicated that silencing of FTO promoted PTC and ATC cells migration and invasion, and over-expression of FTO reversed these effects.

### FTO regulates m6A modifications of CDH12 mRNA

**Fig. 3:**
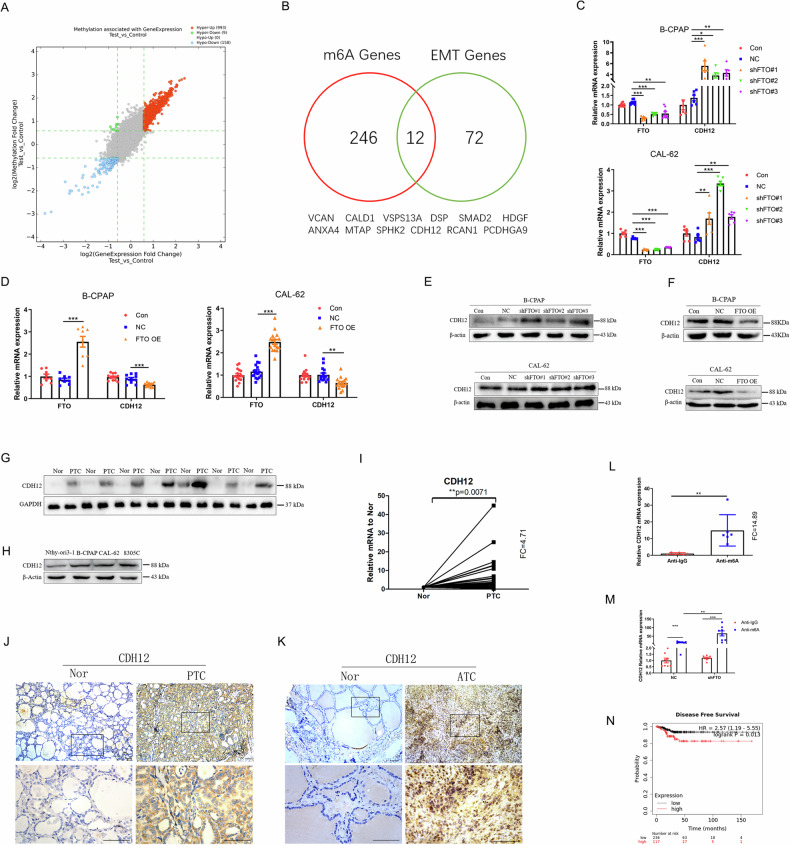
FTO regulates CDH12 mRNA m6A modifications in thyroid cancer. **A** M6A-mRNA epitranscriptomic microarray assay analysing the difference in m6A-related genes and mRNA levels between shFTO and control in CAL-62 cells (*n* = 3 repetitions). **B** Overlapping of 1.5-fold m6A expression changes in CAL-62 cells with FTO knockdown and EMT-related functional genes. **C** Influences of shFTO or (**D**) FTO OE on the mRNA level of CDH12 in B-CPAP and CAL-62 cells (*n* = 6 repetitions). **E–F** The influences of shFTO or FTO OE on the protein levels of CDH12 in B-CPAP and CAL-62 cells (*n* = 3 repetitions). **G** Protein level of CDH12 in PTC and Nor tissues (*n* = 7 paired). **H** The protein level of CDH12 in Nthy-ori 3-1, B-CPAP, CAL-62, and 8305 C cell lines (*n* = 3 repetitions). **I** The mRNA level of CDH12 in PTC tissues and Nor tissues (*n* = 40 paired). (**J**) Representative immunohistochemistry images of CDH12-positive cells in PTC and **K** ATC and Nor tissues (n = 5 paired). **L** MeRIP assay was performed to identify m6A antibody enrichment in CDH12 in CAL-62 cells (*n* = 4 repetitions). **M** Variation in m6A modification enrichment of CDH12 after FTO knockdown in CAL-62 cells (*n* = 4 repetitions). **N** Kaplan–Meier survival curves of disease-free survival based on CDH12. Bar=200 μm, ^*^p < 0.05, ^**^p < 0.01, ^***^p < 0.001.

FTO demethylates mRNAs to promote or suppress the invasion and metastasis of many cancers [28–30]. To identify the target genes that might be regulated by FTO in TC, m6A-mRNA epitranscriptomic microarray assay was used to identify FTO target genes, focusing on genes with high m6A levels and significant increases in mRNA expression. Concurrently, by combining eighty-four EMT-related genes with key functions [31], we identified twelve target genes of FTO (Fig. 3A and B). Then, according to the following conditions: (1) FTO could negatively regulate the mRNA level of the target gene in TC cells; (2) the expression of the target gene in thyroid cancer tissues was higher than that in Nor tissues; and (3) the m6A antibody could enrich the target RNA. Thus, CDH12 was identified as a key target gene regulated by FTO in thyroid cancer. We found that both the mRNA and protein levels of CDH12 were negatively regulated by FTO in TC cell lines (Fig. 3C, D, E and F; Supplement Fig. 3A and B). Our results showed that the expression of CDH12 increased significantly in both TC tissues and cell lines when compared to the control (Fig. 3G-K; Supplement Fig. 3C-E). Additionally, the MeRIP assay showed that the m6A antibody gathered large amounts of CDH12 RNA (Fig. 3L). When FTO was knocked down in CAL-62 cells, m6A abundance of CDH12 mRNA significantly increased (Fig. 3M). Furthermore, GEPIA2 data showed that patients with elevated CDH12 expression had a low disease-free-survival (DFS) (Fig. 3N). Therefore, CDH12 may be an important downstream target of FTO in thyroid carcinomas.

### FTO inhibits the invasion and metastasis of thyroid carcinoma by regulating CDH12 expression

**Fig. 4:**
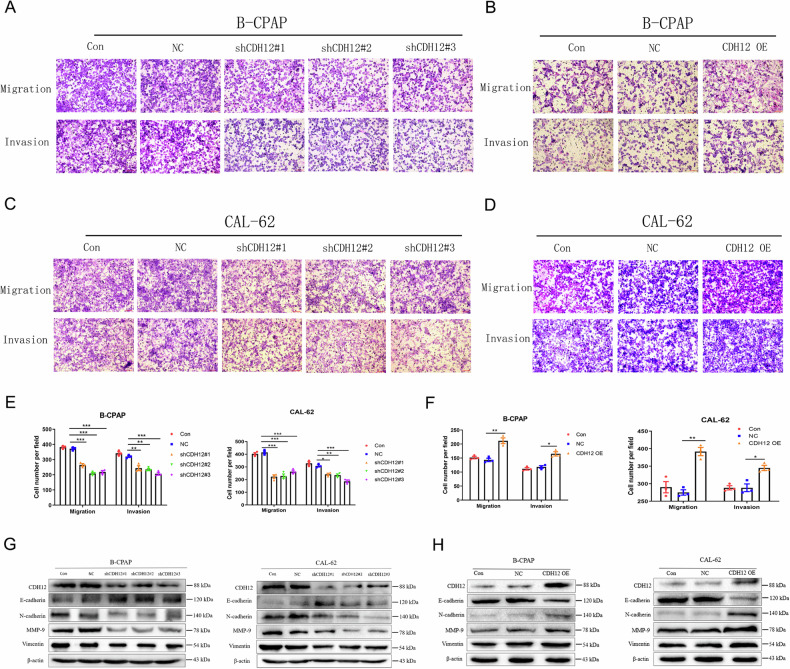
The role of CDH12 in thyroid carcinoma invasion and metastasis. **A, C** Transwell assay analysing the effects of CDH12 knockdown (shCDH12) or (**B, D**) CDH12 over-expression (CDH12 OE) on the invasive and migration capabilities in B-CPAP and CAL-62 cells and the quantitatively analyzed (**E–F**) (*n* = 3 repetitions). **G** Influences of shCDH12 or (**H**) CDH12 OE on the protein levels of E-cadherin, N-cadherin, MMP-9, Vimentin in B-CPAP and CAL-62 cells (*n* = 3 repetitions). Bar=200 μm, ^*^p < 0.05, ^**^p < 0.01, ^***^p < 0.001.

Several studies have confirmed that N-cadherin family proteins play an important role in the invasion and metastasis of various tumours [32, 33]. As a member of the N-cadherin family, CDH12 plays a significant role in carcinoma invasion and metastasis [25]. We found that CDH12 mRNA levels in PTC tissues with cervical lymph node metastasis were higher than those in tissues without cervical lymph node metastasis (Supplement Fig. 3F). Transwell and wound-healing assays showed that CDH12 knockdown significantly inhibited the invasion and migration capability of B-CPAP, CAL-62, and 8305 C cells (Fig. 4A, C and E; Supplement Fig. 4A, C, E and G), while CDH12 over-expression (CDH12 OE) reversed these effects (Fig. 4 B, D and F; Supplement Fig. 4B, D, F and H). Furthermore, we evaluated the effect of CDH12 on EMT in TC cells. These results suggested that CDH12 up-regulation promoted EMT activation in both B-CPAP and CAL-62 cells, whereas CDH12 knockdown partially inhibited the activation of EMT in TC cells (Fig. 4G, H; Supplement Fig. 4I and J). Therefore, our data suggest that CDH12 promotes the invasion and metastasis of PTC and ATC cells.

**Fig. 5:**
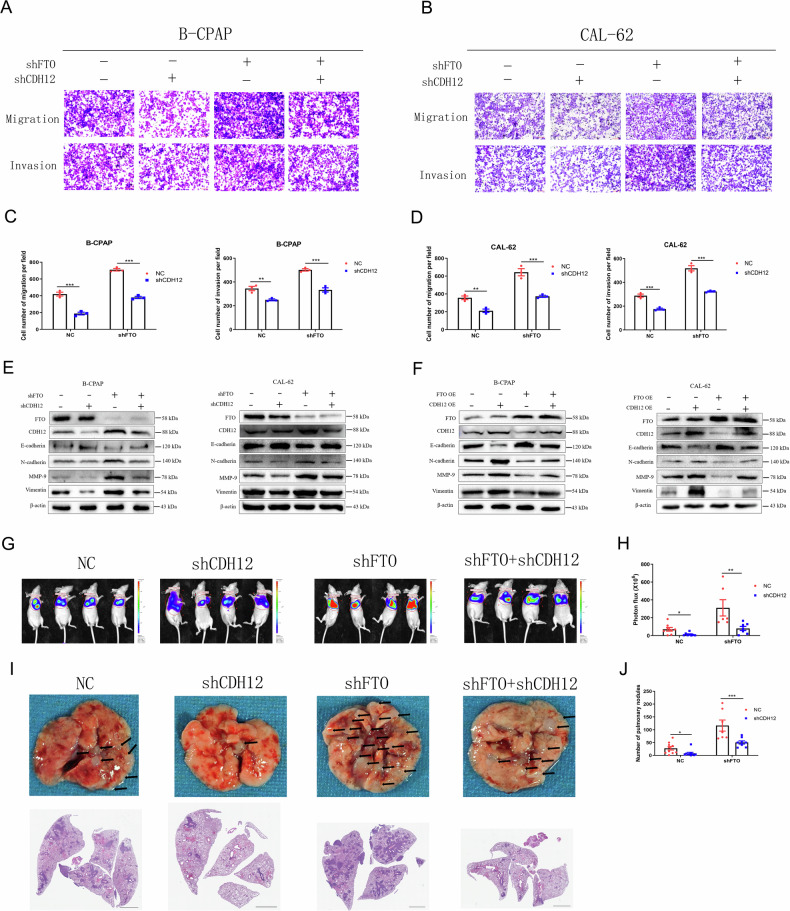
FTO inhibits the invasion and metastasis via CDH12 in thyroid carcinoma. (**A**) Effects of shFTO and shCDH12 on the invasive and migration capabilities of B-CPAP and (**B**) CAL-62 cells and the quantitatively analyzed (**C, D**) (*n* = 3 repetitions). **E** Protein levels of E-cadherin, N-cadherin, MMP-9 and Vimentin in the condition of shFTO and shCDH12 or (**F**) in the condition of FTO OE and CDH12 OE in B-CPAP and CAL-62 cells (*n* = 3 repetitions). **G** Luciferase activity of nude mice metastatic lung tumours were shown and (**H**) were quantitatively analyzed after CAL-62 cells infected with shFTO and shCDH12 virus injected (*n* = 8 mice). **I** Lung metastatic tumours of nude mice and the panoramic section HE staining results were shown and (**J**) were quantitatively analyzed after CAL-62 cells infected with shFTO and shCDH12 virus injected (*n* = 8 mice). Bar=200 μm for Transwell images and Bar=1 mm for HE images, ^*^p < 0.05, ^**^p < 0.01, ^***^p < 0.001.

Furthermore, transwell and wound-healing assays revealed that CDH12 knockdown reversed the effects of FTO silencing on TC cell migration and invasion (Fig. 5A-D; Supplement Fig. 5A-F). Next, western blot assay revealed that CDH12 knockdown reversed the effects of FTO silencing on EMT activation, whereas CDH12 up-regulation produced the opposite effect of FTO over-expression on EMT activation in both B-CPAP and CAL-62 cells (Fig. 5E and F; Supplement Fig. 5G-J). Additionally, we investigated the role of CDH12 down-regulation and FTO silencing in CAL-62 cell lung metastasis in vivo. CDH12 down-regulation significantly reversed the effects of FTO knockdown on the luciferase activity of lung tumours and the number of pulmonary nodules induced by CAL-62 cells (Fig. 5G-J). These data fully demonstrate that FTO inhibits invasion, migration, and EMT in thyroid cancer via regulating the expression of CDH12.

### IGF2BP2 regulates CDH12 mRNA stability through m6A-dependent manner in thyroid cancer

**Fig. 6:**
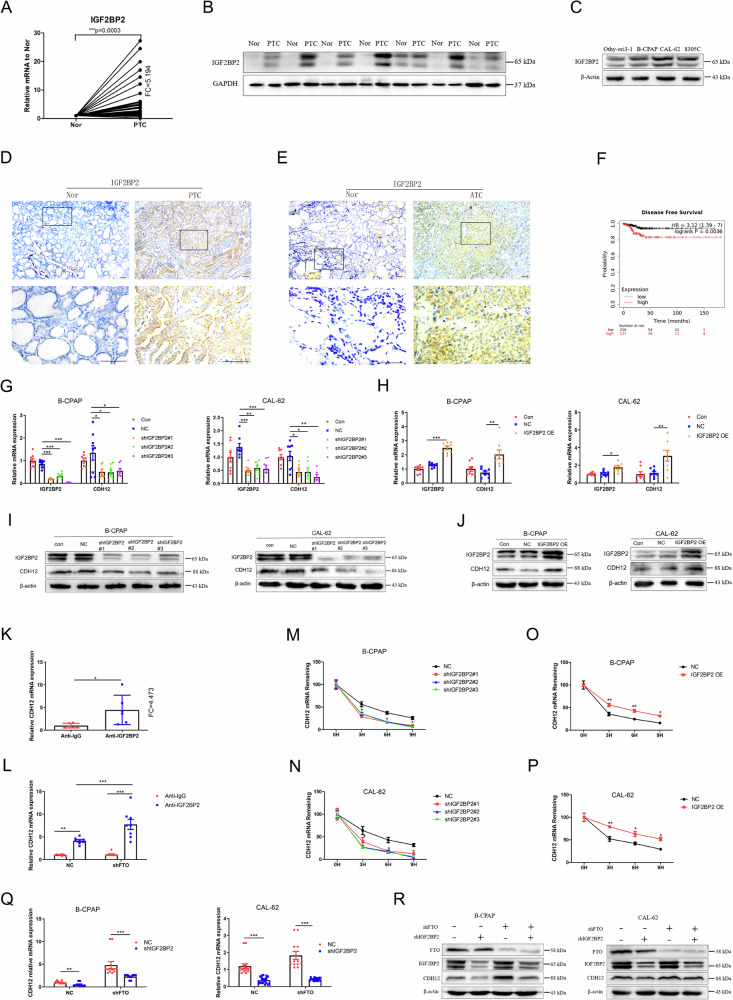
IGF2BP2 regulates CDH12 mRNA stability through m6A-dependent manner in thyroid cancer. **A** The mRNA expression level of IGF2BP2 in PTC tissues and the Nor tissues (*n* = 40 paired). **B** Protein level of IGF2BP2 in PTC and Nor tissues (*n* = 7 paired). **C** Protein level of IGF2BP2 in Nthy-ori 3-1, B-CPAP, CAL-62, and 8305c cell lines (*n* = 3 repetitions). **D** Representative immunohistochemistry images of IGF2BP2-positive cells in PTC and (**E**) ATC and Nor tissues (*n* = 5 paired). **F** Kaplan–Meier survival curves of disease-free survival based on IGF2BP2. **G** Influences of IGF2BP2 knockdown (shIGF2BP2) or (**H**) IGF2BP2 over-expression (IGF2BP2 OE) on the mRNA level of CDH12 in B-CPAP and CAL-62 cell lines (*n* = 6 repetitions). **I** The influences of shIGF2BP2 or (**J**) IGF2BP2 OE on the protein level of CDH12 in B-CPAP and CAL-62 cells (*n* = 3 repetitions). **K** RIP-qPCR showed the enrichment of CDH12 binding to IGF2BP2 in CAL-62 cells (*n* = 4 repetitions). **L** The mRNA level of CDH12 binding to IGF2BP2 after FTO knockdown in CAL-62 cells (n = 4 repetitions). **M, N** CDH12 mRNA half-life (t 1/2) after IGF2BP2 knockdown in B-CPAP and CAL-62 cells. **O, P** CDH12 mRNA half-life (t 1/2) after IGF2BP2 overexpression in B-CPAP and CAL-62 cells (*n* = 6 repetitions). **Q** Influences of shFTO and shIGF2BP2 on the mRNA level of CDH12 in B-CPAP and CAL-62 cells (*n* = 6 repetitions). **R** Influences of shFTO and shIGF2BP2 on the protein level of CDH12 in B-CPAP and CAL-62 cells (*n* = 3 repetitions). Bar=200 μm, ^*^p < 0.05, ^**^p < 0.01, ^***^p < 0.001.

By recognising m6A modifications, m6A reader proteins play a key role in regulating mRNA fate [34]. To identify the key reader proteins of m6A that participate in TC, we systematically analysed the mRNA levels of m6A reader proteins in TC and Nor tissues (Fig. 6A, Supplement Fig. 6A-F). The results showed that the mRNA levels of IGF2BP2 were significantly increased in PTC tissues. We further demonstrated that the protein level of IGF2BP2 was considerably enhanced in both TC tissues and cell lines (Fig. 6B-E; Supplement Fig. 6G-I). Moreover, the protein and mRNA expression levels of CDH12 were markedly reduced after IGF2BP2 was silenced (Fig. 6G and I; Supplement Fig. 6J), and this phenomenon was reversed by the over-expression of IGF2BP2 (Fig. 6 H and J; Supplement Fig. 6K). RIP-qPCR was used to investigate whether IGF2BP2 directly recognises m6A modifications in CDH12 mRNA. As expected, CDH12 mRNA was gathered using the IGF2BP2 antibody, and FTO knockdown enhanced IGF2BP2 abundance in CDH12 mRNA (Fig. 6 K and L). These findings indicate that IGF2BP2 directly binds to CDH12 mRNA. After silencing IGF2BP2 expression in B-CPAP and CAL-62 cells, the mRNA stability of CDH12 was remarkably reduced (Fig. 6M and N), whereas over-expression of IGF2BP2 enhanced the mRNA stability of CDH12 (Fig. 6 O and P), indicating that IGF2BP2 is involved in maintaining the stability of CDH12 mRNA. IGF2BP2 knockdown partially rescued the mRNA and protein expression of CDH12 caused by FTO down-regulation in B-CPAP and CAL-62 cells (Fig. 6 Q and R; Supplement Fig. 6L). These results indicated that IGF2BP2 recognised m6A modifications in CDH12 and induced mRNA degradation.

### IGF2BP2 promotes the invasion and metastasis of thyroid carcinoma via CDH12

**Fig. 7:**
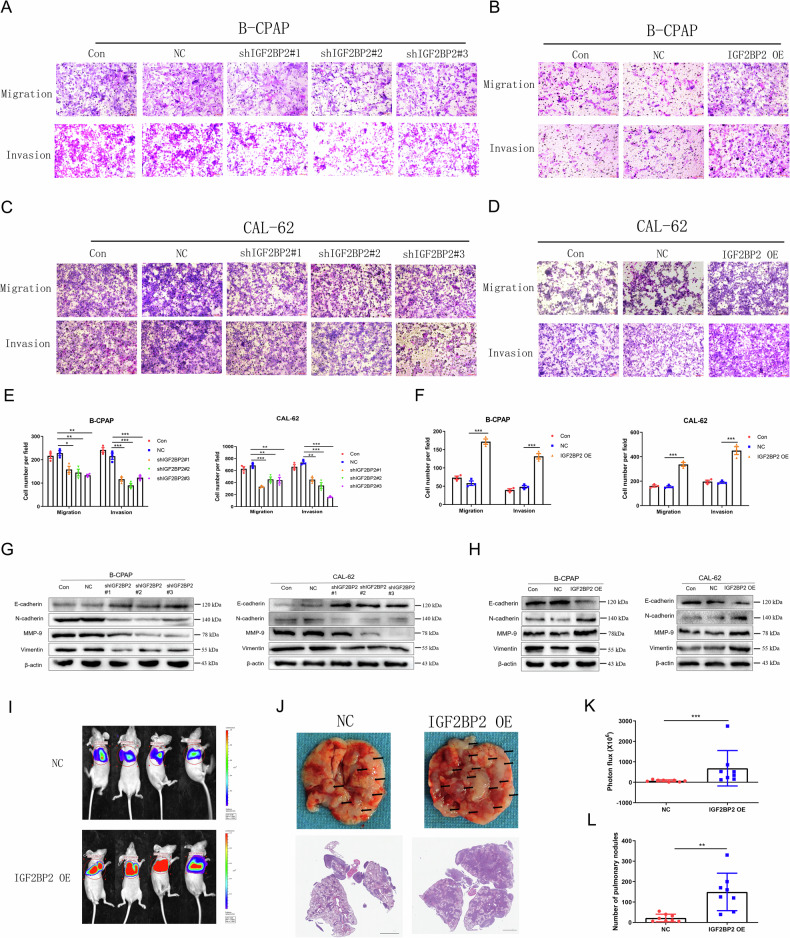
The role of IGF2BP2 in thyroid carcinoma invasion and metastasis. (**A, C**) Effects of shIGF2BP2 or (**B, D**) IGF2BP2 OE on the invasive and migration capabilities in B-CPAP and CAL-62 cells and the quantitatively analyzed (**E–F)** (*n* = 3 repetitions). **G** Influences of shIGF2BP2 or (**H**) IGF2BP2 OE on the protein levels of E-cadherin, N-cadherin, MMP-9 and Vimentin in B-CPAP and CAL-62 cells (*n* = 3 repetitions). **I** Luciferase activity of nude mice metastatic lung tumours were shown and (**K**) were quantitatively analyzed after CAL-62 cells infected with shIGF2BP2 and shRNA-NC virus injected (*n* = 8 mice). **J** Lung metastatic tumours of nude mice and the panoramic section HE staining results were shown and (**K–L**) were quantitatively analyzed after CAL-62 cells infected with shIGF2BP2 and shRNA-NC virus injected (*n* = 8 mice). Bar=200 μm for Transwell images and Bar=1 mm for HE images, ^*^p < 0.05, ^**^p < 0.01, ^***^p < 0.001.

M6A readers play important roles in cancer biology [35–37]. In the present study, we found that IGF2BP2 mRNA levels in PTC tissues with cervical lymph node metastasis (LM + ) were higher than the those in tissues without cervical lymph node metastasis (LM-) (Supplement Fig. 6M), indicating that increased IGF2BP2 expression may participate in PTC metastasis. We found that the silencing of IGF2BP2 was associated with reduced TC migration and invasion (Fig. 7A, C and E; Supplement Fig. 7A, C, E and G), while the over-expression of IGF2BP2 promoted the migration and invasion capacity of TC (Fig. 7B, D and F; Supplement Fig. 7B, D, F and H). Western blotting demonstrated that silencing of IGF2BP2 inhibited the activation of EMT, whereas over-expression of IGF2BP2 produced the opposite functions (Fig. 7G and H; Supplement Fig. 7I and J). In vivo, we found that IGF2BP2 up-regulation significantly increased the luciferase activity of lung tumours and the number of pulmonary nodules induced by CAL-62 cells (Fig. 7I-L). Therefore, IGF2BP2 plays an oncogenic role in thyroid cancer invasion and metastasis.

**Fig. 8:**
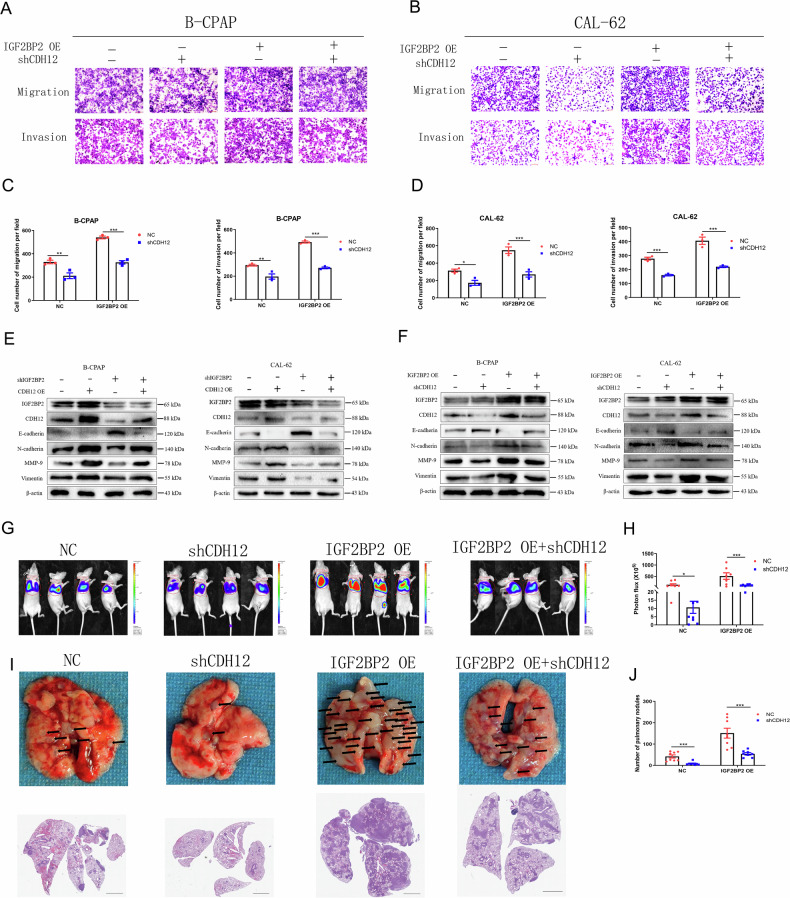
IGF2BP2 inhibits the invasion and metastasis via CDH12 in thyroid carcinoma. (**A**) Effects of IGF2BP2 OE and CDH12 knockdown on the invasive and migration capabilities of B-CPAP and (**B**) CAL-62 cells with transwell assay and the quantitatively analyzed (**C–D**) (*n* = 3 repetitions). **E** Protein levels of E-cadherin, N-cadherin, MMP-9 and Vimentin in the condition of shIGF2BP2 and CDH12 OE or (**F**) in the condition of IGF2BP2 OE and shCDH12 in B-CPAP and CAL-62 cells (*n* = 3 repetitions). **G** Luciferase activity of nude mice metastatic lung tumours were shown and (**H**) were quantitatively analyzed after CAL-62 cells infected with IGF2BP2 OE and shCDH12 virus injected (*n* = 8 mice). **I** Lung metastatic tumours of nude mice and the panoramic section HE staining results were shown and (**J**) were quantitatively analyzed after CAL-62 cells infected with IGF2BP2 OE and shCDH12 virus injected (*n* = 8 mice). Bar=200 μm for Transwell images and Bar=1 mm for HE images, ^*^p < 0.05, ^**^p < 0.01, ^***^p < 0.001.

Although IGF2BP2 over-expression promoted the invasion and metastasis of thyroid cancer, CDH12 knockdown reversed the effects of IGF2BP2 over-expression on TC cell migration and invasion (Fig. 8A-D; Supplement Fig. 8A-F). Western blotting also revealed that CDH12 knockdown reversed the effects of IGF2BP2 over-expression on the activation of EMT, while CDH12 over-expression reversed the effects induced by silencing IGF2BP2 on EMT activation (Fig. 8 E and F; Supplement Fig. 8G-J). Furthermore, we explored the role of CDH12 down-regulation and IGF2BP2 over-expression on CAL-62 cells lung metastasis in vivo. The results showed that CDH12 down-regulation reduced the lung metastasis caused by IGF2BP2 over-expression (Fig. 8G-J). This finding indicates that IGF2BP2 promotes the invasion and migration of PTC and ATC cells via CDH12.

## Discussion

Studies have shown that thyroid cancer patients with lymph node metastasis, invasion of extranodal tissues, or distant metastasis can have worse prognosis [38]. m6A regulates the progression of EMT [20], which is an important biological process during tumour invasion and migration [39]. In the present study, we first identified that (1) FTO can negatively regulate the EMT process and the invasion and migration ability of PTC and ATC; (2) FTO can negatively regulate CDH12 expression in an m6A dependent manner and then mediate EMT to promote the invasion and metastasis of PTC and ATC; and (3) IGF2BP2 can regulate the stability of CDH12 mRNA in an m6A dependent manner and then mediate EMT, thereby promoting the invasion and metastasis of PTC and ATC. Thus, FTO/IGF2BP2-mediated CDH12 m6A modification may promote the invasion and migration of PTC and ATC cells.

Multiple histological types of thyroid cancers have been described, including PTC, the most common, and ATC, the most lethal. However, over 95% of patients with PTC are curable and achieve a 10-year survival [40]. Some PTCs have an aggressive phenotype associated with malignant clinical outcomes due to the comprehensive invasion of metastasis [38]. While ATC is an undifferentiated type that exhibits high proliferative potential and resistance to current therapies [41], it remains a deadly cancer with an overall survival rate of 3-5 months after initial diagnosis [40]. Histologically, PTC progresses to ATC by dedifferentiation, a biological process in pervasive cancers that induces the transition of cancer from a highly to a poorly differentiated status [42]. In addition, the derivation of ATC from PTC is also suggested by other indirect evidence, including the frequent histopathological coexistence of PTC components in ATC lesions and a history of PTC in most patients with ATC [42]. A current study demonstrated that EMT represents the main program activated in cancer cells to ensure the acquisition of an aggressive phenotype [43]. Therefore, it is of clinical significance to identify common upstream factors that regulate the EMT pathway and the progression of invasion and migration in both PTC and ATC.

m6A RNA modification is prevalent and functionally modulates the eukaryotic transcriptome to affect mRNA export, splicing, translation, localization, and stability [44]. Currently, an increasing number of studies are focusing on the potential links between m6A and cancers, especially their invasive and metastatic characteristics [45]. For example, the inhibition of METTL3-mediated m6A modification can promote the migration and invasion of endometrial stromal cells in endometriosis [45]; and m6A modification is critical for EMT and metastasis of gastric cancer [19]. In contrast, a recent study demonstrated that METTL3-mediated m6A modification inhibited PTC progress [46]. However, there have been no systematic studies on the role of m6A in the invasion and metastasis of thyroid cancers, particularly ATC. Importantly, our study demonstrated that the level of m6A was significantly increased in both PTC and ATC tissues, suggesting that m6A may play a key role in the progression of TC. Consistent with the m6A level, we demonstrated that the level of the FTO, which is an ‘eraser’ of the m6A modification regulator, was significantly decreased both in PTC and ATC tissues and cell lines. These results were consistent with a previous study on PTC, which showed that FTO was down-regulated and that the overall level of m6A was higher in PTC tissues [47]. Research has found that FTO regulates PTC proliferation via SLC7A11 in a ferroptosis-dependent manner [48]. Our results verified that FTO expression is lower in thyroid cancer tissues with lymph node metastasis. Furthermore, we demonstrated that FTO knockdown enhanced the invasion and migration capacity of B-CPAP, CAL-62, and 8305 C cells through the EMT pathway, whereas FTO over-expression produced the opposite effects. In vivo, FTO knockdown enhanced the lung metastatic ability of CAL-62 cells. Thus, these data demonstrate that FTO is significantly down-regulated in PTC and ATC and may play a tumour suppressive role in the regulation of TC invasion and migration through the EMT pathway.

To further explore the mechanism of FTO in the regulation of EMT progression in TC, an m6A-mRNA epitranscriptomic microarray in CAL-62 with FTO knockdown was performed and demonstrated that CDH12 was negatively regulated by FTO and modified by FTO-mediated m6A methylation, as validated by MeRIP-qPCR. Additionally, we found that the expression of CDH12 was increased in TC tissues and cell lines. A previous study reported that CDH12 promotes colorectal cancer cell metastasis by promoting EMT [49]; however, its role in TC has not been elucidated. To further verify the function of CDH12 in TC, we found a suppressive role of down-regulated CDH12 in TC cell migration and invasion through the EMT pathway. Consistently, CDH12 promotes TC cell migration and invasion. These data indicate the important role of CDH12 in TC cell metastasis which agrees with the findings in salivary adenoid cystic carcinoma [50] and colorectal cancer [49]. Critically, the role of FTO in mediating the expression of EMT-related factors and invasion and migration functions was abolished by CDH12 intervention. Moreover, the results of the lung metastasis model in nude mice showed that the knockdown of CDH12 significantly reversed the lung metastatic ability of CAL-62 cells induced by FTO knockdown. These results suggested that FTO negatively regulates CDH12 expression in an m6A dependent manner and mediates EMT to promote the invasion and metastasis of thyroid cancer.

Studies have reported that m6A-modified mRNA transcripts are mainly mediated by m6A readers such as the YTH domain family members or IGF2BPs [51–53]. The mRNA transcripts with m6A modifications are targeted by different m6A readers; therefore, they may have different outcomes. For example, YTHDC2, YTHDF2 and YTHDF3 promote the decay of their target mRNA transcripts [54, 55], whereas IGF2BP1-3 could promote the stability of their target mRNA [36]. The current study showed that IGF2BP2 expression was significantly increased in both PTC and ATC tissues and cell lines. Further analysis revealed that IGF2BP2 knockdown significantly reduce the expression of CDH12 in TC. Moreover, an RNA stability assay showed that the half-life of CDH12 mRNA was significantly reduced after IGF2BP2 knockdown, and vice versa. The RIP assay data indicated that IGF2BP2 bound to CDH12 mRNA. These findings indicate that IGF2BP2 is a key protein that regulates the stability of CDH12 mRNA in an m6A-dependent manner. Accumulating evidence has revealed the oncogenic role of IGF2BP2 in tumorigenesis and metastasis [56–58]. Experiments with IGF2BP2-deficient mice indicated that IGF2BP2 is a tumour-promoting factor that facilitates cancer progression and metastasis [59]. Moreover, researches have been demonstrated that IGF2BP2 promoted the thyroid cancer progression via RUNX2 [60] or long non-coding RNA HAGLR [61]. Our present study also demonstrated that over-expression of IGF2BP2 could enhance the capacity for invasion and migration of PTC and ATC cells and promote EMT progression, whereas IGF2BP2 over-expression could produce the opposite effects. Furthermore, the in vivo experiments showed that knockdown of CDH12 significantly reversed the lung metastatic ability of CAL-62 cells induced by the over-expression of IGF2BP2. These results suggest that IGF2BP2 regulates the stability of CDH12 mRNA in an m6A dependent manner and mediates EMT, thereby promoting the invasion and metastasis of PTC and ATC.

## Abbreviations

FTO: Fat mass and obesity associated gene; CDH12: Cadherin 12; IGF2BP2: Insulin-like growth factor 2 mRNA-binding protein 2; EMT: Epithelial mesenchymal transition; TC: Thyroid cancer; m6A: N6 methyladenosine; PTC: Papillary thyroid cancer; ATC: Anaplastic thyroid cancer; METTL3/14: Methyltransferase-like 3/14; WTAP: Wilm’s tuner 1-associated protein; KIAA1429:Vir-like m6A methyltransferase-associated protein; ALKBH5:Alk B homolog 5; YTH:YT521-B homology; eIF3: Eukaryotic initiation factor 3; HRP: Horseradish peroxidase; MMP-9: Matrix metalloproteinase 9.

## Conclusions

The current study indicates that low levels of FTO epigenetically promote the expression of CDH12 through IGF2BP2-mediated m6A modification, and that CDH12 promotes the invasion and metastasis of PTC and ATC through the EMT pathway. Thus, our results reveal a novel molecular mechanism of PTC and ATC progression regulated by m6A modification and provide valuable insights for the development of efficient therapeutic strategies against TC.

## Supplementary information

Supplementary materials

Supplement2-original Western blot bands

## Data Availability

The datasets supporting the conclusions of this article are included within the article and its supplementary files.
