# Supplementary material for: FTO/IGF2BP2-mediated N6 methyladenosine modification in invasion and metastasis of thyroid carcinoma via CDH12
Source: Cell Death Dis. 2024 Oct 8;15(10):733. doi: 10.1038/s41419-024-07097-4 (PMC11461506; doi:10.1038/s41419-024-07097-4)
Supplement: Supplementary file 1 — Supplementary materials [file 41419_2024_7097_MOESM1_ESM.doc]

FTO/IGF2BP2-mediated N6 methyladenosine modification in invasion and metastasis of thyroid carcinoma via CDH12

Zuyao Chen^1.2*^, Xiaolin Zhong^1,4*^, Min Xia^1^, Chang Liu^5^, Weiqiang Tang^1^, Gaohua Liu^1^, Yan Yi^1,3^, Yinping Guo^1,3^, Qingshan Jiang^2^, Xuyu Zu^1,3,#^, Jing Zhong^1,3,#^

1. Clinical Medical Research Center, The First Affiliated Hospital, Hengyang Medical School, University of South China, 421001, Hengyang, Hunan, China
2. Department of Otorhinolaryngology, The First Affiliated Hospital, Hengyang Medical School, University of South China, 421001, Hengyang, Hunan, China
3. Institute of Cancer Research, The First Affiliated Hospital, Hengyang Medical School, University of South China, 421001, Hengyang, Hunan, China
4. Department of Endocrinology and Metabolism, The First Affiliated Hospital, Hengyang Medical School, University of South China, 421001, Hengyang, Hunan, China
5. Department of Endocrinology and Metabolism, The First People's Hospital of Chenzhou, The First School of Clinical Medicine, University of Southern Medical, 510515, China.

^*^These authors contributed equally to this work.

^#^Corresponding authors:

Jing Zhong (Co-corresponding author)

Institute of Cancer Research, The First Affiliated Hospital, Hengyang Medical School, University of South China, 421001, Hengyang, Hunan, China

zhongjing2002@usc.edu.cn

Xuyu Zu (Co-corresponding author)

Institute of Cancer Research, The First Affiliated Hospital, Hengyang Medical School, University of South China, 421001, Hengyang, Hunan, China

Zuxuyu0108@163.com

**Materials and Methods**

**Table 1. Information for the primers used in this study**

| Genes | Primers | Sequence 5’-3’ |
| --- | --- | --- |
| METTL3 | *forwards*  *reverse* | CTTCAGCAGTTCCTGAATTAGC  ATGTTAAGGCCAGATCAGAGAG |
| METTL14 | *forwards*  *reverse* | ACCAAAATCGCCTCCTCCCAAATC  AGCCACCTCTTTCTCCTCGGAAG |
| KIAA1429 | *forwards*  *reverse* | GGAATGGACACGTTTATTCGAG  GATAGAGCACAGGAGCATATGT |
| WTAP | *forwards*  *reverse* | CTGACAAACGGACCAAGTAATG  AAAGTCATCTTCGGTTGTGTTG |
| FTO | *forwards*  *reverse* | GTTCACAACCTCGGTTTAGTTC  CATCATCATTGTCCACATCGTC |
| ALKBH5 | *forwards*  *reverse* | TCCTTTCCCTTCCCTTCTCCACTG  TGAAGCGGAGGAGGCACCAG |
| VCAN | *forwards*  *reverse* | ACTGAAACTTCCTACGTATGCA  CTCACAAAGTGCACCAACATAA |
| CALD1 | *forwards*  *reverse* | AGAAAAGCAGTGGTGTCAAATC  CAATTGCACTGGTATACTGCTC |
| VSP13A | *forwards*  *reverse* | AAAATGGCCATTGTTGAGTCAG  CAGCTTCTGTAAATGCCTTGAC |
| DSP | *forwards*  *reverse* | AACTTGAAGAAGTCGTTGTTGG  GTTTATCTATCCTTTGCCAGCG |
| SMAD2 | *forwards*  *reverse* | CTCTTCTGGCTCAGTCTGTTAA  AAGGAGTACTTGTTACCGTCTG |
| HDGF | *forwards*  *reverse* | GCT TCCGGCTATCAGTCCTC  CTGCCTCCTTCTCCTCTCCT |
| ANXA4 | *forwards*  *reverse* | CTTGAAGATGACATTCGCTCTG  TAATTTCCTTCATCCCTCCCAC |
| MTAP | *forwards*  *reverse* | GACCTCAGTCCTTCTATGATGG  TGGAACTGTGGTCATGTTGATA |
| SPHK2 | *forwards*  *reverse* | TACTTCTGCATCTACACCTACC  CACGGTTCTCTTCGTAGGTG |
| CDH12 | *forwards*  *reverse* | TTGAGGAGAACAAAATTCGCAG  ATATGTGGCCAGTGAATCGTAT |
| RCAN1 | *forwards*  *reverse* | CGACCCCAGTCATAAACTATGA  CCATTTCCTCTTCTTCCTCCTT |
| PCDHGA9 | *forwards*  *reverse* | TCTTCCTCACCTTCGTTATCAC  AATAGGTCTGTAGGAAAGCTCG |
| YTHDC1 | *forwards*  *reverse* | AGTGACTCTGGTTCTGAATCTG  CTGGTTTGATCTTTTCGGACAG |
| YTHDC2 | *forwards*  *reverse* | ACCACCATGGAGAAGGCTGG  CTCAGTGTAGCCCAGGATGC |
| YTHDF1 | *forwards*  *reverse* | ACCACCATGGAGAAGGCTGG  CTCAGTGTAGCCCAGGATGC |
| YTHDF2 | *forwards*  *reverse* | ACCACCATGGAGAAGGCTGG  CTCAGTGTAGCCCAGGATGC |
| YTHDF3 | *forwards*  *reverse* | ACCACCATGGAGAAGGCTGG  CTCAGTGTAGCCCAGGATGC |
| IGF2BP1 | *forwards*  *reverse* | ACCACCATGGAGAAGGCTGG  CTCAGTGTAGCCCAGGATGC |
| IGF2BP2 | *forwards*  *reverse* | ACCACCATGGAGAAGGCTGG  CTCAGTGTAGCCCAGGATGC |
| IGF2BP3 | *forwards*  *reverse* | ACCACCATGGAGAAGGCTGG  CTCAGTGTAGCCCAGGATGC |
| eIF3 | *forwards*  *reverse* | ACCACCATGGAGAAGGCTGG  CTCAGTGTAGCCCAGGATGC |
| GAPDH | *forwards*  *reverse* | GATTCCACCCATGGCAAATTC  CTGGAAGATGGTGATGGGATT |

**Table 2.** Information for the primary antibodies used in this study

| Antibody | Company | Lot number | Molecular weight (KDa) | Dilution ratio |
| --- | --- | --- | --- | --- |
| m6A | Abclonal | A19841 |  | 1:500 |
| FTO | Proteinteck | 27226-1-AP | 58 kDa | 1:1000 |
| CDH12 | Abcam | Ab181860 | 88 kDa | 1:400 |
| IGF2BP2 | Proteinteck | 11601-1-AP | 65 kDa | 1:1000 |
| E-cadherin | BD biosciences | 610182 | 120 kDa | 1:200 |
| N-cadherin | Abclonal | A19083 | 140kDa | 1:200 |
| MMP-9 | Proteinteck | 10375-2-AP | 78 kDa | 1:1000 |
| Vimentin | Proteinteck | 10366-1-AP | 54 kDa | 1:1500 |
| β-actin | Origene | TA811000 | 43 kDa | 1:1500 |

**Supplementary figures**

**
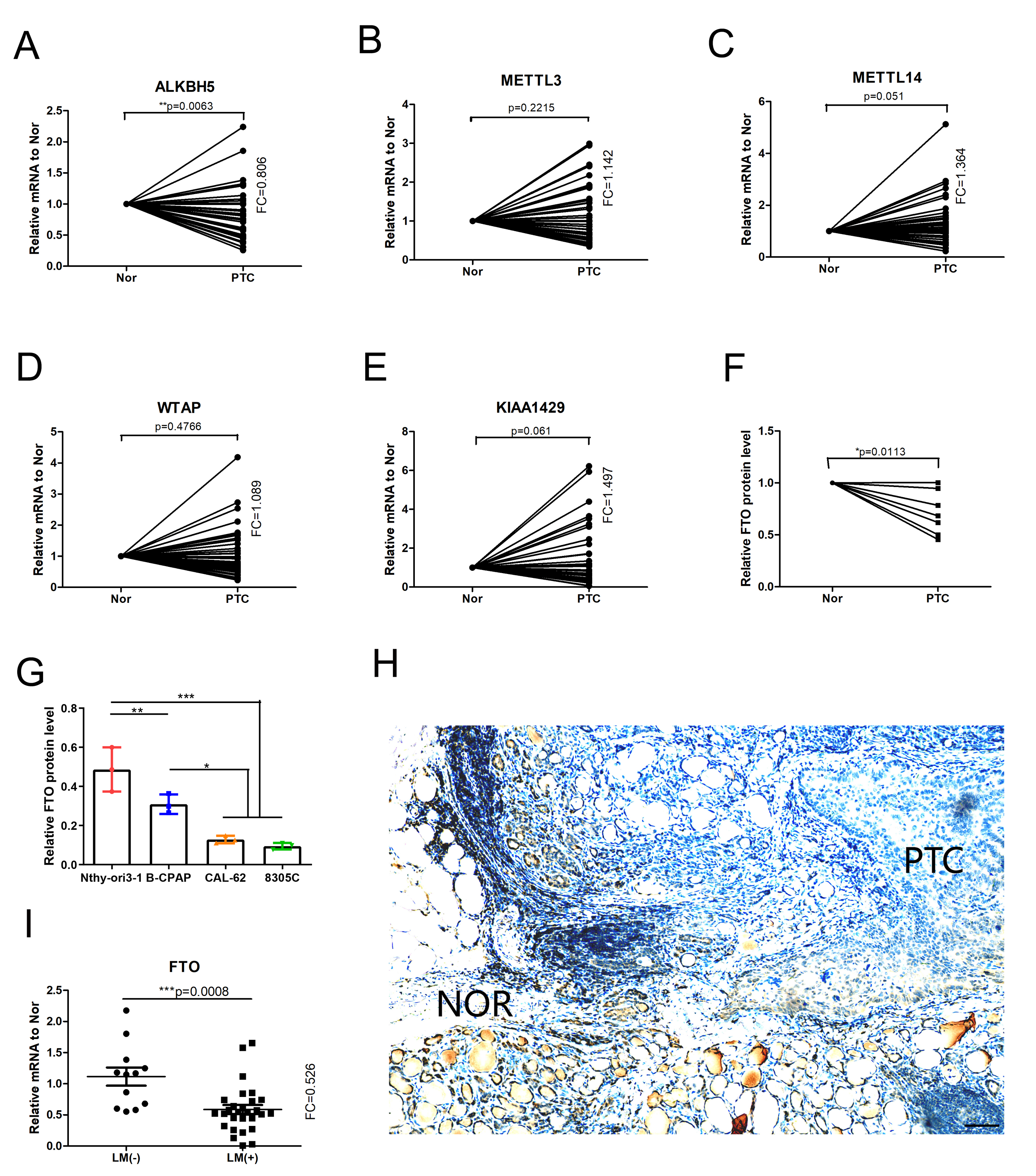
**

**Supplement Figure. 1 The expression of FTO in thyroid carcinoma.** (A-E) The mRNA levels of m6A modification enzymes in papillary thyroid carcinoma (PTC) tissues and the adjacent normal tissues (Nor). (F) Semiquantitative analysis of the FTO protein levels of PTC and Nor tissues relative to GAPDH. (G) Semiquantitative analysis of the FTO protein levels of Nthy-ori 3-1, B-CPAP , CAL-62, and 8305C relative to β-Actin. (H) Representative immunohistochemistry images of FTO negative cells in PTC and FTO positive cells in adjacent normal tissues from the same section. (I) The mRNA level of FTO in PTC tissues with (LM+) or without (LM-) cervical lymph node metastasis. Bar=200 μm. **p*<0.05, ***p*<0.01, ****p*<0.001.

**
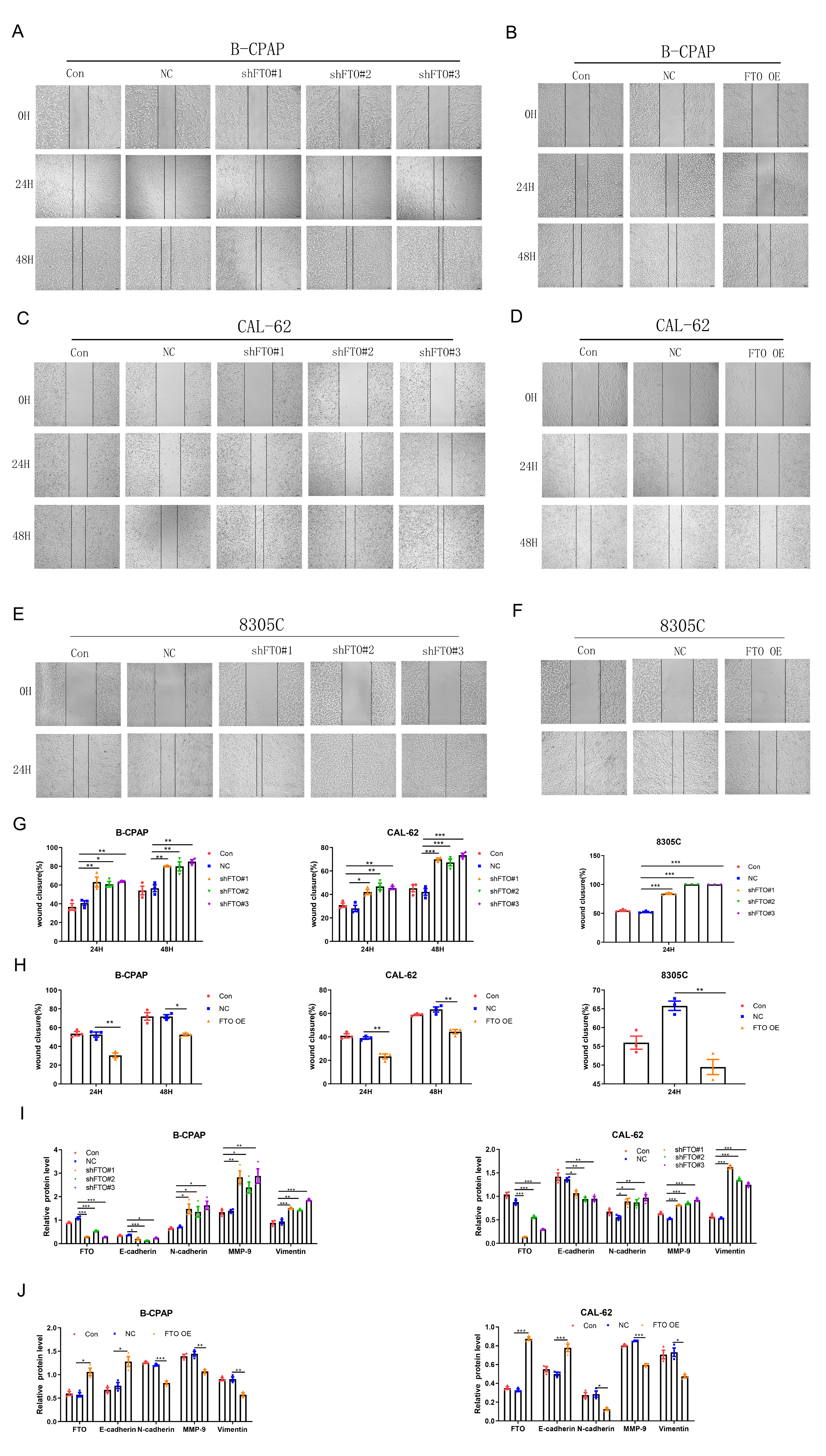
**

**Supplement Figure. 2 The role of FTO on the migration in thyroid carcinoma.** (A) Effects of FTO knockdown (shFTO) or (B) over-expression (FTO OE) on the migration capability of B-CPAP with wound-healing assay were recorded and quantitatively analyzed (G-H). (C) Effects of shFTO or (D) FTO OE on the migration capability of CAL-62 with wound-healing assay were recorded and (G-H) quantitatively analyzed. (E) Effects of shFTO or (F) FTO OE on the migration capability of 8305C with wound-healing assay were recorded and (G-H) quantitatively analyzed. (I-J) Semiquantitative analysis of shFTO or FTO OE on the protein levels of FTO, E-cadherin, N-cadherin, MMP-9, and Vimentin in B-CPAP and CAL-62 cells relative to β-Actin. Bar=200 μm, **p*<0.05, ***p*<0.01, ****p*<0.001.

**
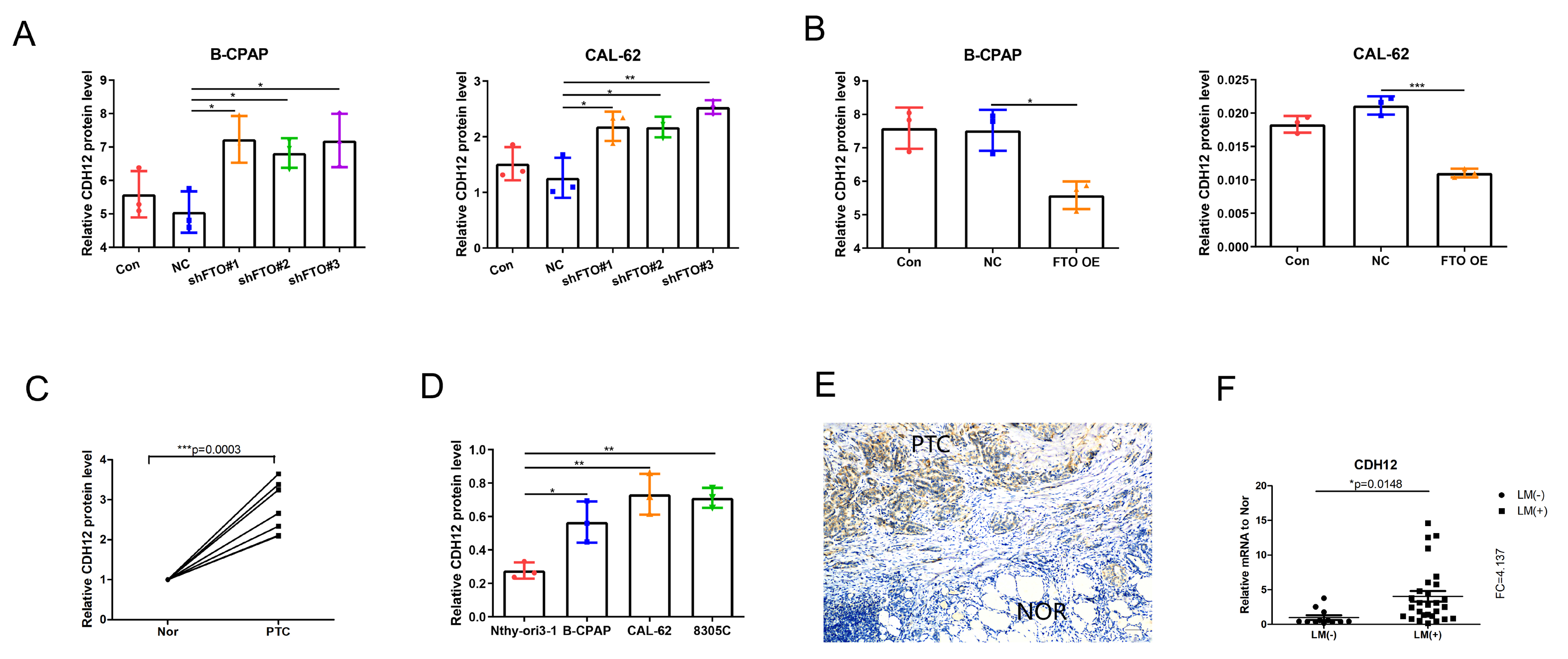
**

**Supplement Figure. 3 The expression of CDH12 in thyroid cancer.** (A-B) Semiquantitative analysis of shFTO or FTO OE on the protein levels of CDH12 in B-CPAP and CAL-62 cells relative to β-Actin. (C) Semiquantitative analysis of the CDH12 protein levels of PTC and Nor tissues relative to GAPDH. (D) Semiquantitative analysis of the CDH12 protein levels of Nthy-ori 3-1, B-CPAP , CAL-62, and 8305C relative to β-Actin. (E) Representative immunohistochemistry images of CDH12 positive cells in PTC and CDH12 negative cells in adjacent normal tissues from the same section. (F) The mRNA level of CDH12 in PTC tissues with (LM+) or without (LM-) cervical lymph node metastasis. Bar=200 μm. **p*<0.05, ***p*<0.01, ****p*<0.001.

**
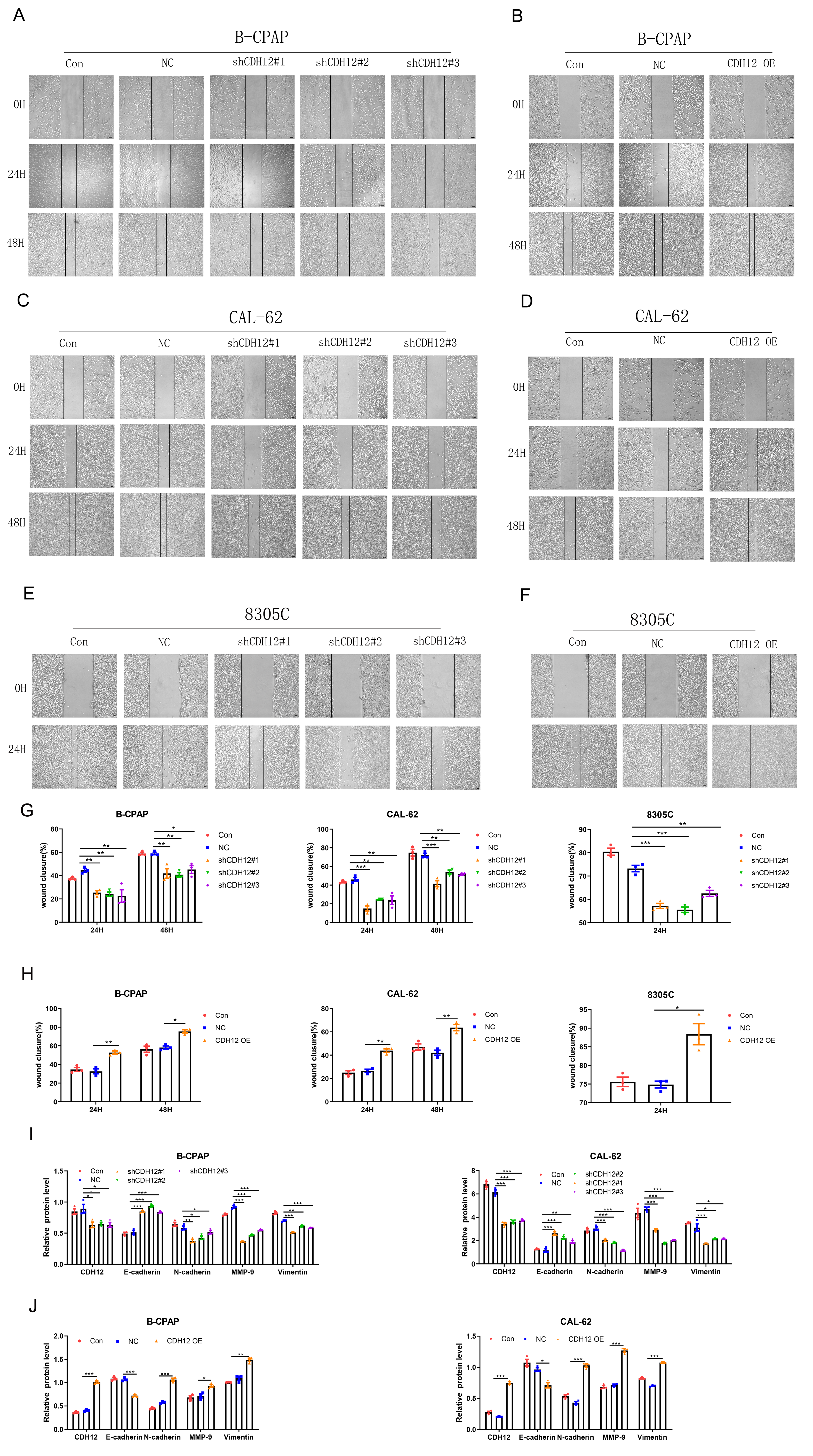
**

**Supplement Figure. 4 The role of CDH12 on the migration of thyroid carcinoma.** (A)Effects of CDH12 knockdown (shCDH12) or (B) over-expression (CDH12 OE) on the migration capability of B-CPAP with wound-healing assay were recorded and quantitatively analyzed (G-H). (C) Effects of shCDH12 or (D) CDH12 OE on the migration capability of CAL-62 with wound-healing assay were recorded and quantitatively analyzed (G-H). (E) Effects of shCDH12 or (F) CDH12 OE on the migration capability of 8305C with wound-healing assay were recorded and quantitatively analyzed (G-H). (I) Semiquantitative analysis of shCDH12 or (J) CDH12 OE on the protein levels of CDH12, E-cadherin, N-cadherin, MMP-9, and Vimentin in B-CPAP and CAL-62 cells relative to β-Actin. Bar=200 μm, **p*<0.05, ***p*<0.01, ****p*<0.001.


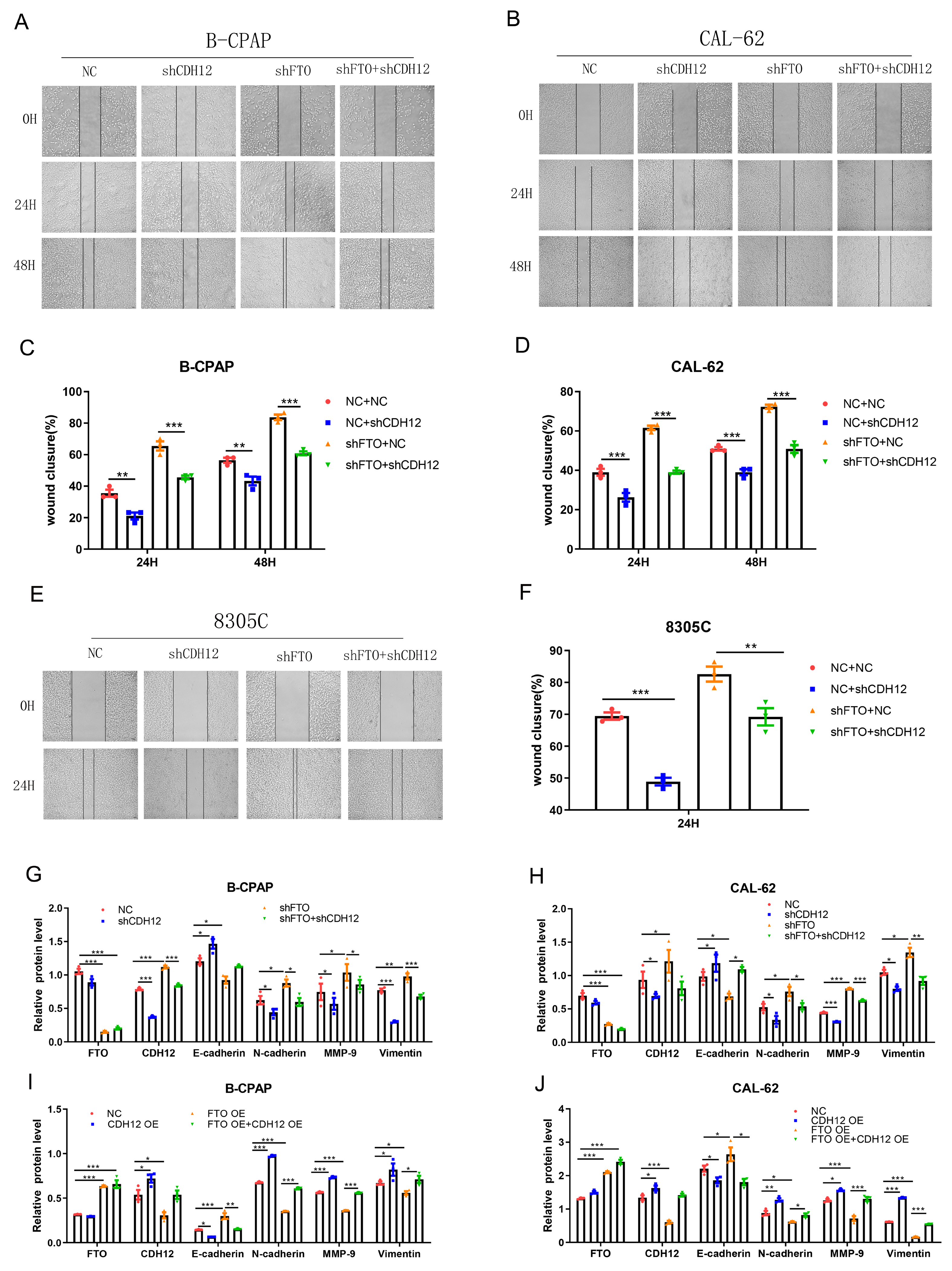


**Supplement Figure. 5 FTO inhibits the migration via CDH12 in thyroid carcinoma.** (A) The effects of shFTO and shCDH12 on the migration capability of B-CPAP, (B) CAL-62 and (E) 8305C cells with wound-healing assay were recorded and quantitatively analyzed (C, D and F). (G-H) Semiquantitative analysis of shFTO and shCDH12 on the protein levels of FTO, CDH12, E-cadherin, N-cadherin, MMP-9, and Vimentin in B-CPAP and CAL-62 cells relative to β-Actin. (I-J) Semiquantitative analysis of FTO OE and CDH12 OE on the protein levels of FTO, CDH12, E-cadherin, N-cadherin, MMP-9, and Vimentin in B-CPAP and CAL-62 cells relative to β-Actin. Bar=200 μm, **p*<0.05, ***p*<0.01, ****p*<0.001.


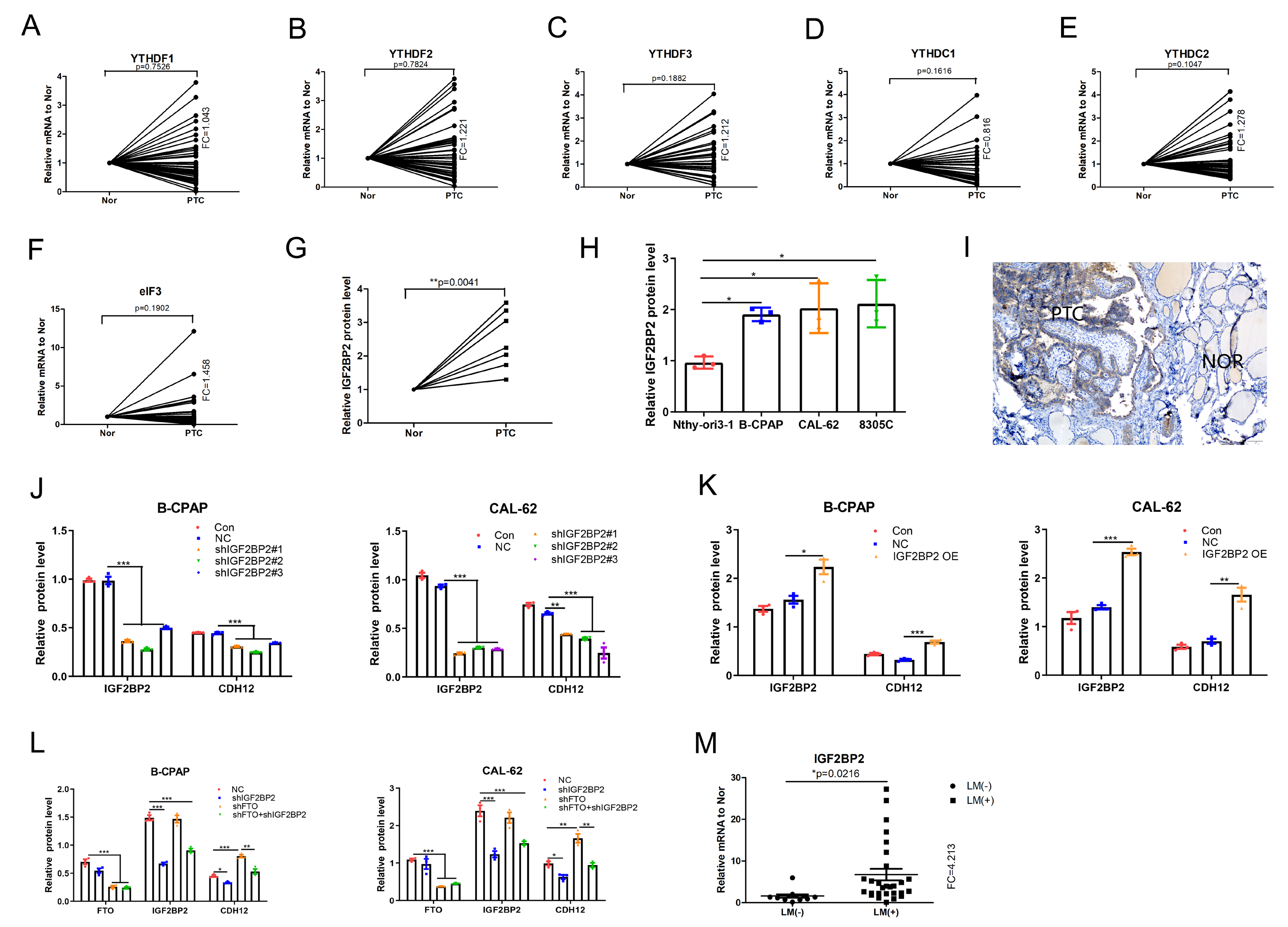


**Supplement Figure. 6 The expression of m6A readers in thyroid cancer.** (A-F) The mRNA levels of m6A reader protein in papillary thyroid carcinoma (PTC) tissues and the adjacent normal tissues (Nor). (G) Semiquantitative analysis of the IGF2BP2 protein levels of PTC and Nor tissues relative to GAPDH. (H) Semiquantitative analysis of the IGF2BP2 protein levels of Nthy-ori 3-1, B-CPAP , CAL-62, and 8305C relative to β-Actin. (I) Representative immunohistochemistry images of IGF2BP2 positive cells in PTC and IGF2BP2 negative cells in adjacent normal tissues from the same section. (J) Semiquantitative analysis of shIGF2BP2 or (K) IGF2BP2 OE on the protein levels of IGF2BP2 and CDH12 in B-CPAP and CAL-62 cells relative to β-Actin. (L) Semiquantitative analysis of shFTO and shIGF2BP2 on the protein levels of FTO, IGF2BP2 and CDH12 in B-CPAP and CAL-62 cells relative to β-Actin. (M) The mRNA level of IGF2BP2 in PTC tissues with (LM+) or without (LM-) cervical lymph node metastasis. Bar=200 μm. **p*<0.05, ***p*<0.01, ****p*<0.001.

**
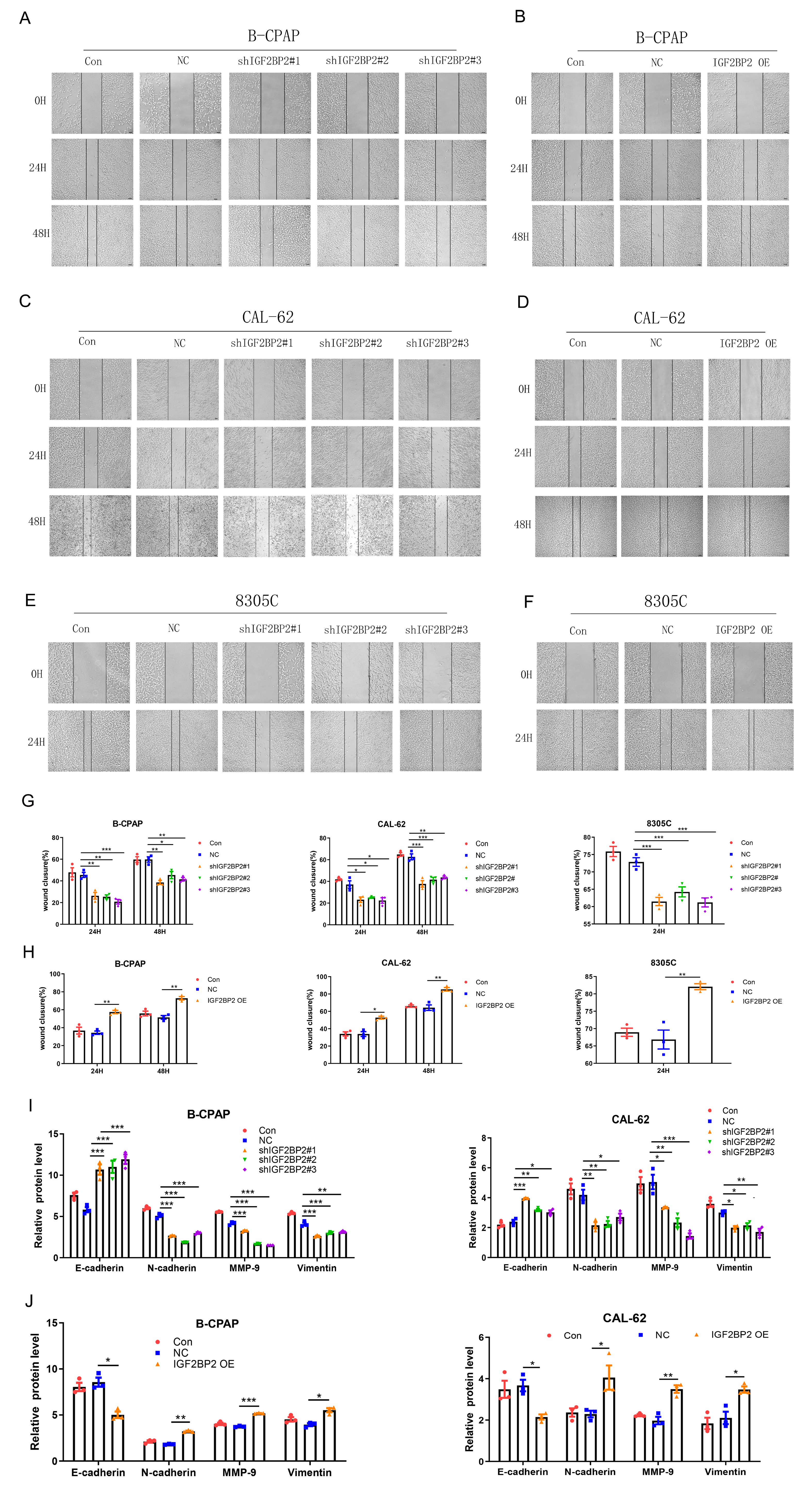
**

**Supplement Figure. 7 The role of IGF2BP2 on the migration of thyroid carcinoma.** (A) Effects of IGF2BP2 knockdown (shIGF2BP2) or (B) overexpression (IGF2BP2 OE) on the migration capability of B-CPAP with wound-healing assay were recorded and quantitatively analyzed (G-H). (C) Effects of shIGF2BP2 or (D) IGF2BP2 OE on the migration capability of CAL-62 with wound-healing assay were recorded and quantitatively analyzed (G-H). (E) Effects of shIGF2BP2 or (F) IGF2BP2 OE on the migration capability of 8305C with wound-healing assay were recorded and quantitatively analyzed (G-H). (I) Semiquantitative analysis of shIGF2BP2 or (J) IGF2BP2 OE on the protein levels of E-cadherin, N-cadherin, MMP-9, and Vimentin in B-CPAP and CAL-62 cells relative to β-Actin. Bar=200 μm, **p*<0.05, ***p*<0.01, ****p*<0.001.


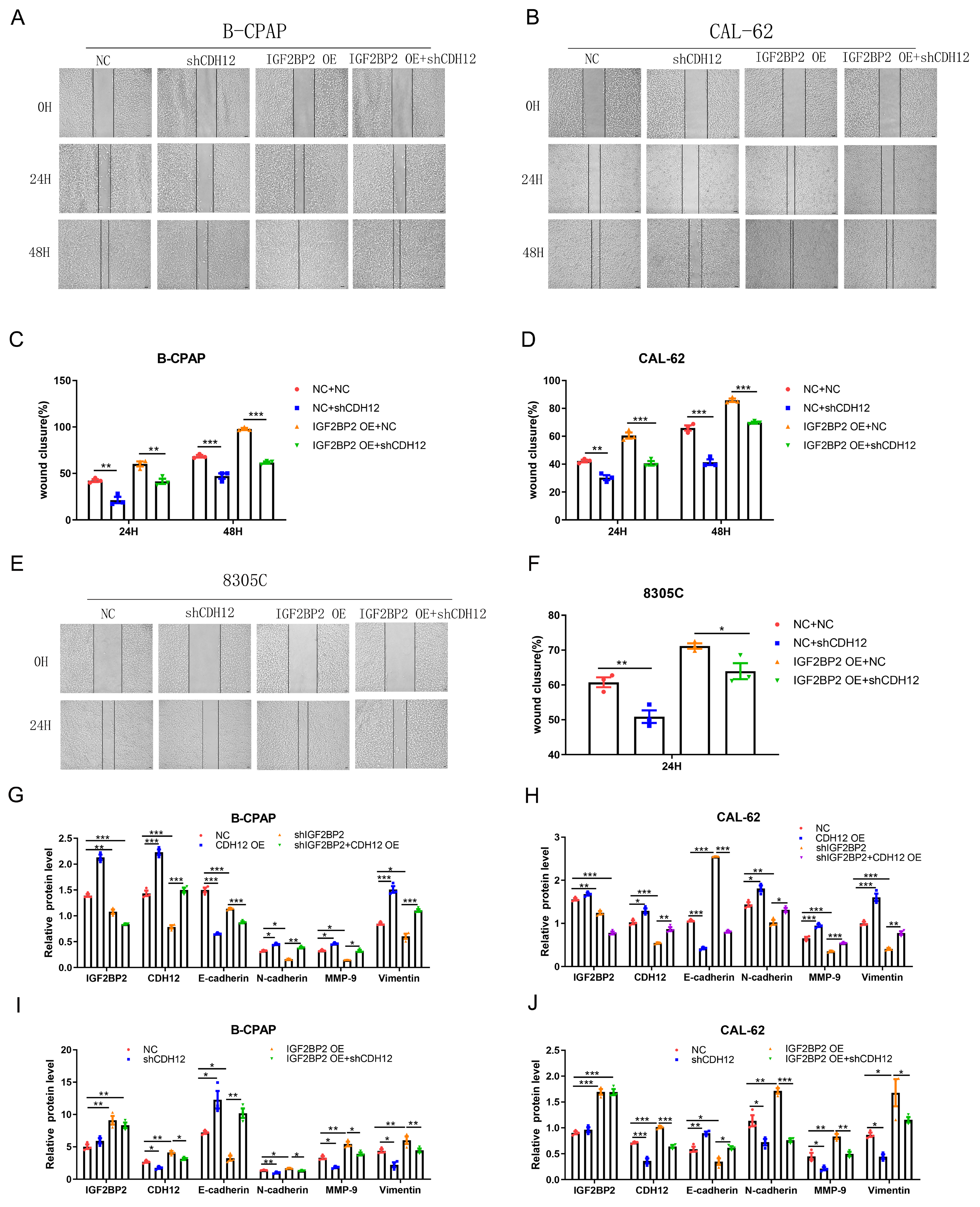


**Supplement Figure. 8 IGF2BP2 inhibits the migration via CDH12 in thyroid carcinoma.** (A)The effects of IGF2BP2 OE and shCDH12 on the migration capability of B-CPAP, (B) CAL-62 and (E) 8305Ccells with wound-healing assay were recorded and quantitatively analyzed (C-D and F). (G-H) Semiquantitative analysis of shIGF2BP2 and CDH12 OE on the protein levels of IGF2BP2, CDH12, E-cadherin, N-cadherin, MMP-9, and Vimentin in B-CPAP and CAL-62 cells relative to β-Actin. (I-J) Semiquantitative analysis of IGF2BP2 OE and shCDH12 on the protein levels of IGF2BP2, CDH12, E-cadherin, N-cadherin, MMP-9, and Vimentin in B-CPAP and CAL-62 cells relative to β-Actin. Bar=200 μm, **p*<0.05, ***p*<0.01, ****p*<0.001.
