# Supplementary material for: FTO/IGF2BP2-mediated N6 methyladenosine modification in invasion and metastasis of thyroid carcinoma via CDH12
Source: Cell Death Dis. 2024 Oct 8;15(10):733. doi: 10.1038/s41419-024-07097-4 (PMC11461506; doi:10.1038/s41419-024-07097-4)
Supplement: Supplementary file 2 — Supplement2-original Western blot bands [file 41419_2024_7097_MOESM2_ESM.pdf]

The uncropped picture of Western Blotting

Figure 1

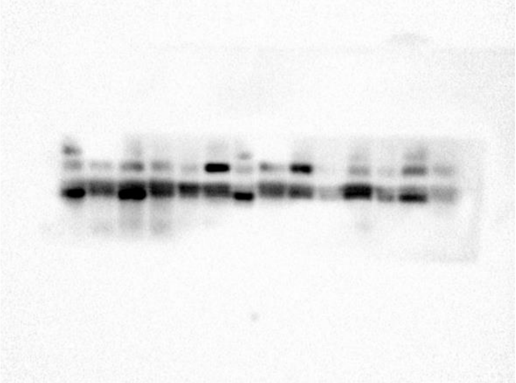

Figur1E-FTO

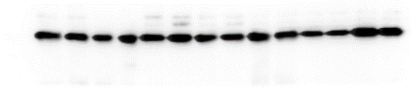

Figur1E-GAPDH

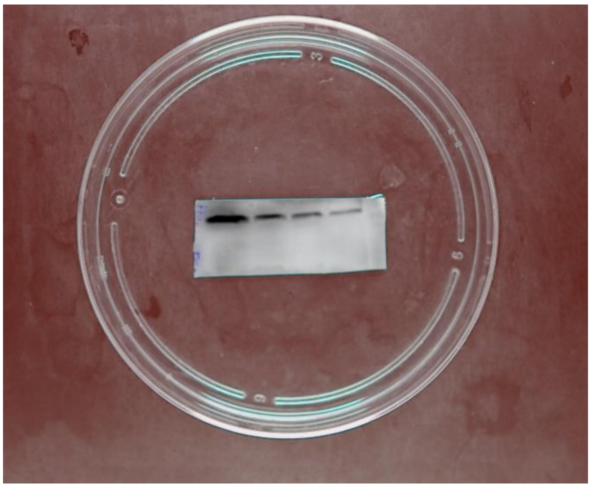

Figure1F-FTO

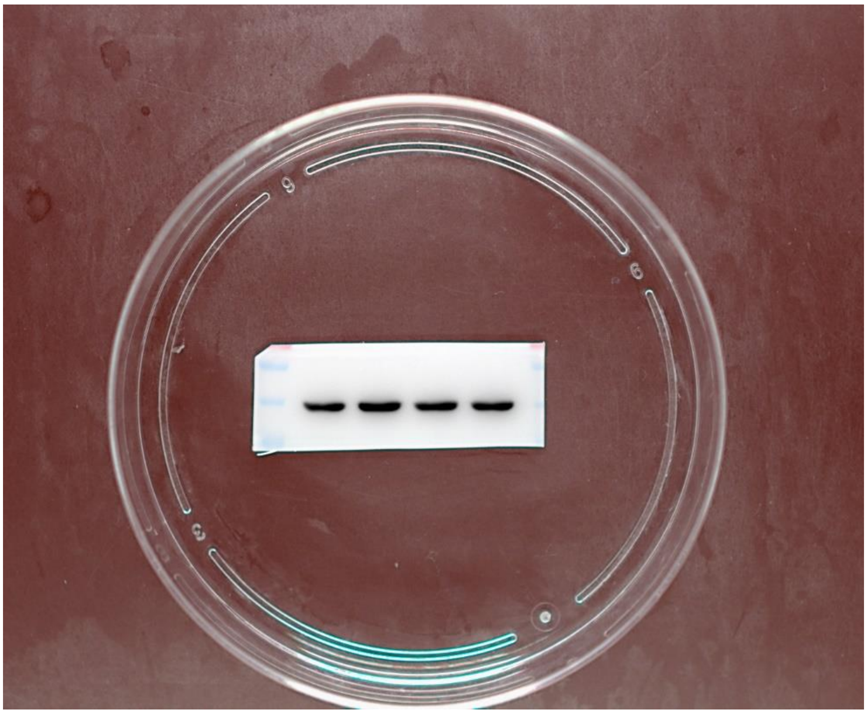

Figure1F-Actin

Figure 2

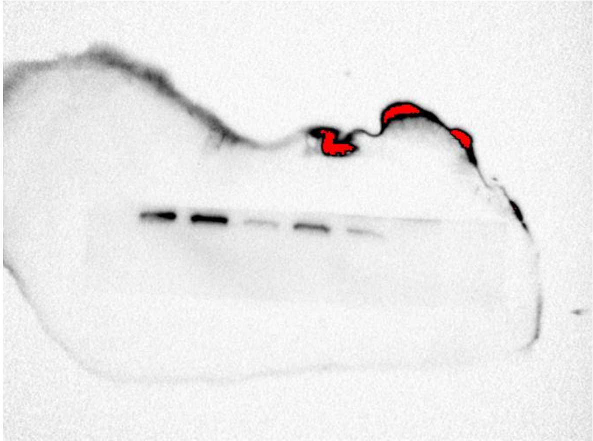

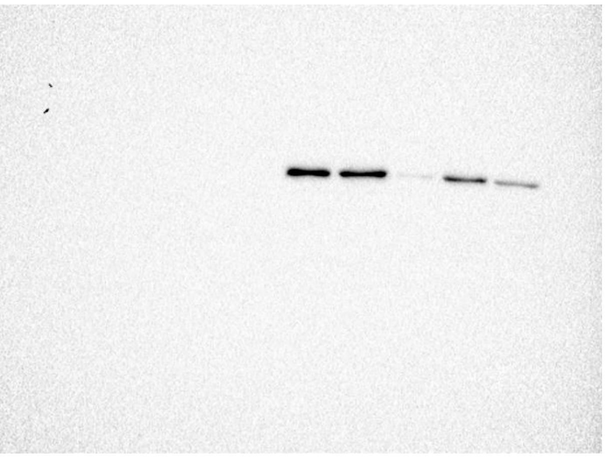

Figure2G-FTO

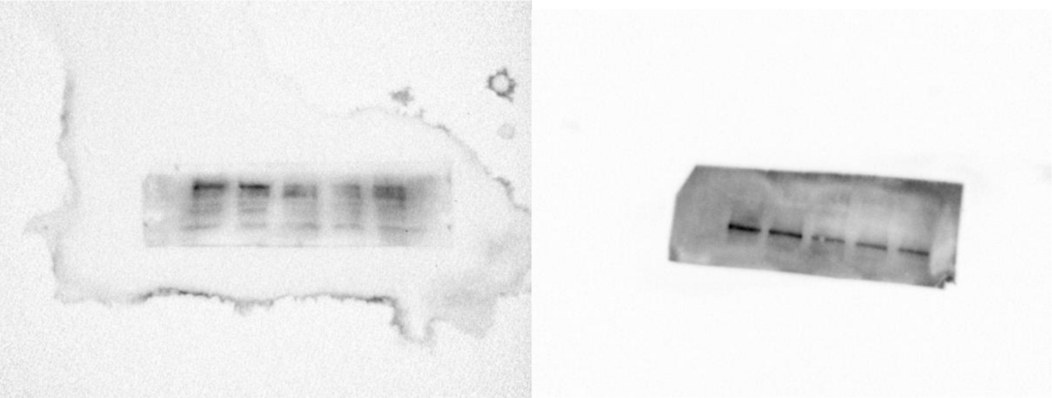

Figure2G-E-cadherin

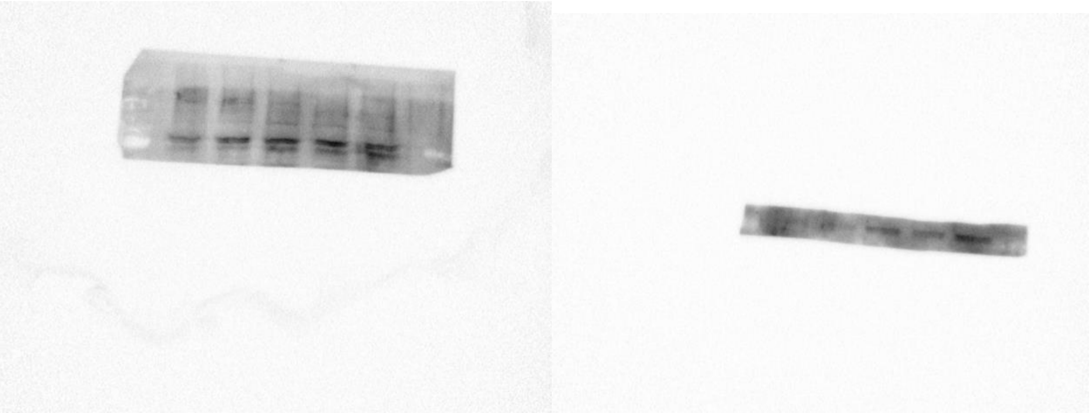

Figure2G-N-cadherin

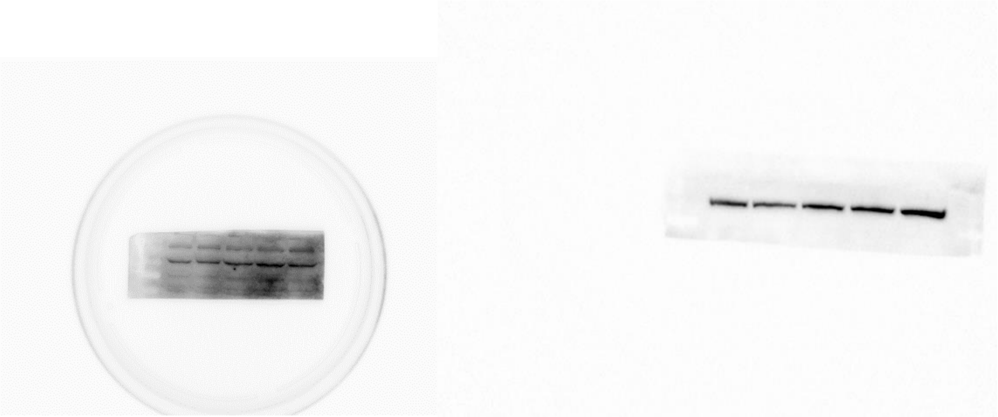

Figure2G-MMP-9

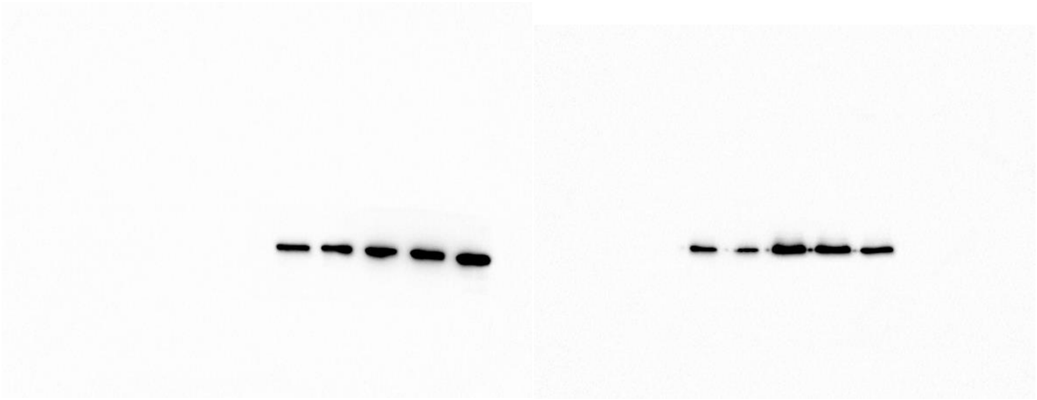

Figure2G-Vimentin

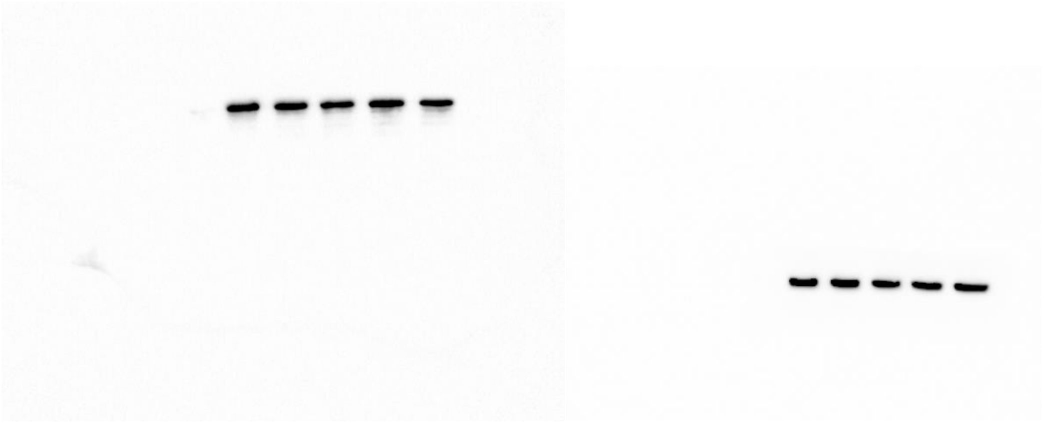

Figure2G-Actin

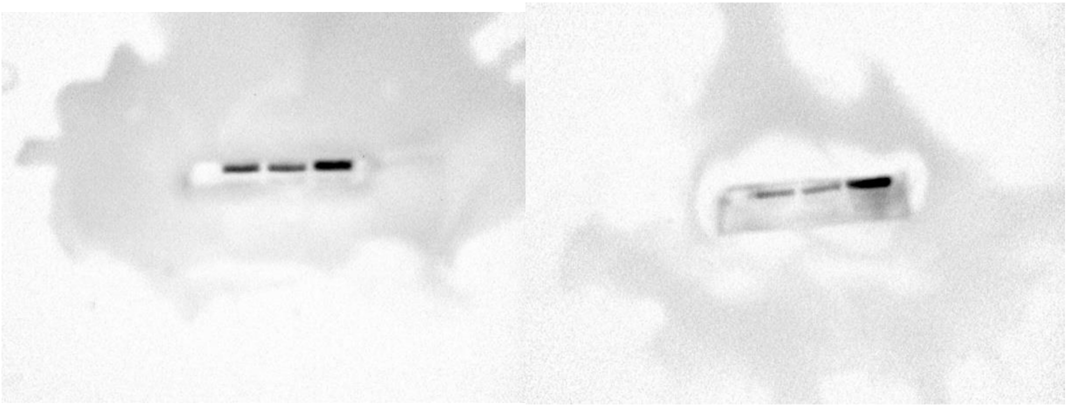

Figure2H-FTO

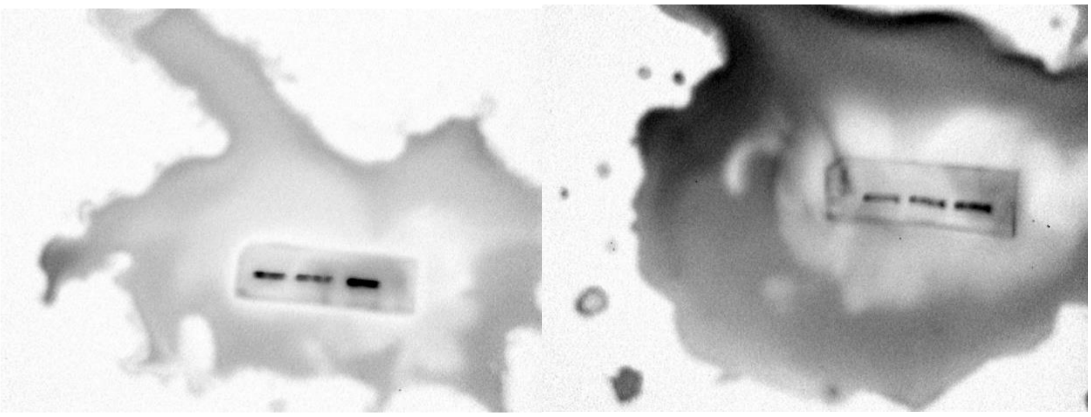

Figure2H-E-cadherin

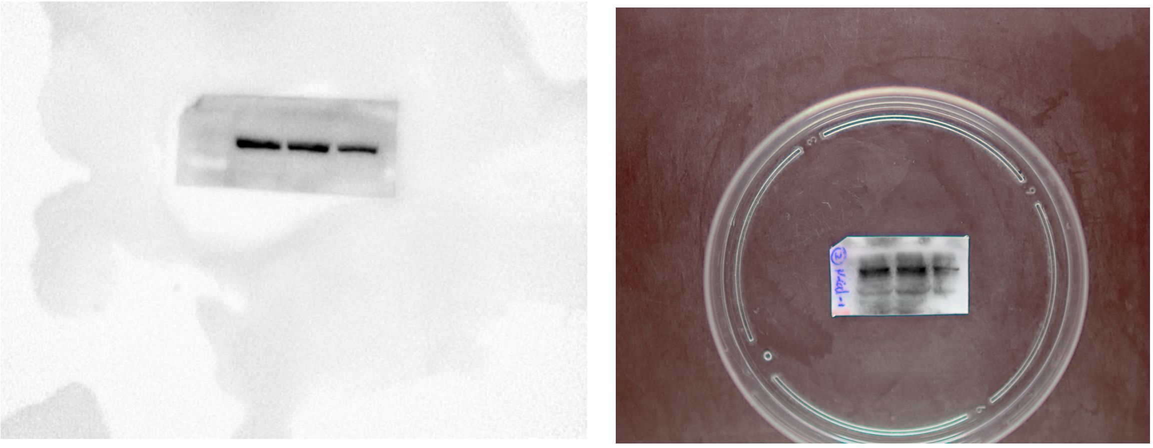

Figure2H-N-cadherin

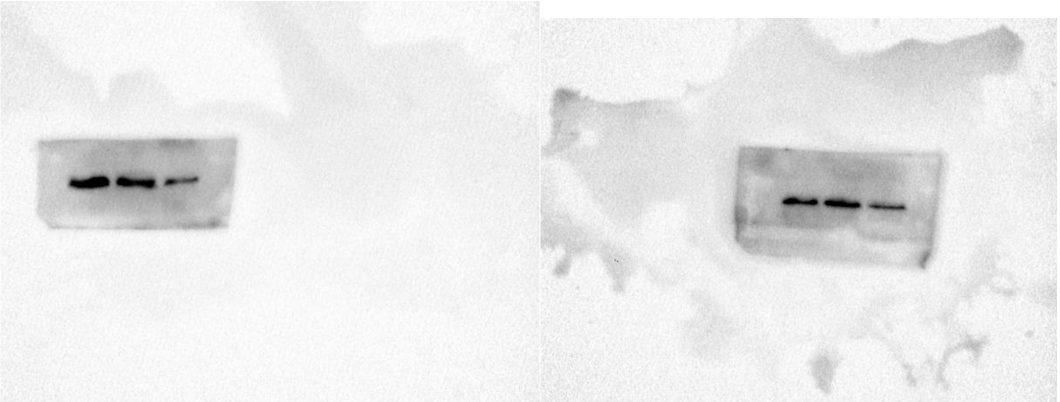

Figure2H-MMP-9

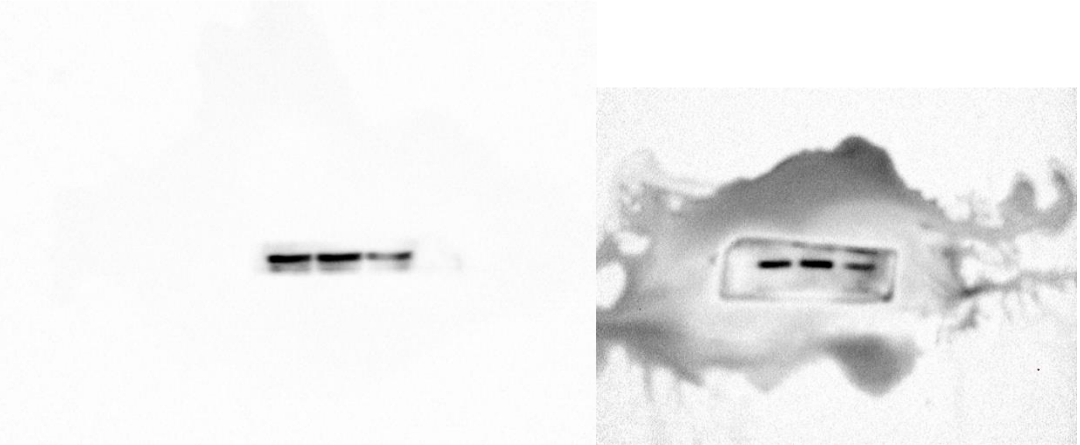

Figure2H-Vimentin

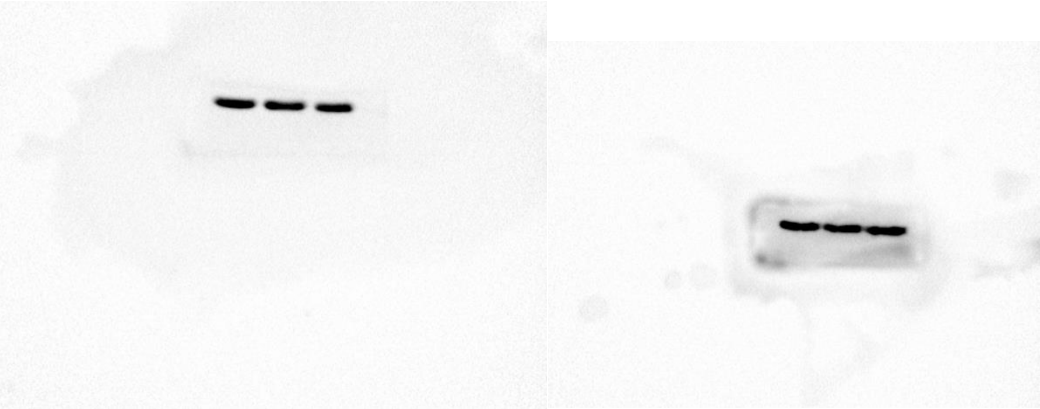

Figure2H-Actin

Figure 3

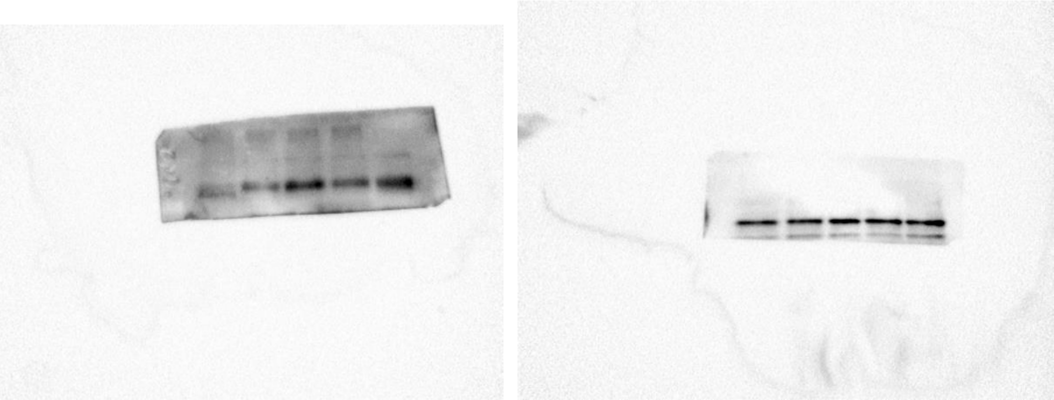

Figure3E-CDH12

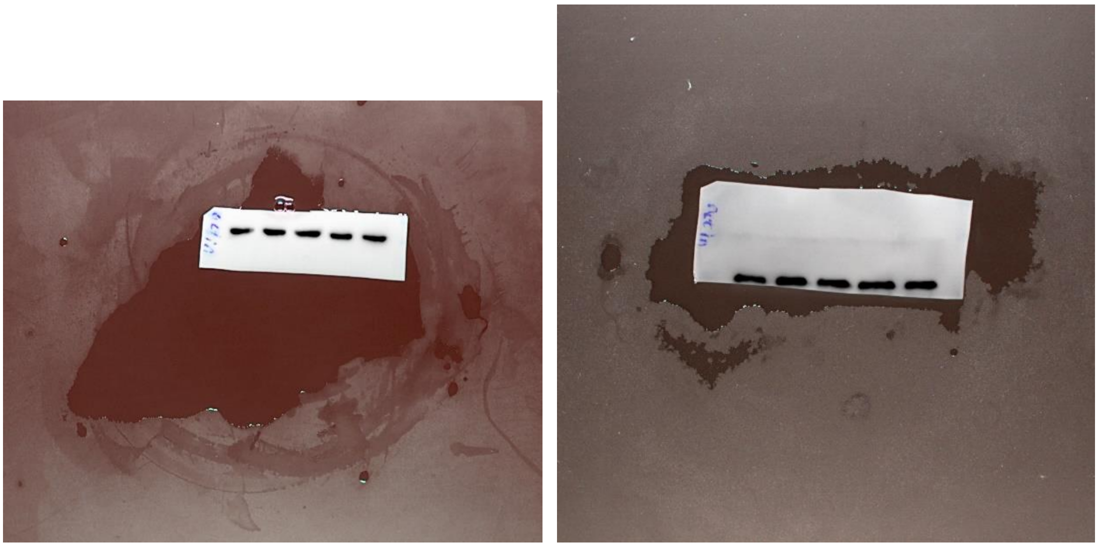

Figure3E-Actin

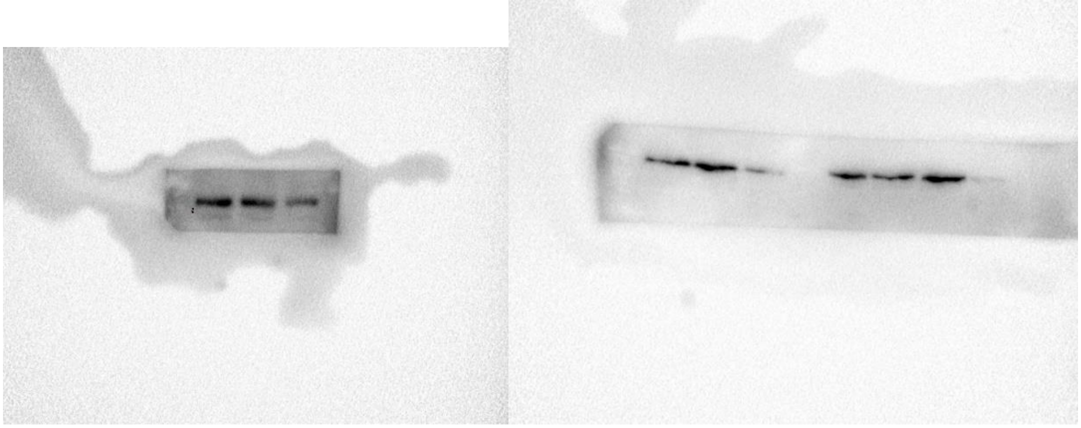

Figure3F-CDH12

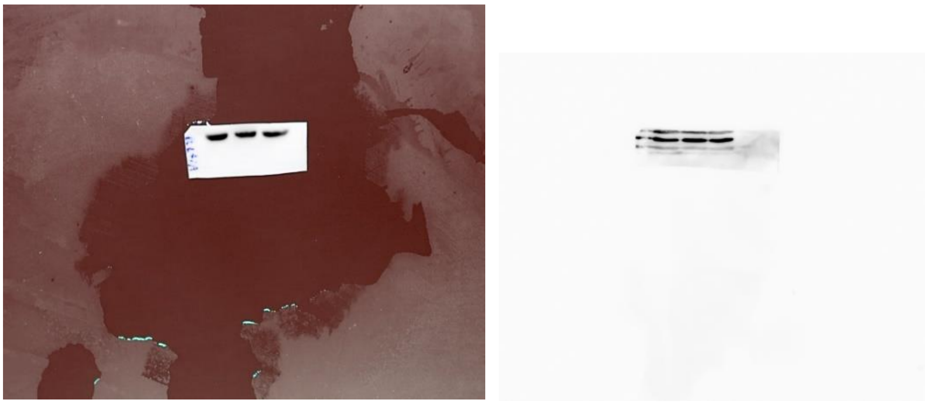

Figure3F-Actin

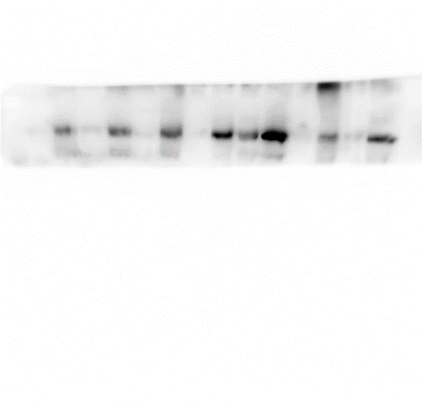

Figure3G-CDH12

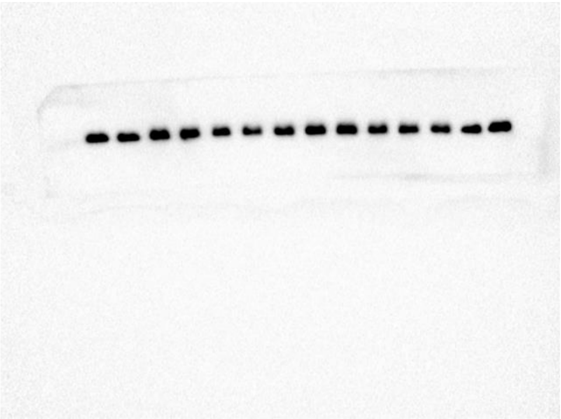

Figure3G-GAPDH

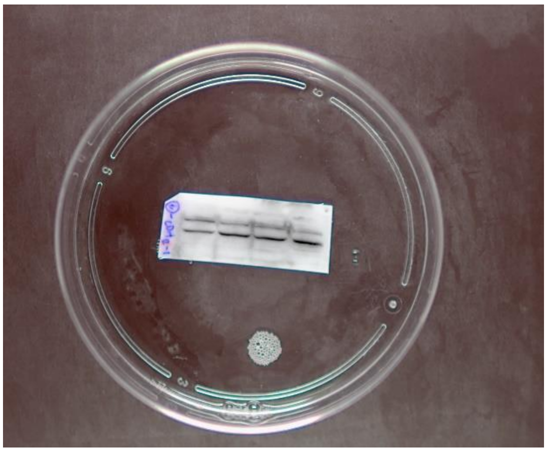

Figure3H-CDH12

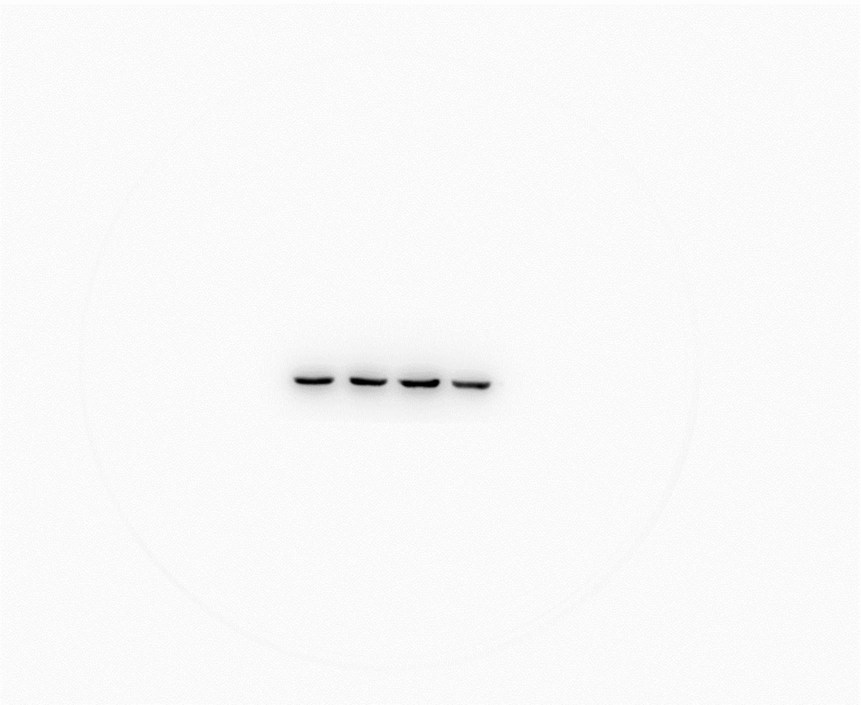

Figure3H-Actin

Figure 4

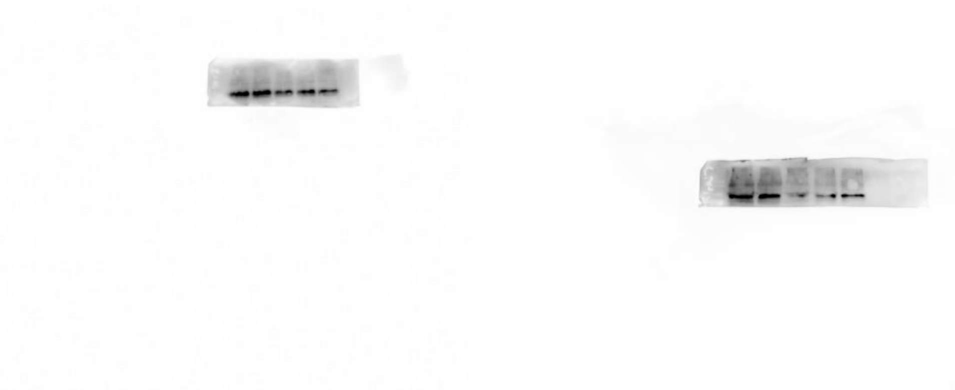

Figure4G-CDH12

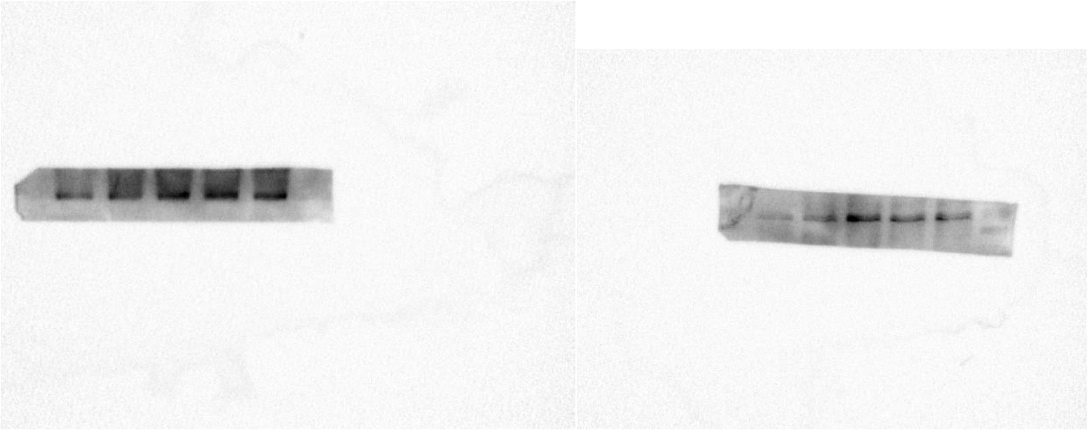

Figure4G-E-cadherin

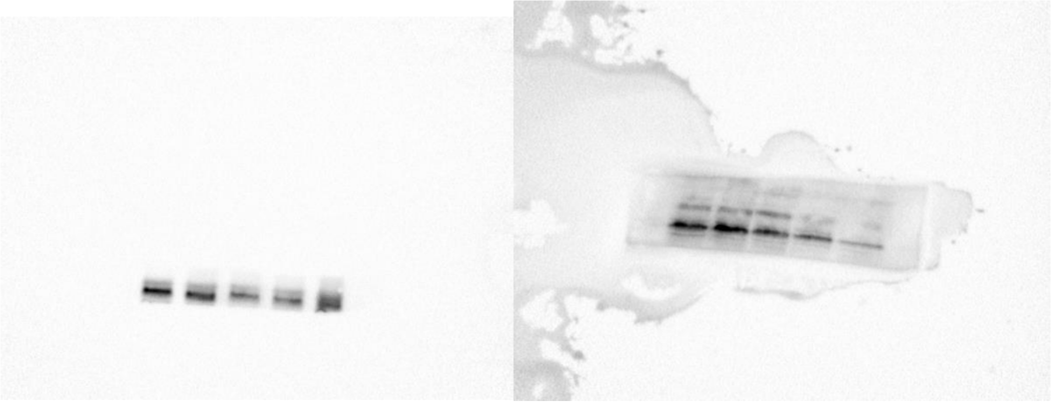

Figure4G-N-cadherin

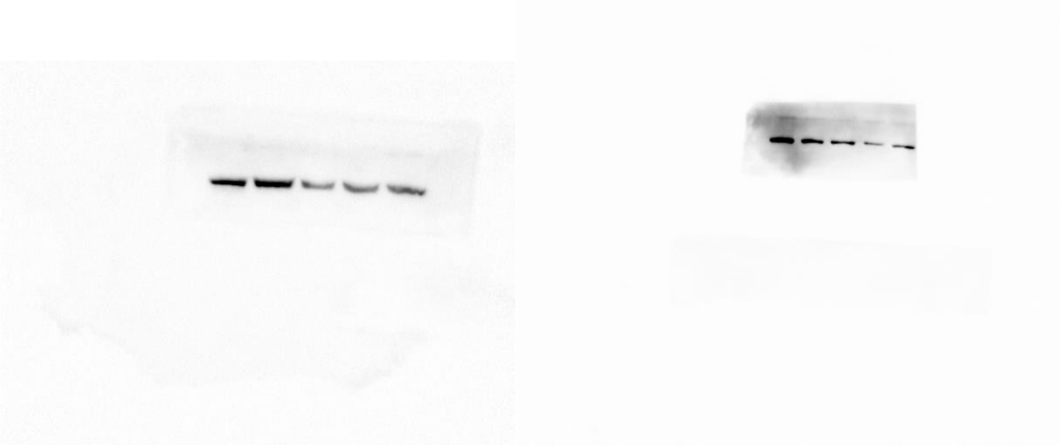

Figure4G-MMP-9

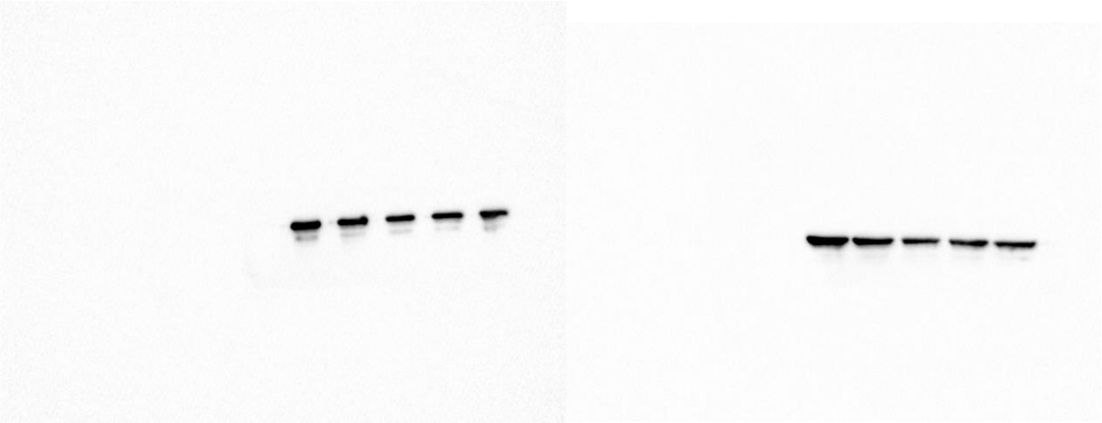

Figure4G-Vimentin

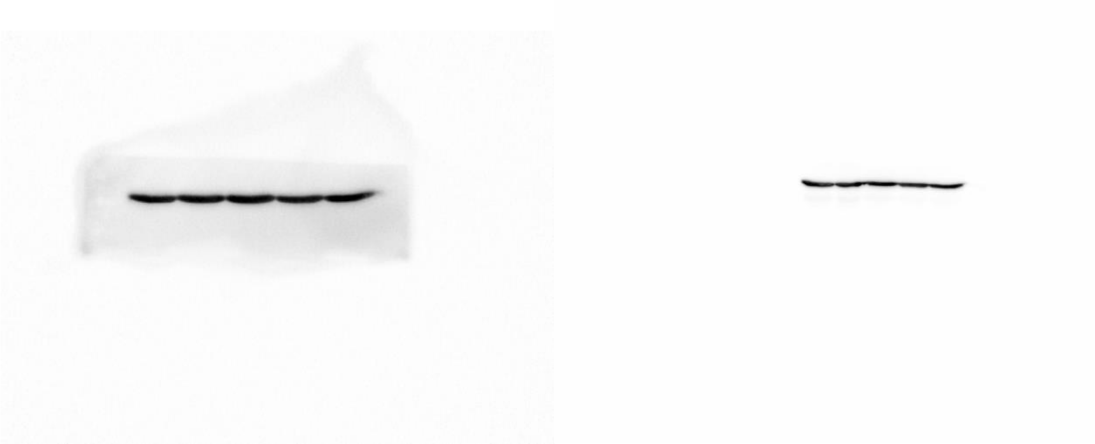

Figure4G-Actin

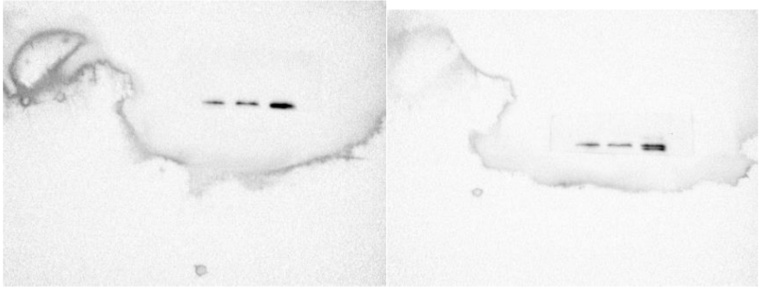

Figure4H-CDH12

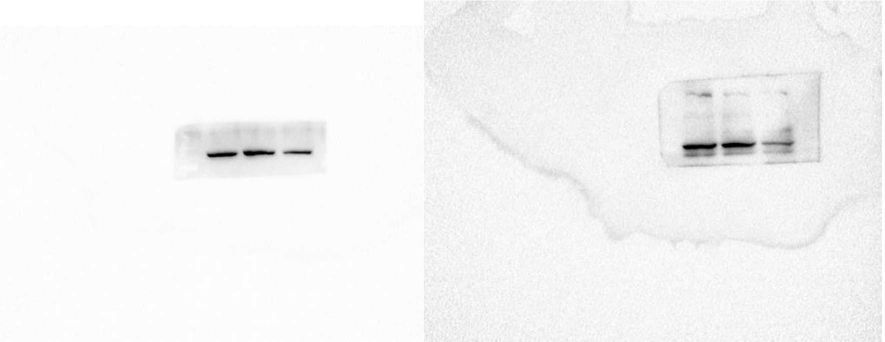

Figure4H-CDH12

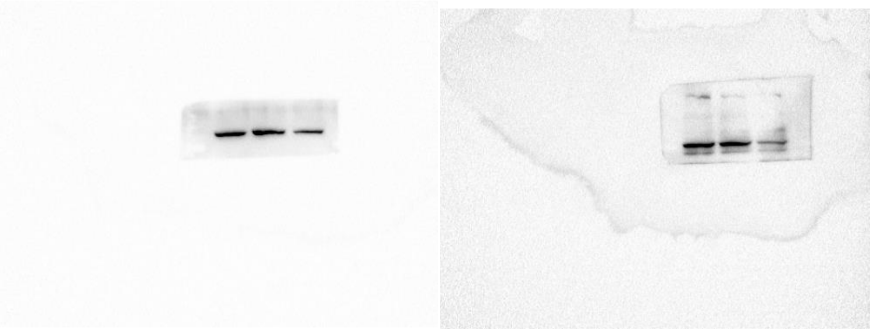

Figure4H-E-cadherin

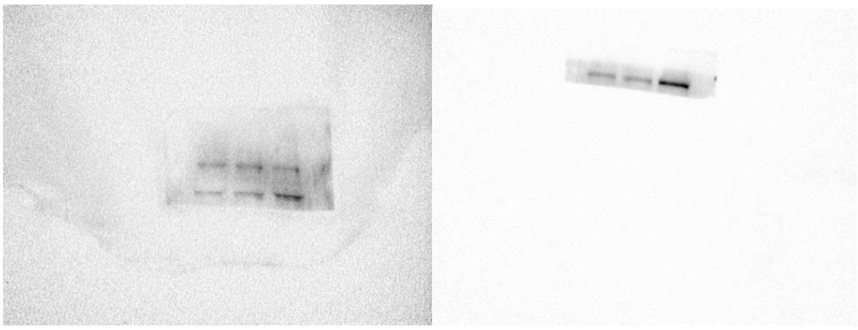

Figure4H-N-cadherin

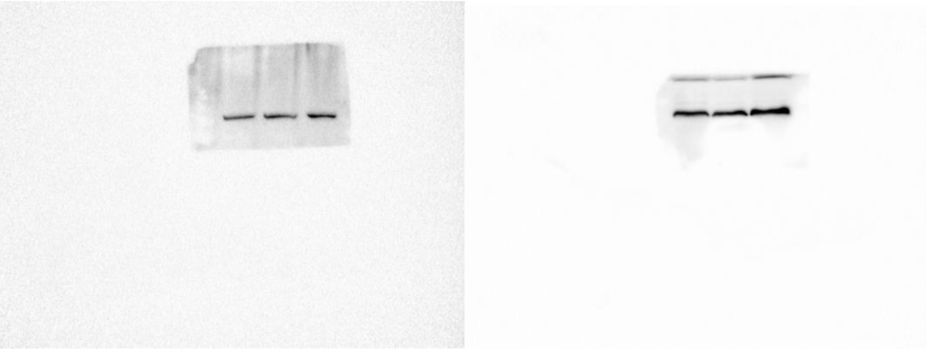

Figure4H-MMP-9

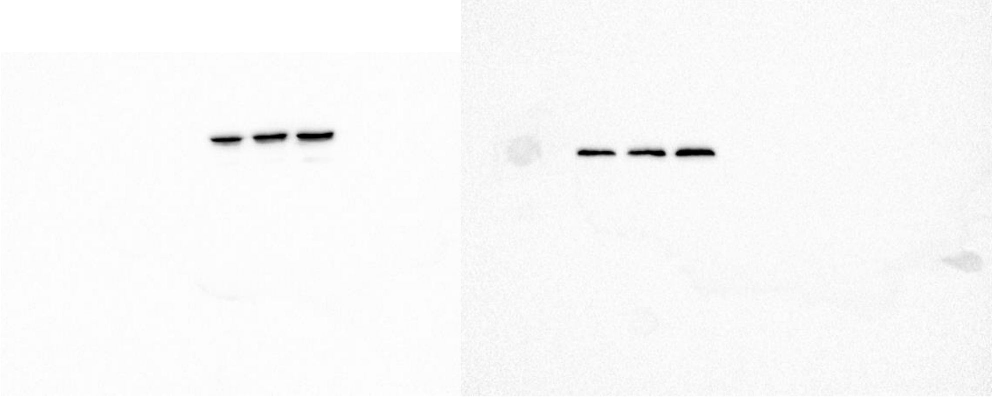

Figure4H-Vimentin

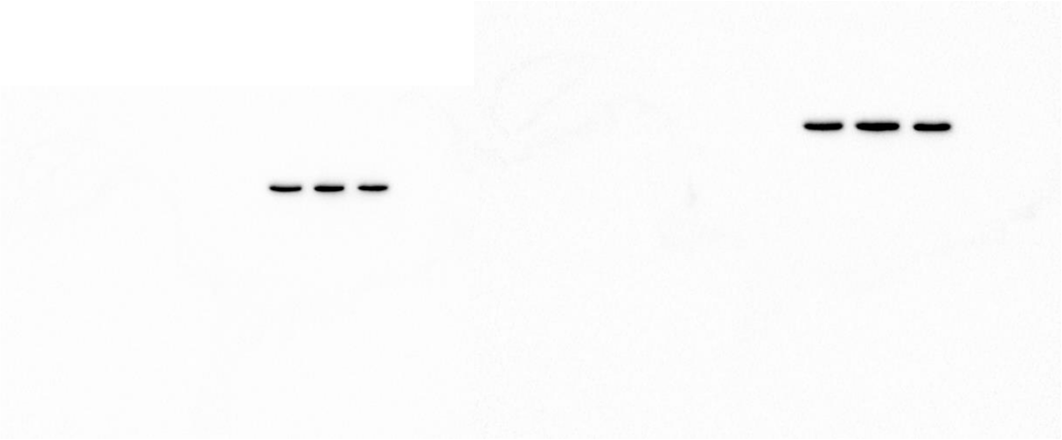

Figure4H-Actin

Figure 5

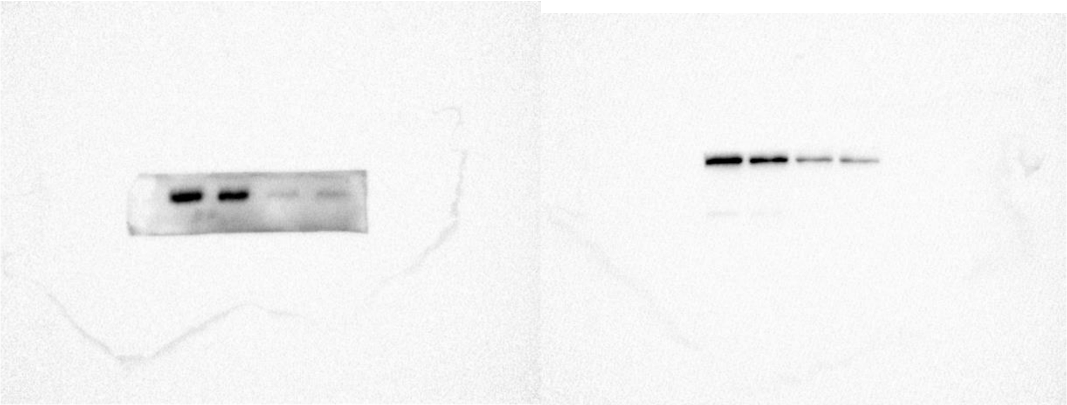

Figure5EFTO

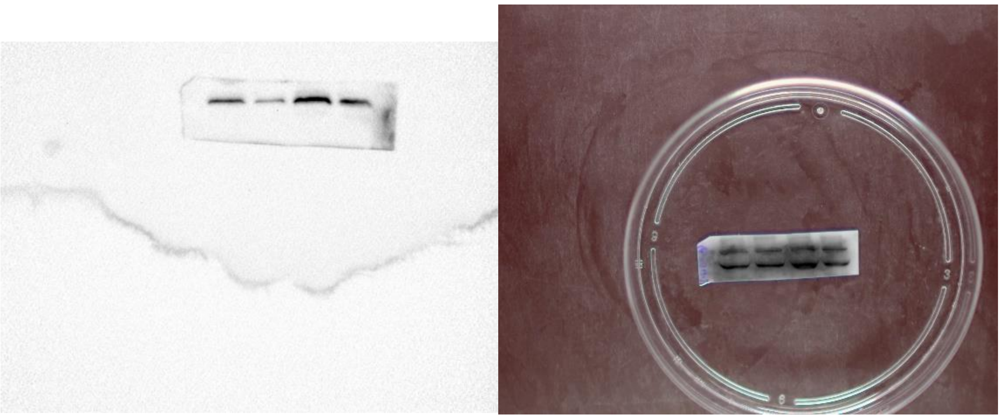

Figure5E-CDH12

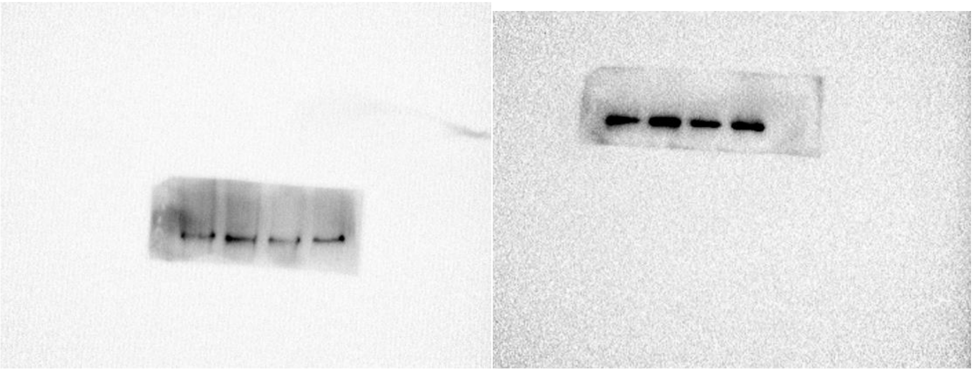

Figure5E-E-cadherin

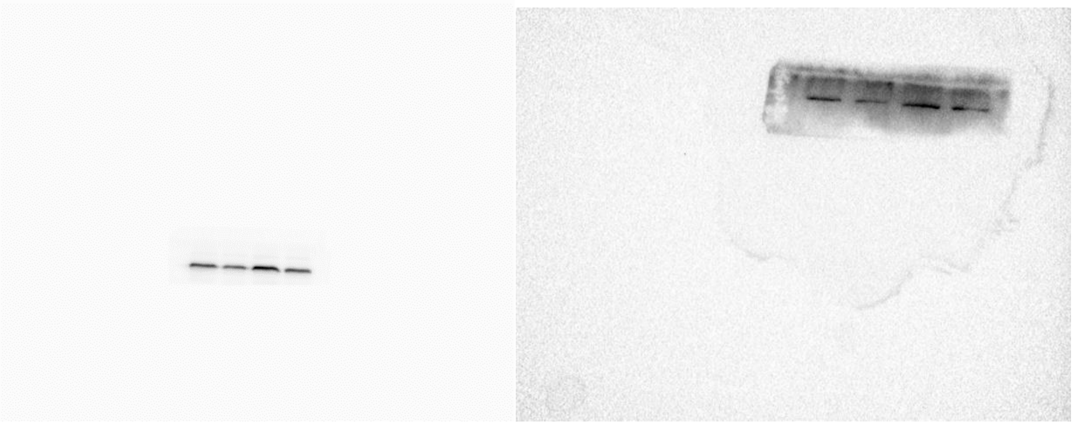

Figure5E-N-cadherin

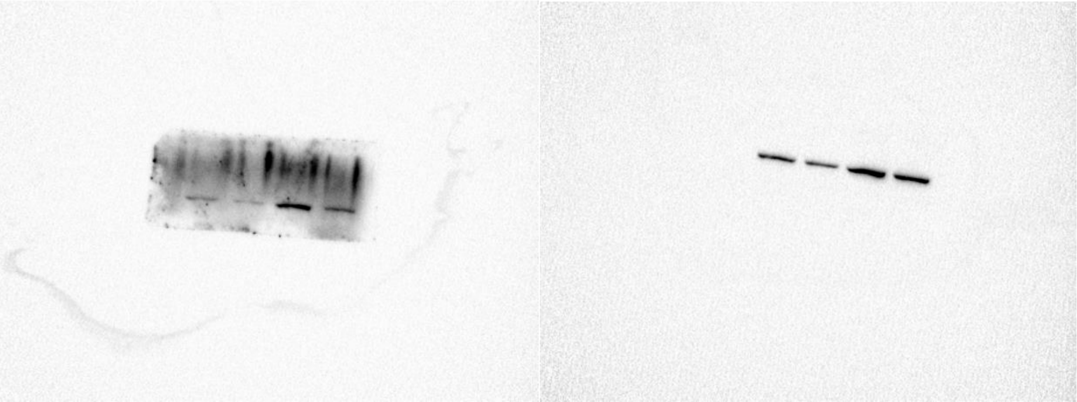

Figure5E-MMP-9

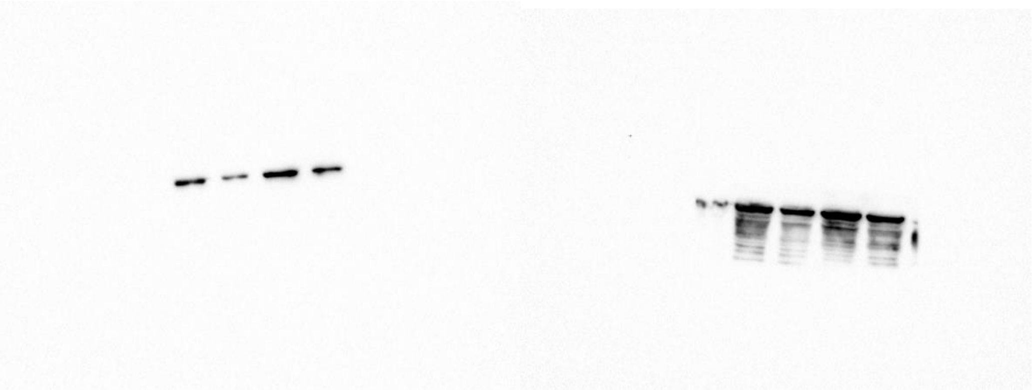

Figure5E-Vimentin

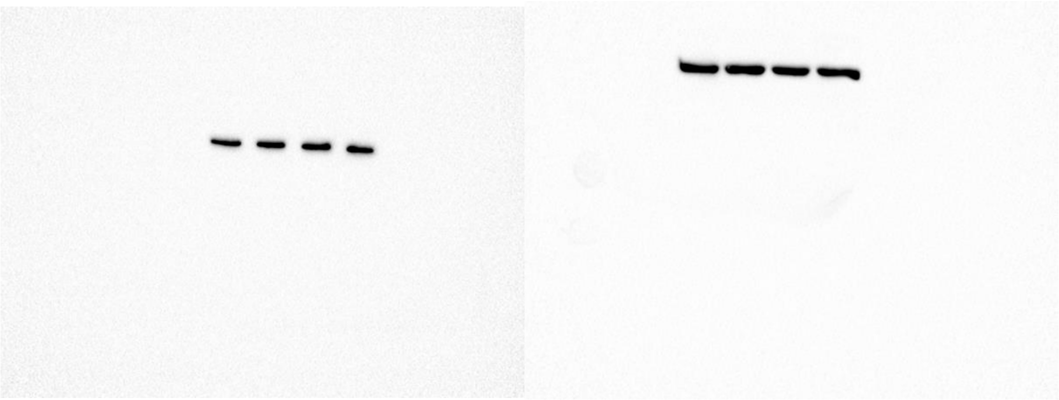

Figure5E-Actin

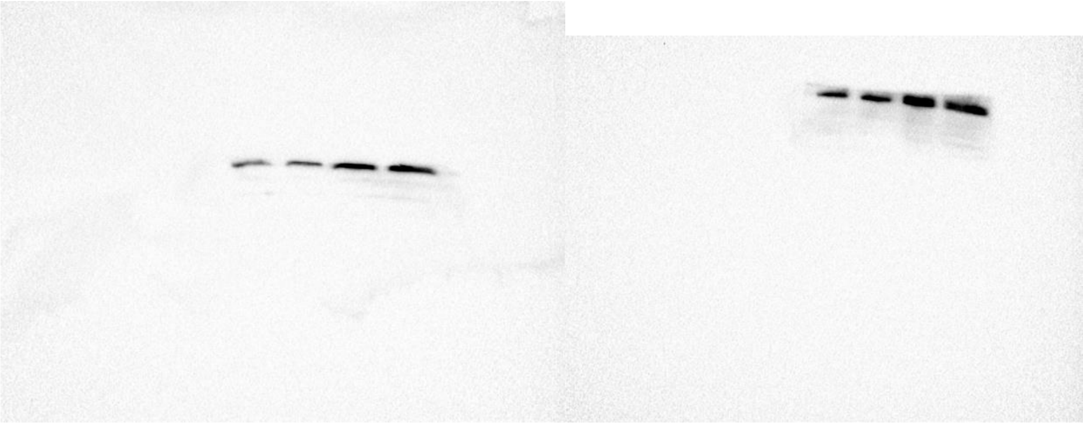

Figure5F-FTO

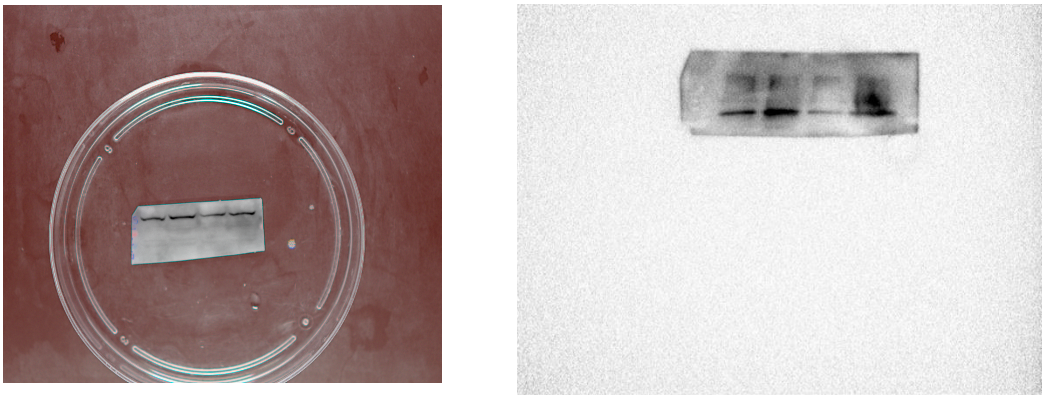

Figure5F-CDH12

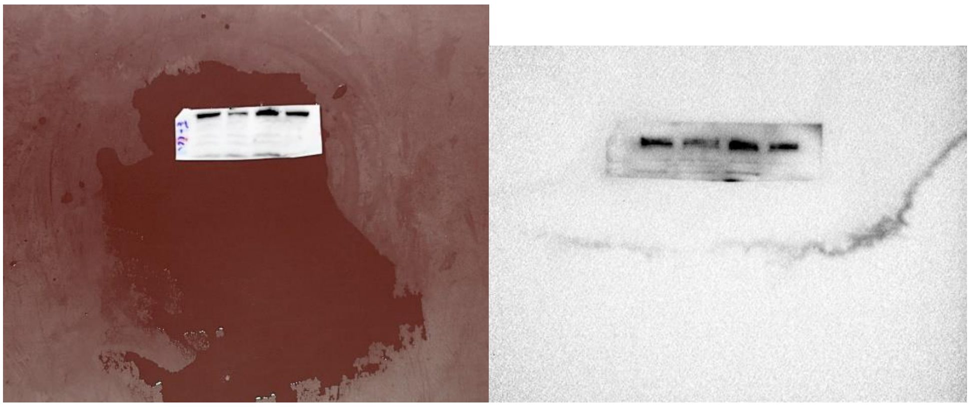

Figure5F-E-cadherin

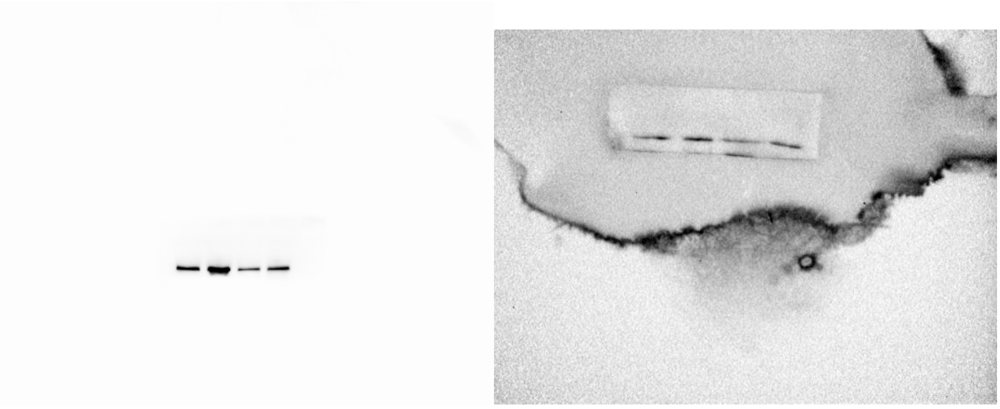

Figure5F-N-cadherin

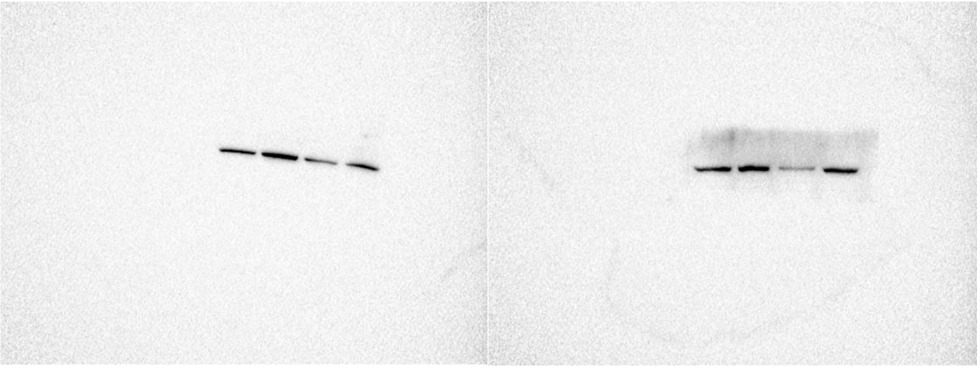

Figure5F-MMP-9

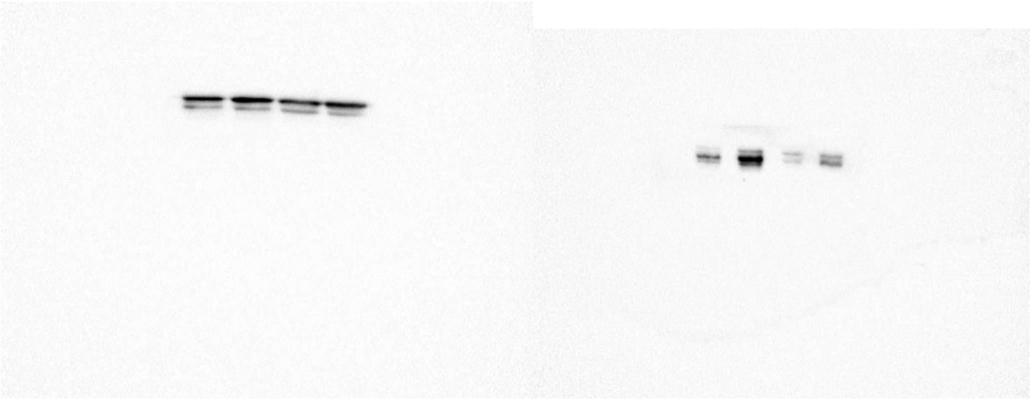

Figure5F-Vimentin

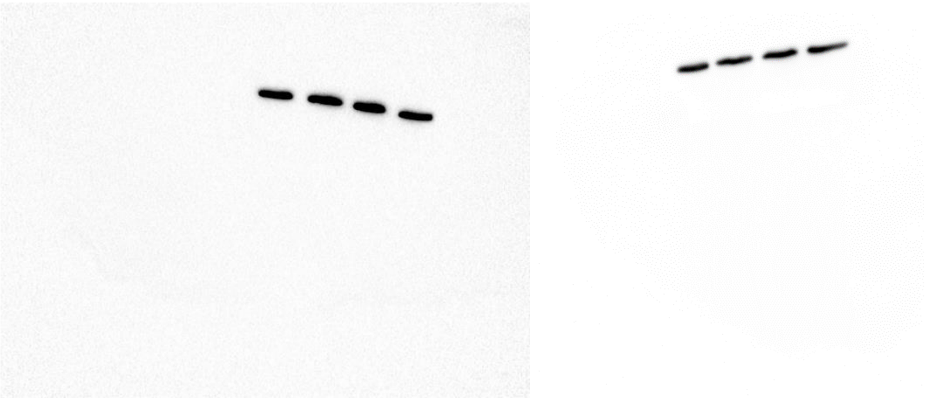

Figure5F-Actin

Figure 6

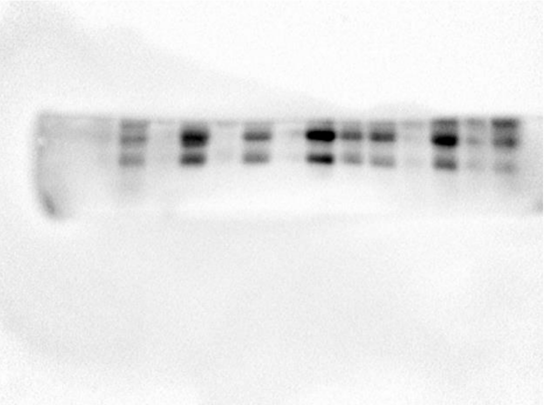

Figure6B-IGF2BP2

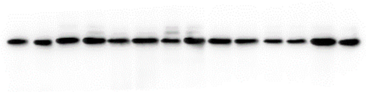

Figure6B-GAPDH

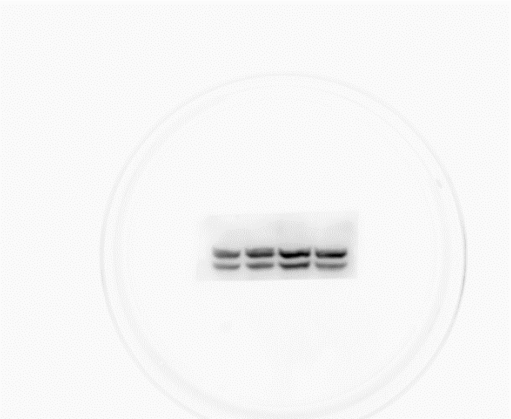

Figure6C-IGF2BP2

Figure6C-Actin

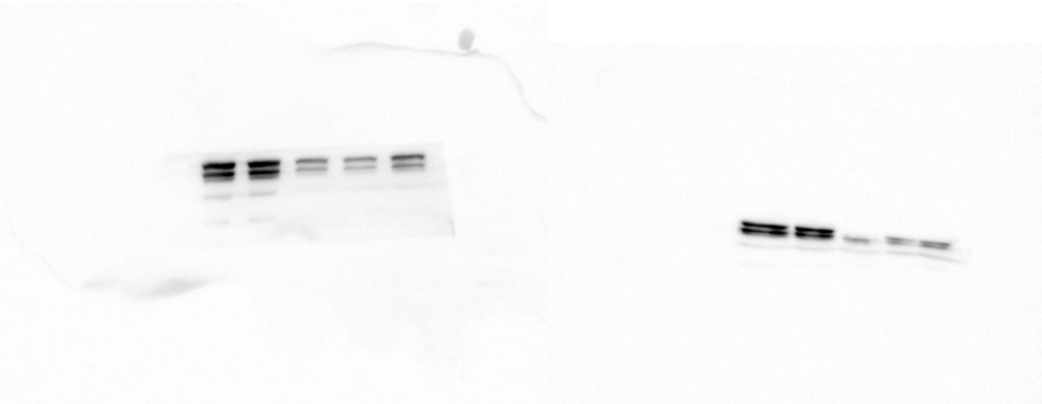

Figure 6I-IGF2BP2

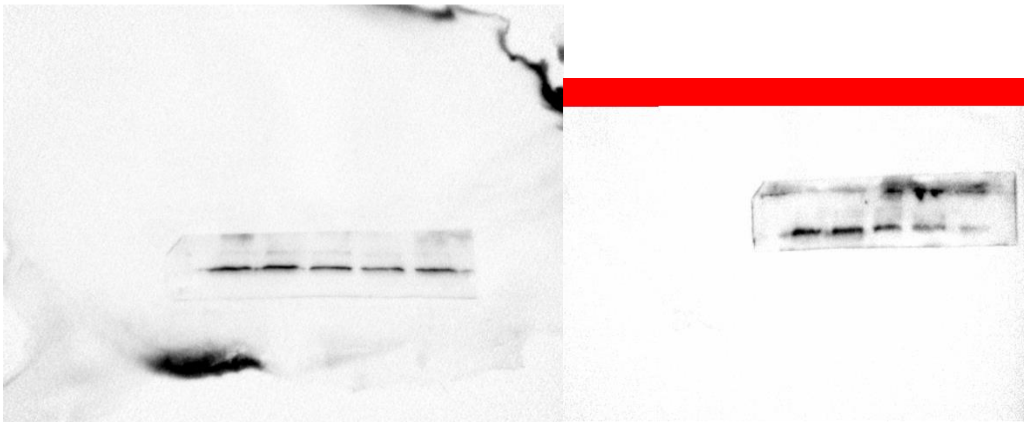

Figure 6I-CDH12

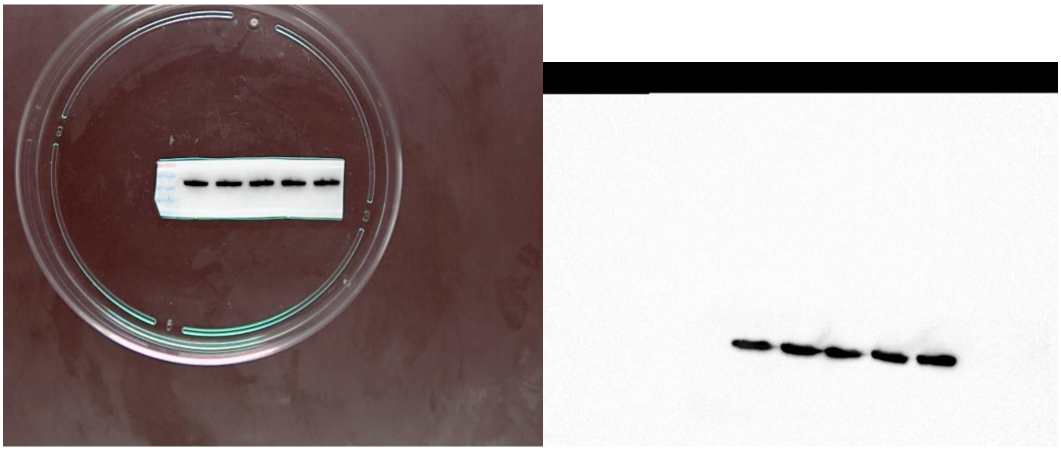

Figure 6I-Actin

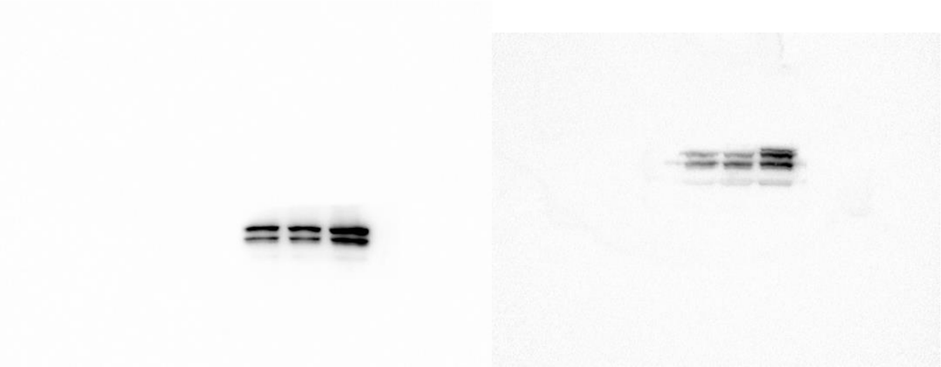

Figure 6I-IGF2BP2

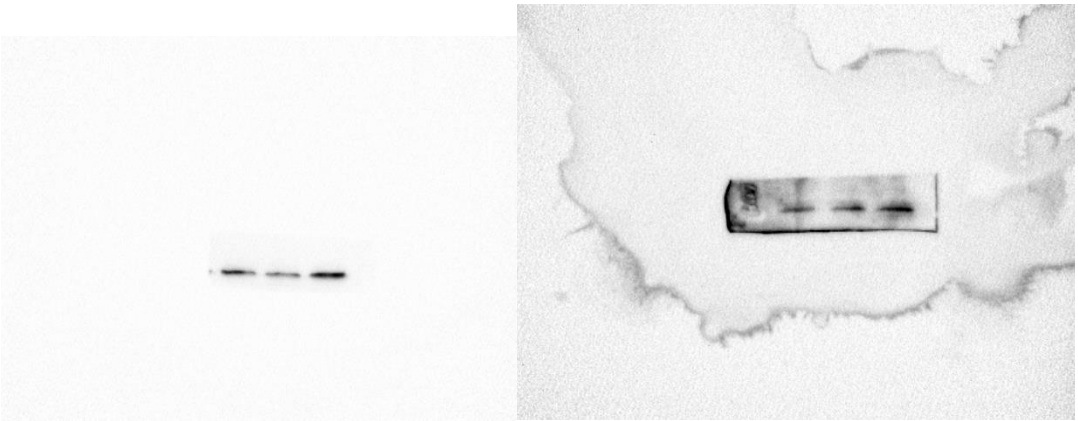

Figure 6J-CDH12

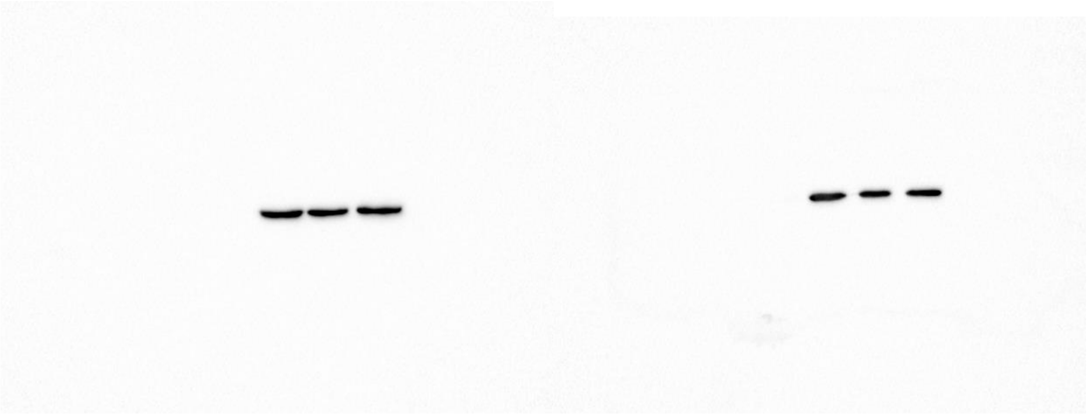

Figure 6J-Actin

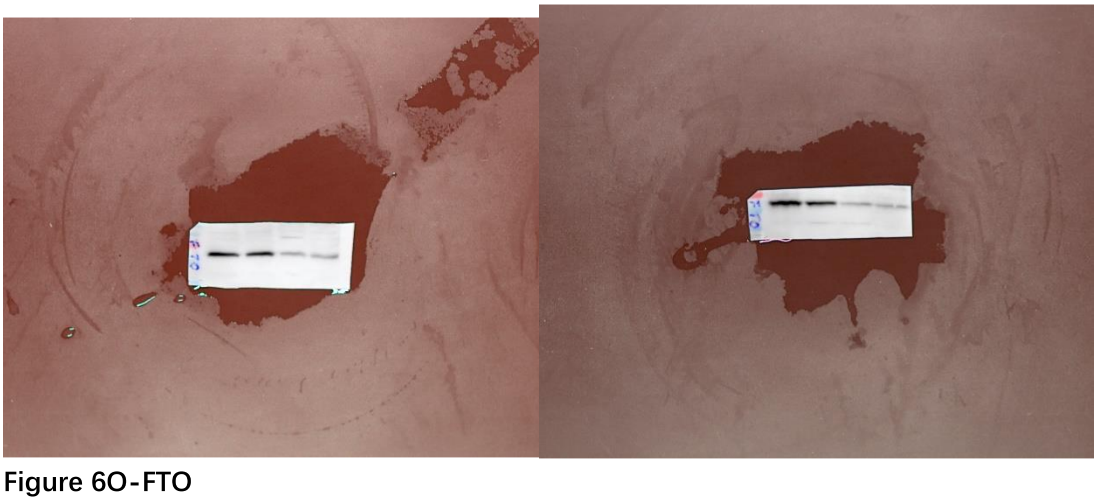

Figure 6O-FTO

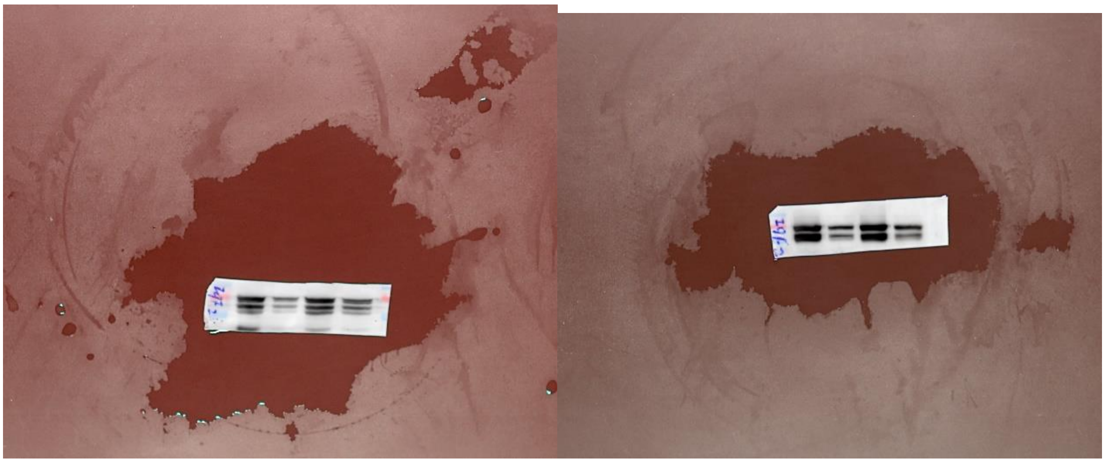

Figure 6O-IGF2BP2

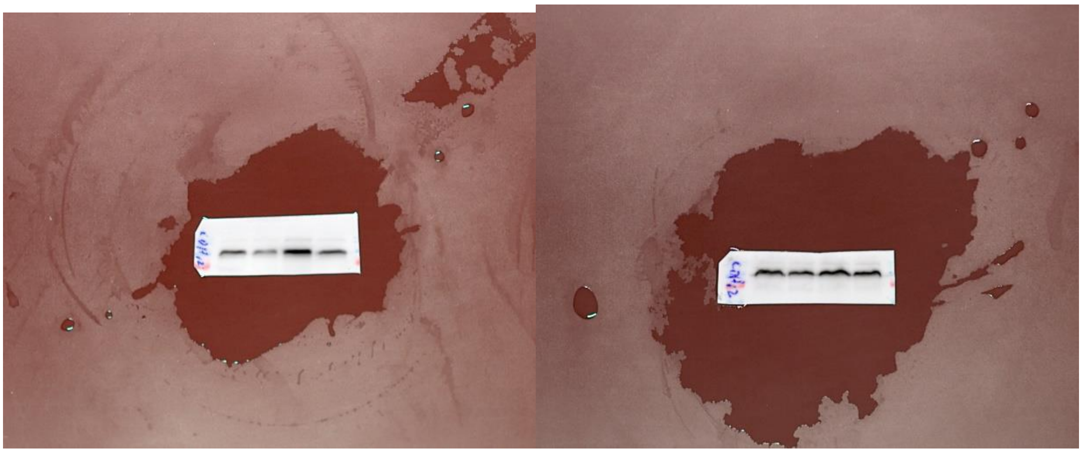

Figure 6O-CDH12

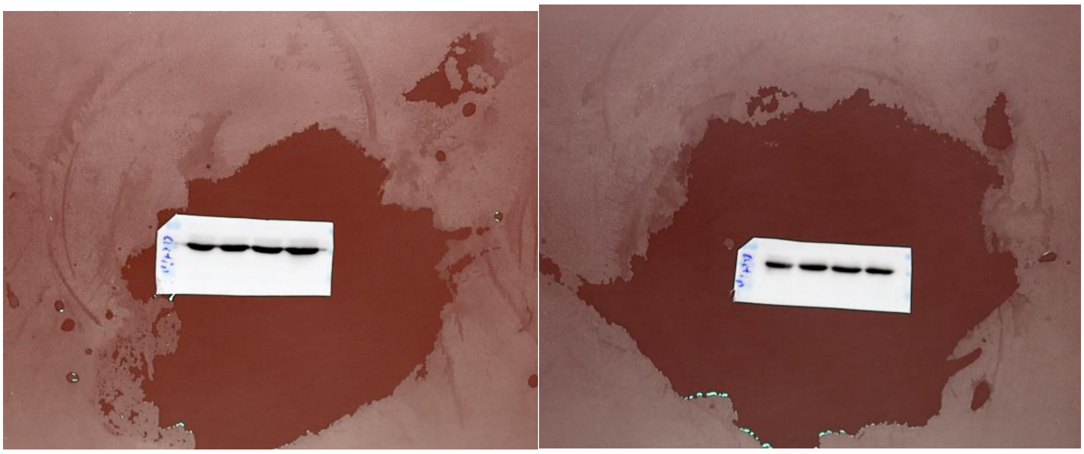

Figure 6O-Actin

Figure 7

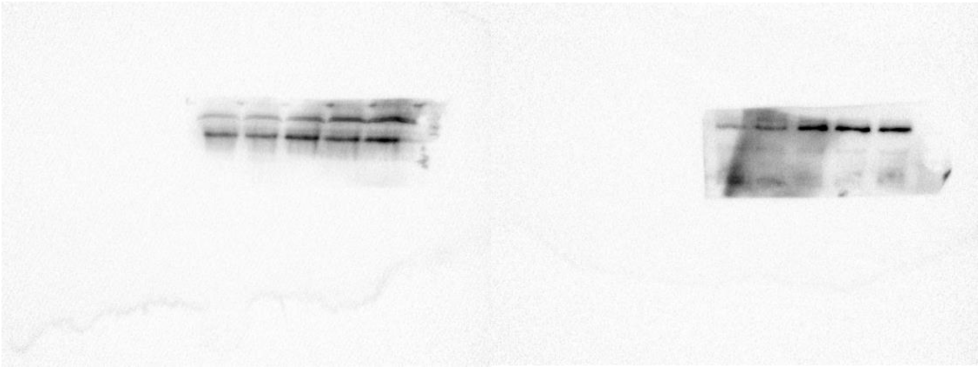

Figure7G-E-cadherin

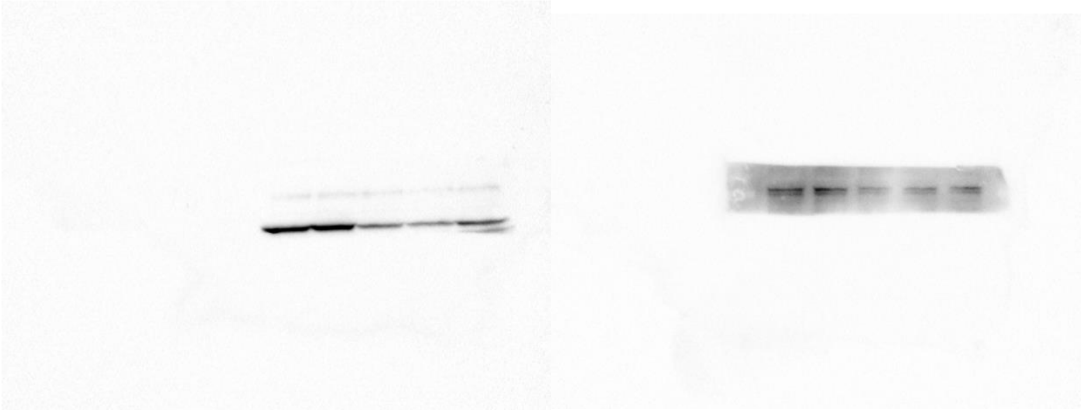

Figure7G-N-cadherin

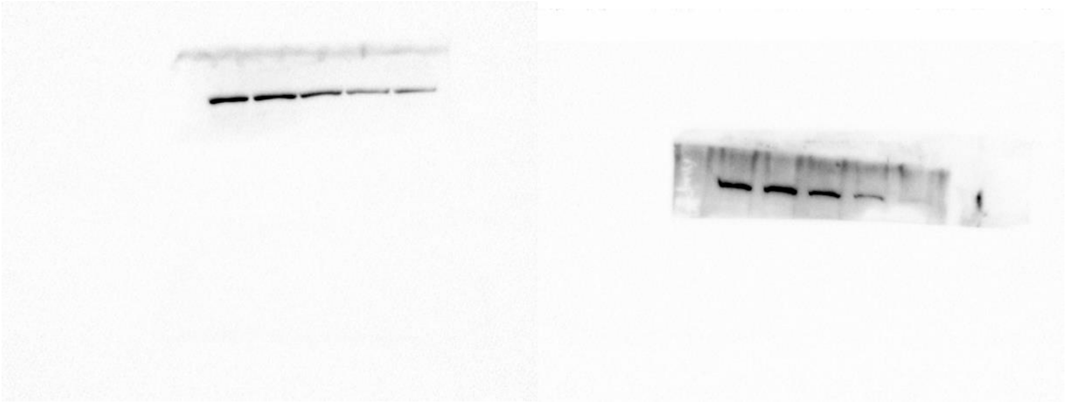

Figure7G -MMP-9

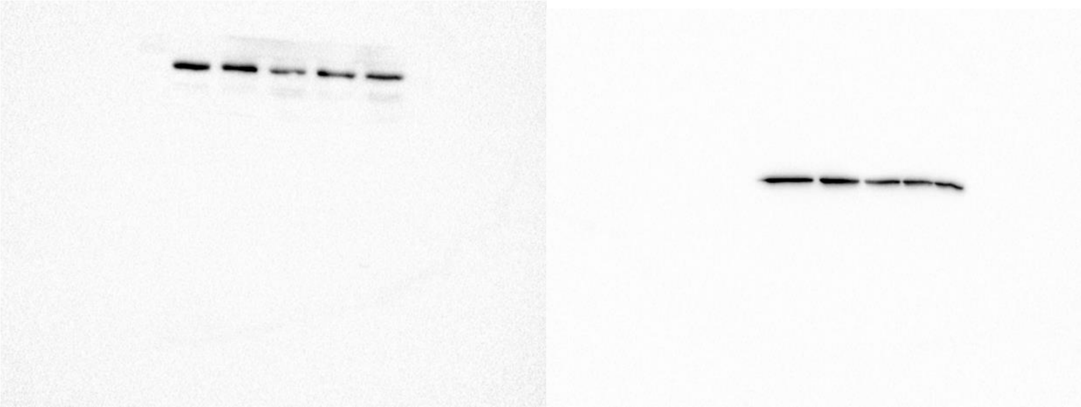

Figure7G-Vimentin

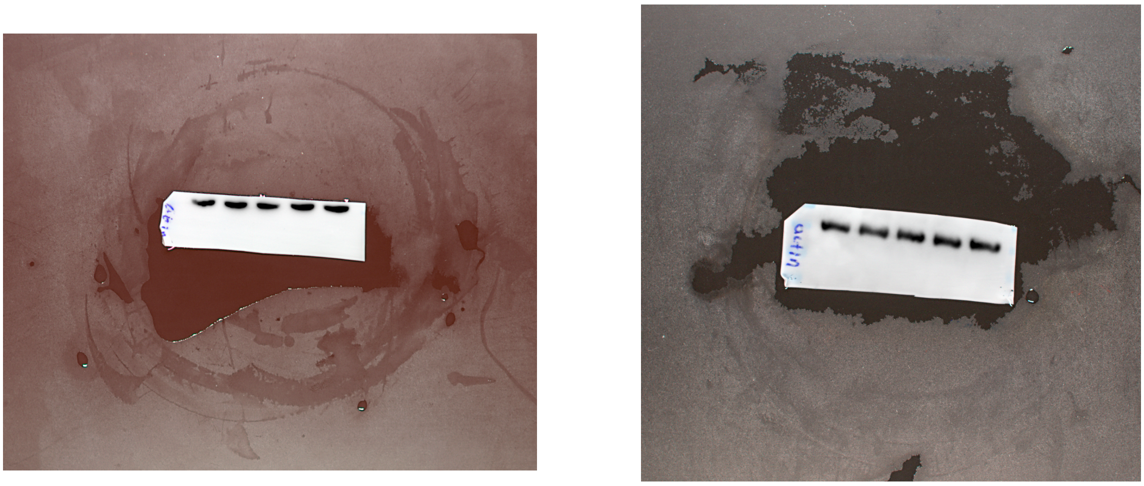

Figure7G-Actin

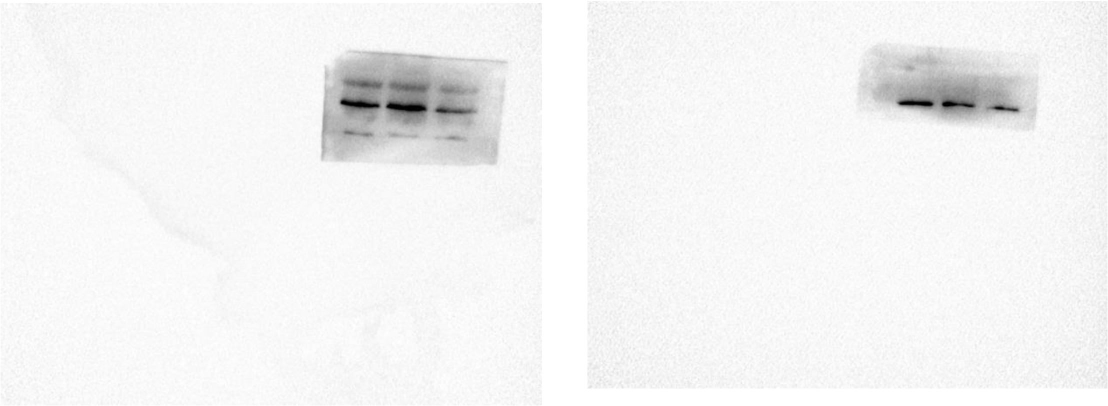

Figure7H-E-cadherin

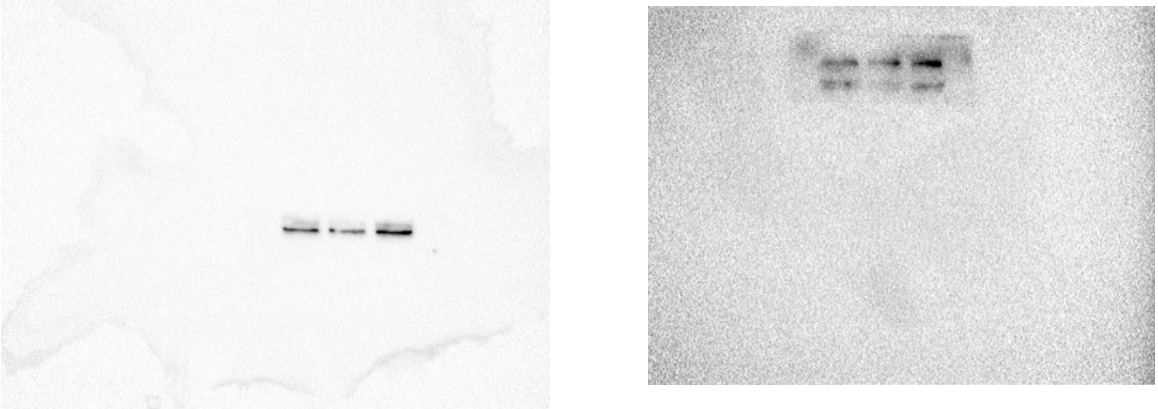

Figure7H-N-cadherin

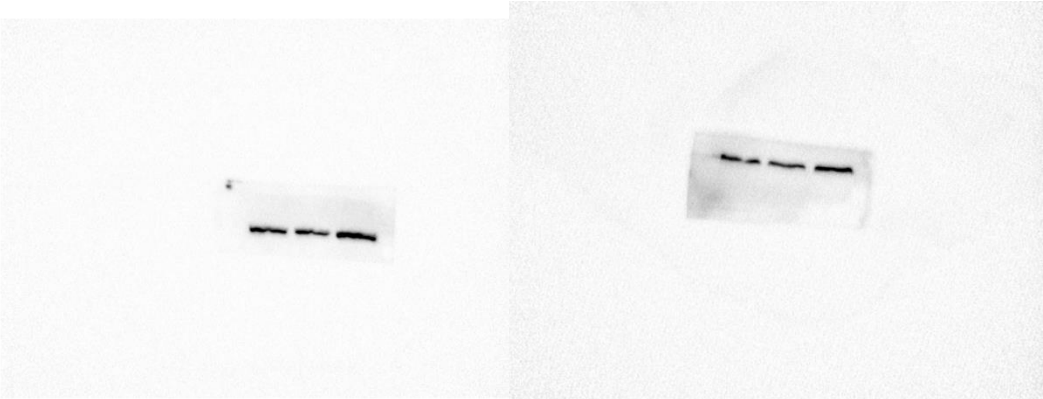

Figure7H-MMP-9

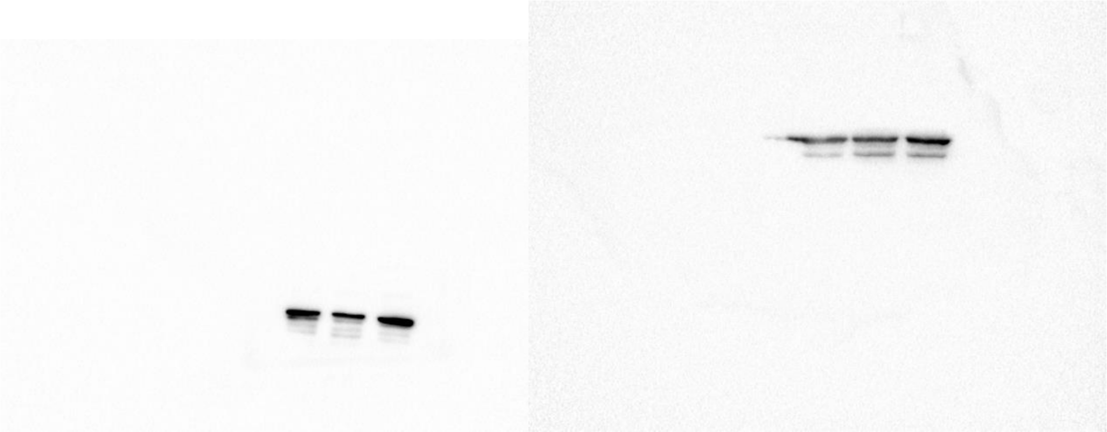

Figure7H-Vimentin

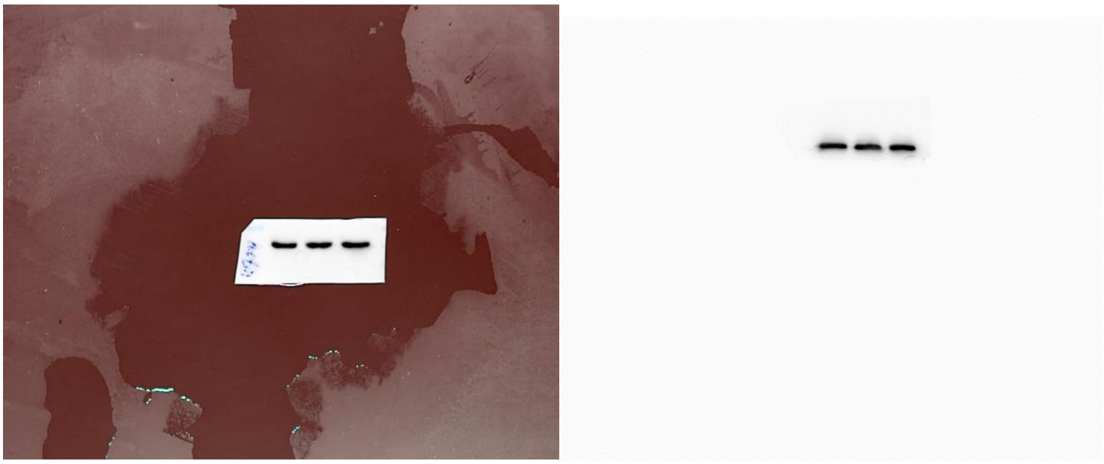

Figure7H-Actin

Figure8

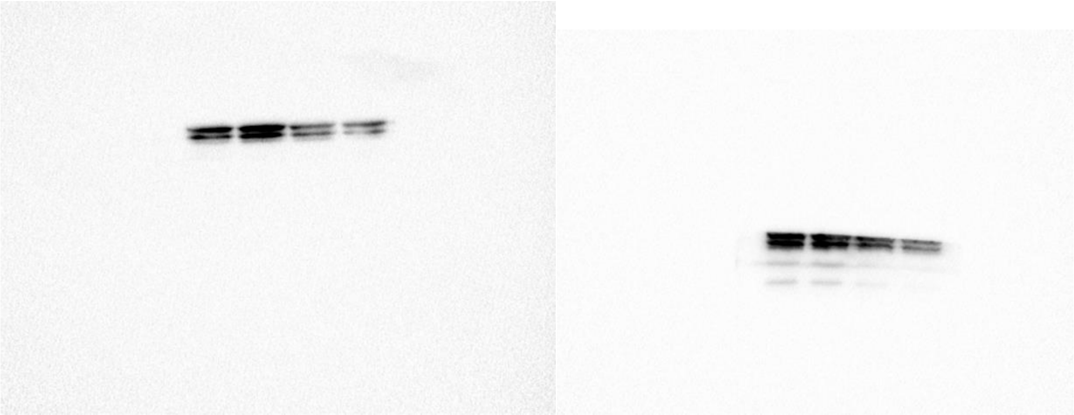

Figure8E-IGF2BP2

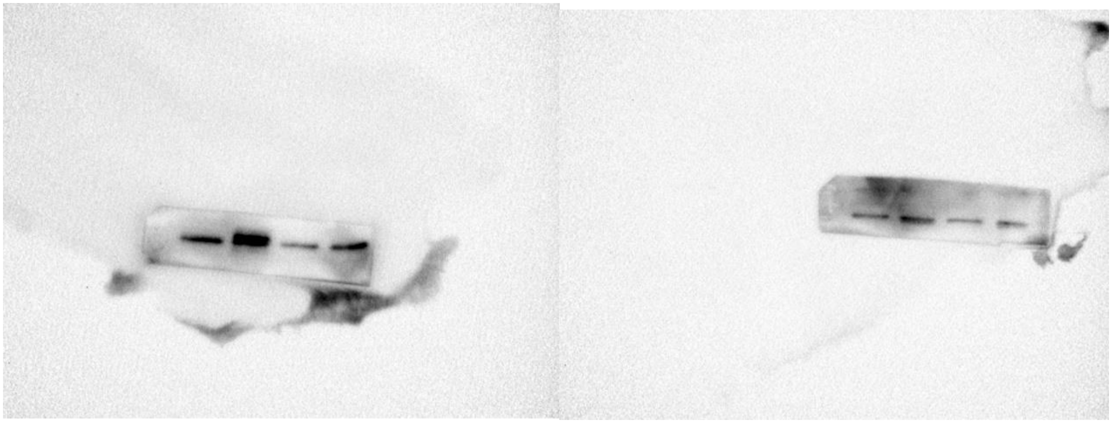

Figure8E-CDH12

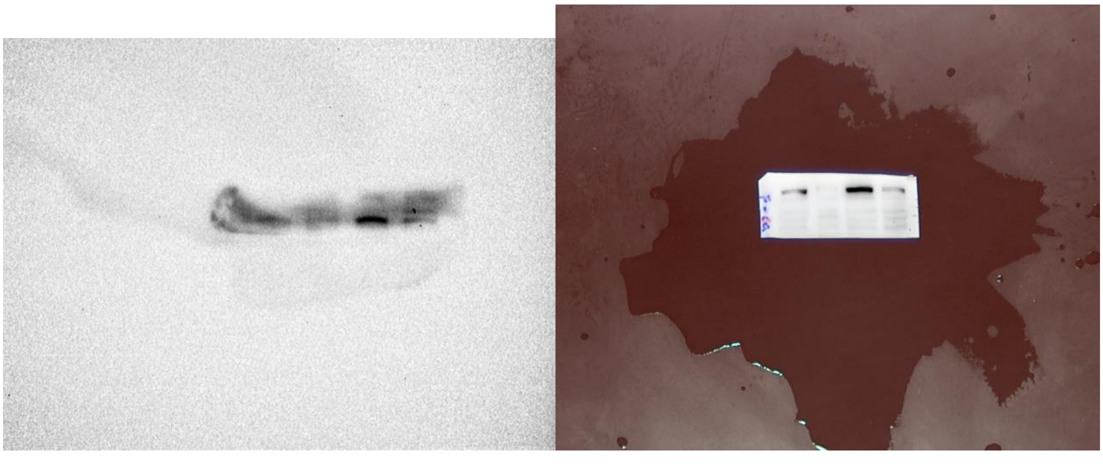

Figure8E-E-cadherin

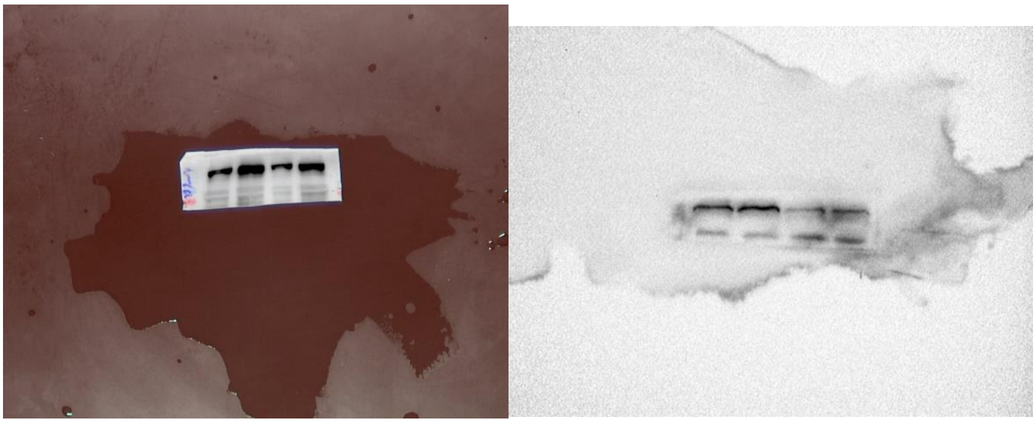

Figure8E-N-cadherin

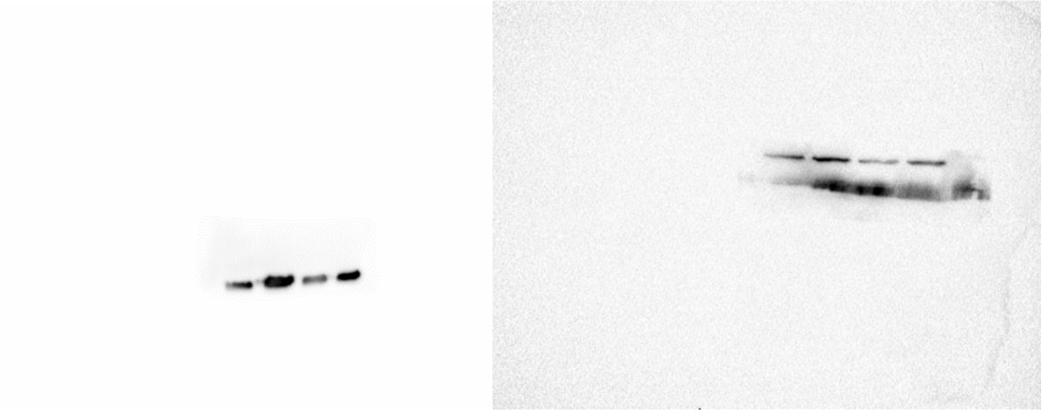

Figure8E-MMP-9

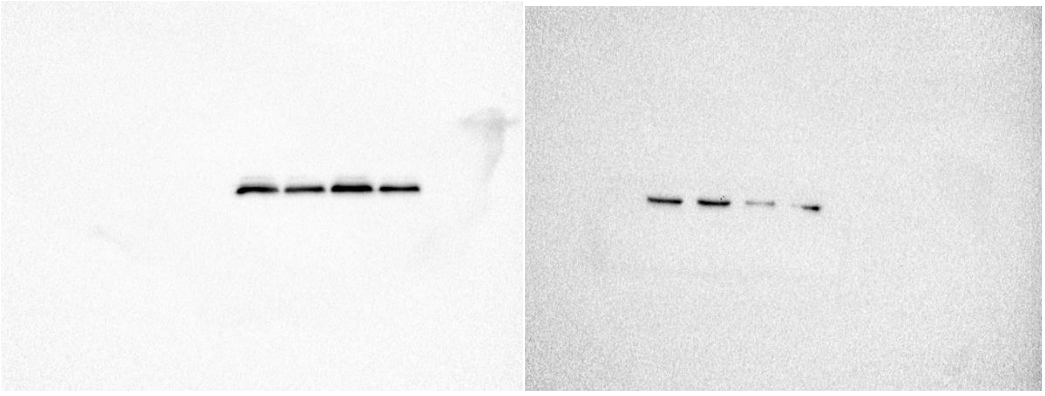

Figure8E-Vimentin

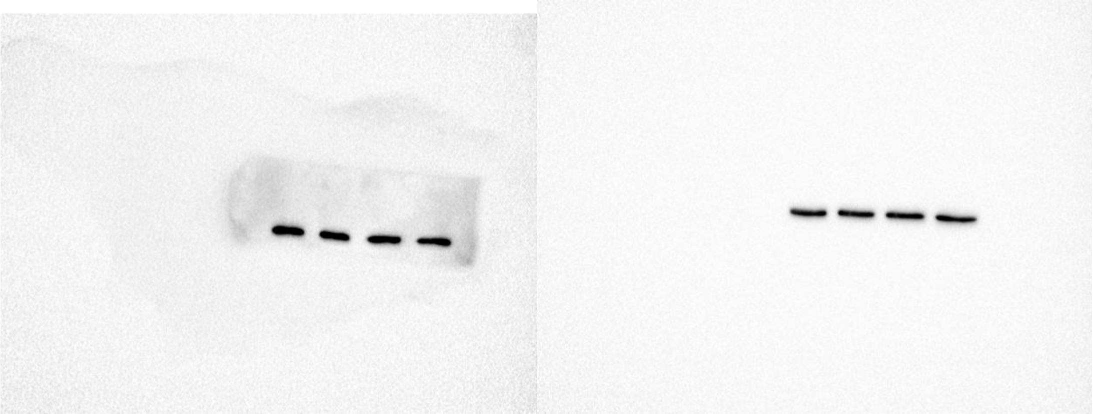

Figure8E-Actin

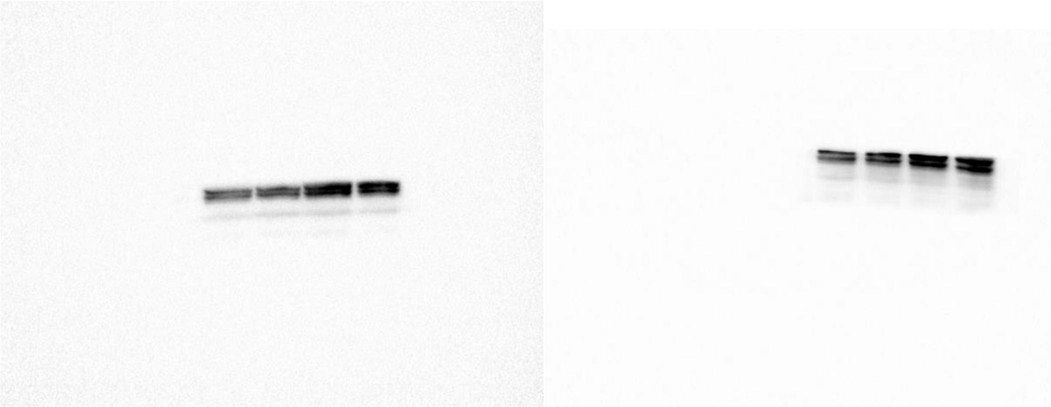

Figure8F-IGF2BP2

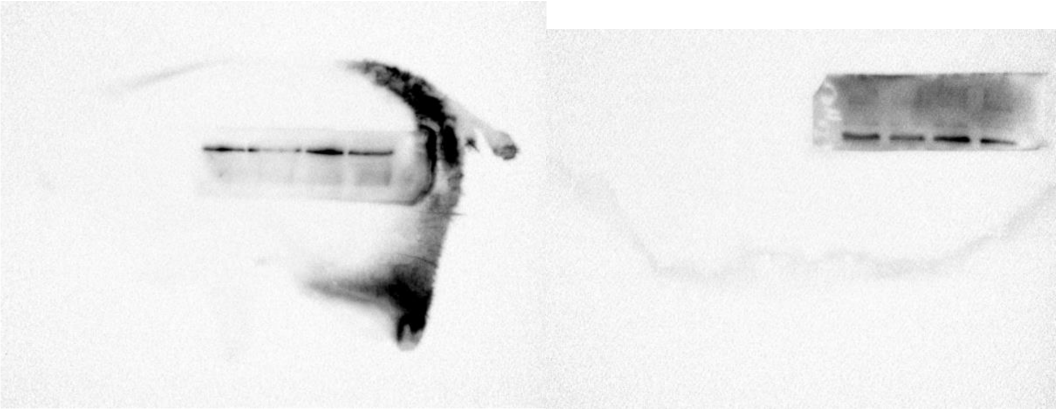

Figure8F-CDH12

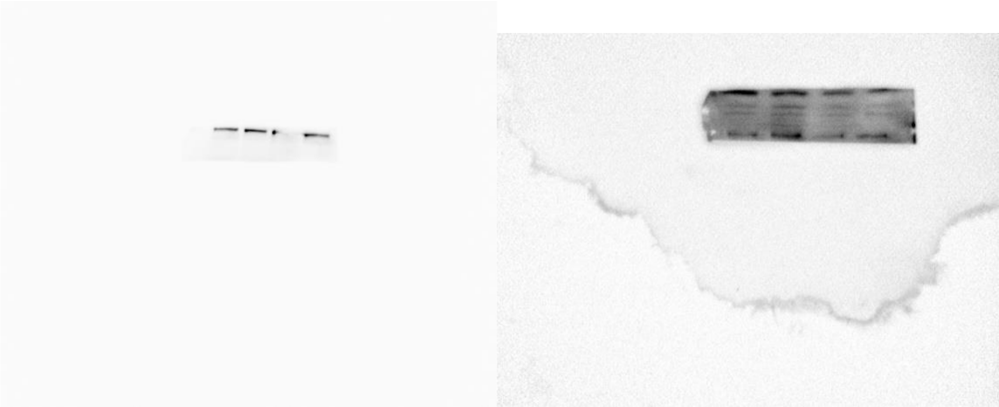

Figure8F-E-cadherin

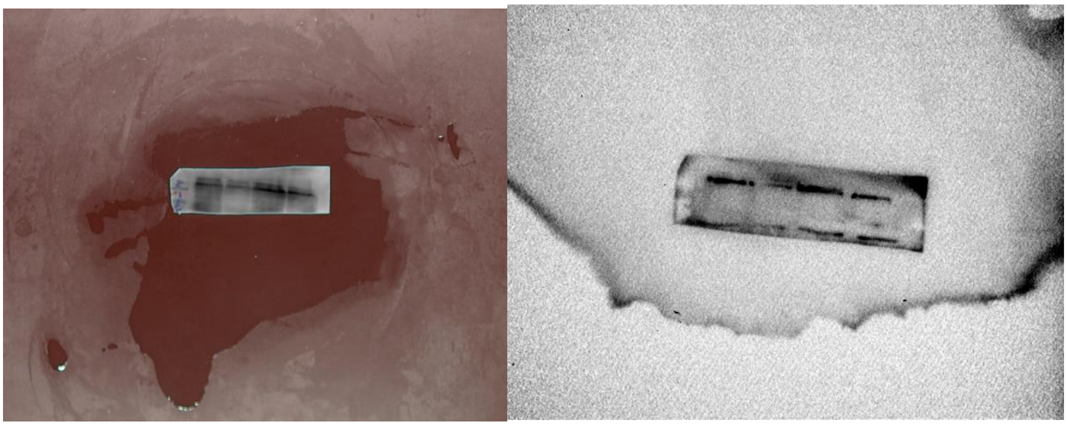

Figure8F-N-cadherin

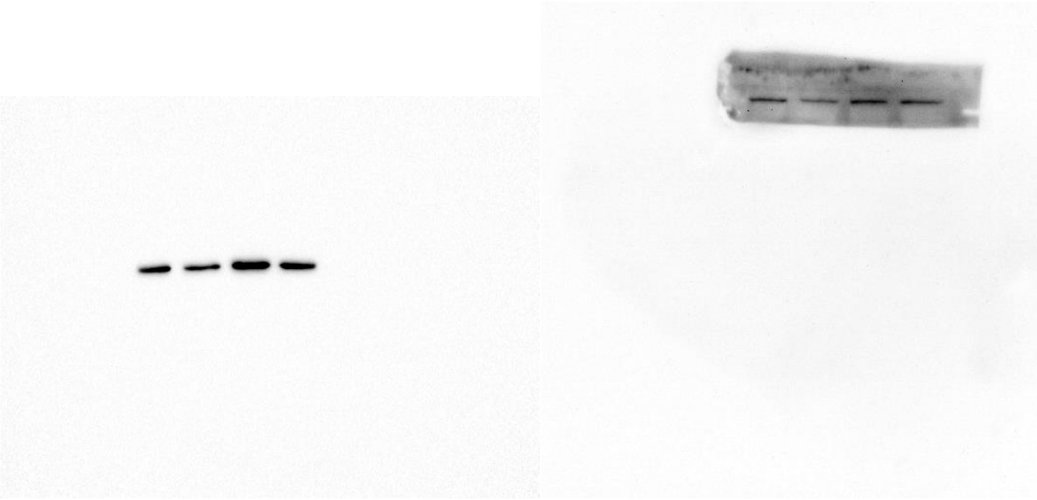

Figure8F-MMP-9

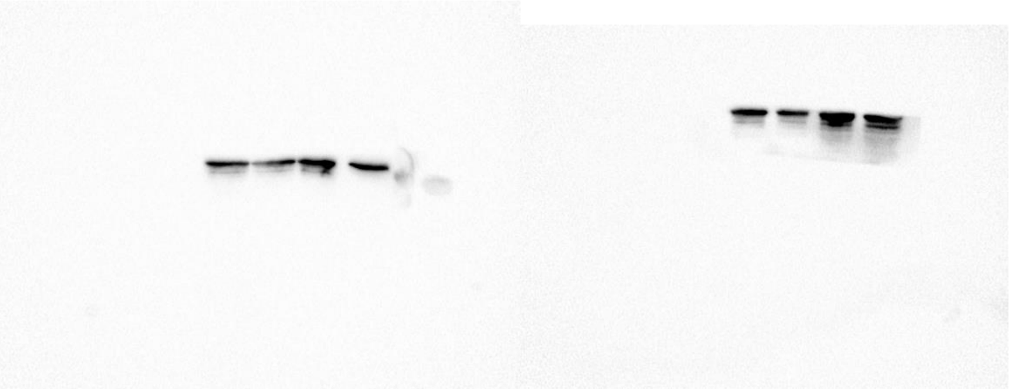

Figure8F-Vimentin

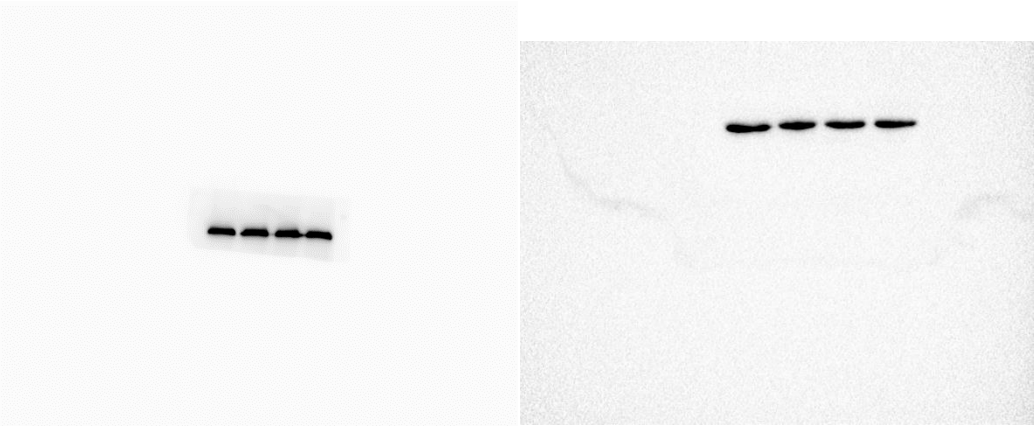

Figure8F-Actin
